# Dirhodium(II)-catalyzed [3 + 2] cycloaddition of *N*-arylaminocyclopropane with alkyne derivatives

**DOI:** 10.3762/bjoc.15.48

**Published:** 2019-02-25

**Authors:** Wentong Liu, Yi Kuang, Zhifan Wang, Jin Zhu, Yuanhua Wang

**Affiliations:** 1 Chengdu Institute of Organic Chemistry, Chinese Academy of Sciences, Chengdu 610046, China; 2 University of Chinese Academy of Sciences, Beijing 100049, China; 3 College of Chemistry, Sichuan University, Chengdu 610046, China

**Keywords:** [3 + 2], alkyne, cycloaddition, cyclopropanes, dirhodium catalysis, *N*-arylaminocyclopropanes

## Abstract

Dirhodium(II) complex-catalyzed [3 + 2] reactions between *N*-arylaminocyclopropanes and alkyne derivatives are described. The cycloaddition products proved to be versatile synthetic intermediates. *trans*-Cyclic β-amino acids and derivatives thereof can be conveniently synthesized using this cycloaddition protocol.

## Introduction

**Scheme 1:**
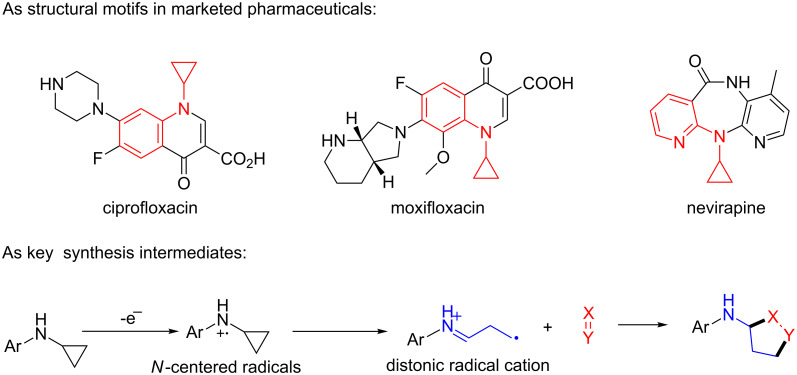
Applications of *N*-arylaminocyclopropanes.

*N*-Arylaminocyclopropanes **1** are important structural motifs for pharmaceuticals and are found especially in marketed fluoroquinolone antibiotics [1], such as ciprofloxacin and moxifloxacin (Scheme 1), and reverse transcriptase inhibitors [2], such as nevirapine. Meanwhile, since **1** contains a three-membered ring with high tension [3–6] and a nitrogen prone to single-electron oxidation, ring opening readily occurs followed by *N*-centered radical formation. The generated distonic radical cation can be further trapped by an alkene, alkyne, or triplet oxygen to initiate radical cyclization (Scheme 1) [7–15]. Thus, as key synthons, this class of molecules may play an important role in organic synthesis during construction of a series of aminocyclic compounds. In fact, the synthesis of **1** has always been a challenge [16–17]. Only recently an efficient synthesis method by arylation of cyclopropylamine has been developed and documented by Colacot et al. [16] and Stradiotto et al. [17], which has provided opportunities for further development of cycloaddition chemistry based on compound **1**. Zheng et al. [7] first reported on the [3 + 2] cycloaddition reaction of **1** with an alkene or alkyne mediated by visible light by the aid of the photocatalyst [Ru(bpz)_3_](PF_6_)_2_. Our group reported the metal catalyst itself, particularly the dinuclear rhodium complex (Rh_2_(II,II)), that efficiently catalyzes the ring opening of **1** to achieve cycloaddition with alkene substrates under an argon atmosphere [18]. During the reaction, no metal valence changes were observed. We proposed that Rh_2_(II,II) may coordinate with the nitrogen in **1** to decrease the bond dissociation energy of N–H bonds, which may be beneficial to N-centered radical formation. However, due to the characteristics of the radical reaction, the resulting cycloaddition product mixtures have a low diastereoisomeric ratio, which increases the difficulty of separation and limits applications. In view of this, we applied alkyne derivatives as cycloaddition partners to make the developed methodology more applicable, as well as to investigate the possibility of chiral ring construction.

## Results and Discussion

Initially, phenylcyclopropylamine (**1a**) and ethyl propiolate (**2a**) were selected as substrates to study the cycloaddition in dichloromethane (DCM) with 1 mol % starting catalyst loading under the previously reported conditions [18]. In the absence of a catalyst, no cycloaddition product **3a** formed (Table 1, entry 1). We then studied the effect of common rhodium catalysts on the reactions. When 1 mol % Rh(I) catalyst [Rh(CH_2_CH_2_)_2_Cl]_2_ was used (Table 1, entry 2), product **3a** was not detected. The commonly used Rh(III) catalyst [Rh(Cp*)Cl_2_]_2_ lead to a 72% yield of product **3a** (Table 1, entry 3). Next, the representative dirhodium(II) carboxylate such as Rh_2_(OAc)_4_, Rh_2_(TFA)_4_, Rh_2_(esp)_2_ and dirhodium(II) carboxamidate catalysts such as Rh_2_(cap)_4_, Rh_2_(5*S*, *R*-MenPY)_4_ were evaluated in the reaction. Rh_2_(OAc)_4_ created **3a** with a yield of 45% (Table 1, entry 4) and Rh_2_(TFA)_4_ improved the yield of **3a** to 52% (Table 1, entry 5). With the chelating catalyst Rh_2_(esp)_2_, the yield further increased to 68% (Table 1, entry 6). Although the carboxamidate type Rh_2_(cap)_4_ resulted in a lower yield (Table 1, entry 7), the yield raised to 61% when Rh_2_(5*S*, *R*-MenPY)_4_ [19–20] was applied as the catalyst (Table 1, entry 8). Next, 0.1 mol % catalytic loading was tested in the reactions to investigate the efficiency of the catalysts. Compared to the 1 mol % catalyst loading, the yield of **3a** dropped to 39 and 44% when Rh_2_(esp)_2_ and [Rh(Cp*)Cl_2_]_2_, respectively, were used (Table 1, entries 9, 10), and kept practically consistent with Rh_2_(5*S*, *R*-MenPY)_4_ (Table 1, entry 11). These results indicate that the catalytic efficiency of Rh_2_(5*S*, *R*-MenPY)_4_ was the best of all the screened catalysts. Further solvent screening studies found the yields of **3a** obtained in non-coordinating solvents, such as DCM, hexane, toluene, and 1,2-dichloroethane (DCE), and weak coordinating solvents, such as 1,2-dimethoxyethane (DME), were similar (Table 1, entries 11–15). DCE was the best solvent, leading to a 67% yield of **3a**. Due to axial coordination of dirhodium(II) for a strong coordination solvent, the yield of the resulting cycloaddition product in *N*,*N*-dimethylformamide decreased to only 33% (Table 1, entry 16). Regrettably, though Rh_2_(5*S*,*R*-MenPY)_4_ is a chiral catalyst, the obtained cycloaddition products are racemic.

**Table 1:** Catalyst screening and optimization of reaction conditions^a^.

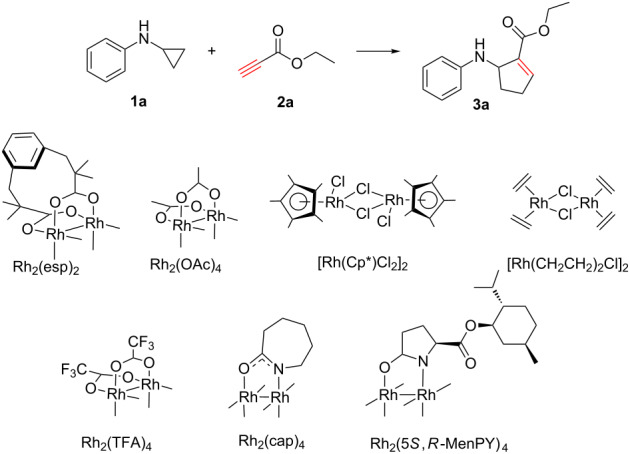			

Entry	Conditions	Solvent	Yield^b^ (%)

1	no catalyst	DCM	NR
2	[Rh(CH_2_CH_2_)_2_Cl]_2_	DCM	ND
3	[Rh(Cp*)Cl_2_]_2_	DCM	72
4	Rh_2_(OAc)_4_	DCM	45
5	Rh_2_(TFA)_4_	DCM	52
6	Rh_2_(esp)_2_	DCM	68
7	Rh_2_(cap)_4_	DCM	40
8	Rh_2_(5*S*,*R*-MenPY)_4_	DCM	61
9^c^	Rh_2_(esp)_2_	DCM	39
10^c^	[Rh(Cp*)Cl_2_]_2_	DCM	44
11^c^	Rh_2_(5*S*,*R*-MenPY)_4_	DCM	58
12^c^	Rh_2_(5*S*,*R*-MenPY)_4_	DCE	67
13^c^	Rh_2_(5*S*,*R*-MenPY)_4_	hexane	59
14^c^	Rh_2_(5*S*,*R*-MenPY)_4_	toluene	59
15^c^	Rh_2_(5*S*,*R*-MenPY)_4_	DME	60
16^c^	Rh_2_(5*S*,*R*-MenPY)_4_	DMF	33

^a^Reaction conditions: **1a** (0.5 mmol, 0.2 M in degassed solvent), **2a** (2.5 mmol), catalyst (1 mol %) under argon at room temperature for 24 h unless otherwise noted. ^b^Isolated yield. ^c^0.1 mol % of catalyst. esp = α,α,α’,α’-tetramethyl-1,3-benzenedipropionate, cap = caprolactamate, 5*S*,*R*-MenPY = (*S*)-(1*R*,2*S*,5*R*)-2-isopropyl-5-methylcyclohexyl 2-oxopyrrolidine-5-carboxylate, NR = no reaction, ND = not detected.

According to previous reports [16], after a series of arylcyclopropylamines **1** with different substituents were synthesized, the scope of **1** was then explored and the results are shown in Table 2. The data suggest that the electronic effect on the aromatic ring of **1** influences the results of the reactions. Compound **1** with electron-donating groups, such as methoxy, *tert*-butyl, and methyl groups, all reacted smoothly to produce products with good yields (Table 2, entries 1–3). The cyclization of substrate **1** containing electron-withdrawing groups at the aromatic substuituent proceeded slowly, producing considerably lower yields of the corresponding products. For instance, multiple-substituted compound **1** (with a 3,5-disubstitued trifluoromethyl **1f**) generated product **3f** with a yield of 36% (88% brsm) after 24 h (Table 2, entry 5), but 3,5-dimethyl compound **1d** afforded the desired product **3d** with an excellent yield of 91% (Table 2, entry 3). We reasoned the electron-withdrawing groups on the arene increased the nitrogen–hydrogen bond dissociation energy (BDE_N–H_) of compound **1** [21], decreasing the rate of ring opening. Substrates **1i**,**j** containing hindered substituents lead to low conversion of the substrates, producing only poor yields of products **3i**,**j** (Table 2, entries 8 and 9), which implied that steric hindrance greatly influenced the reactions.

**Table 2:** Substrate scope of *N*-arylaminocyclopropanes^a^.

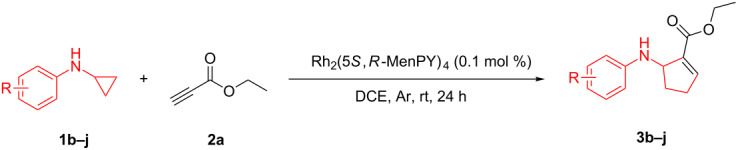					

Entry	Substrate		Product		Yield^b^

1	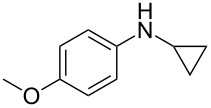	**1b**	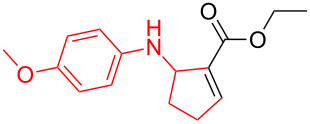	**3b**	59%
2	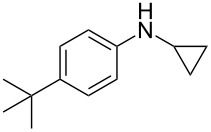	**1c**	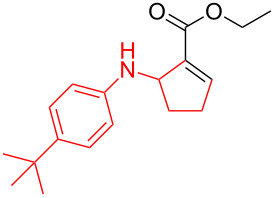	**3c**	78%
3	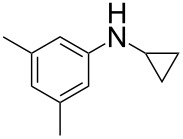	**1d**	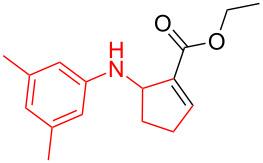	**3d**	91%
4	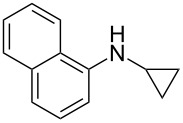	**1e**	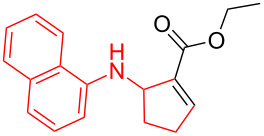	**3e**	85%
5	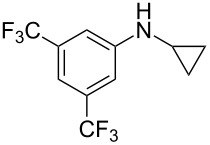	**1f**	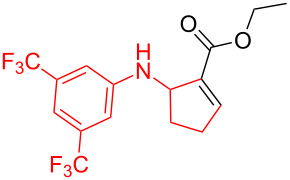	**3f**	36% (88% brsm)
6	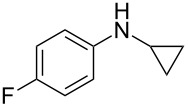	**1g**	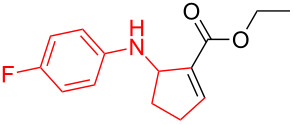	**3g**	67%
7	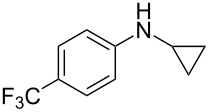	**1h**	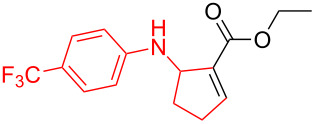	**3h**	24% (52% brsm)
8	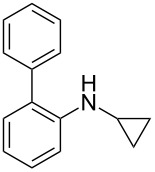	**1i**	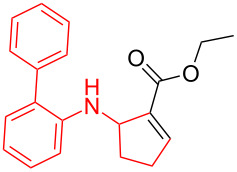	**3i**	20% (67% brsm)
9	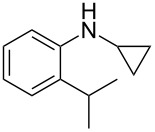	**1j**	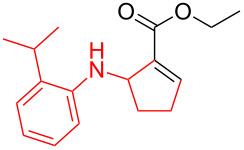	**3j**	37% (65% brsm)

^a^Reaction conditions: **1b**–**j** (1 mmol, 0.2 M in degassed solvent), **2a** (5 mmol), catalyst (0.1 mol %) at room temperature for 24 h unless otherwise noted. ^b^Isolated yield; brsm = based on recovered starting material.

Next, we surveyed the different alkyne substrates **2** for cycloaddition under optimized reaction conditions. The terminal alkynes with electron-withdrawing groups reacted smoothly to produce the desired products, while alkyl-substituted terminal aldehydes, such as pent-1-yne (**2b**, Table 3, entry 1), did not produce the cycloaddition product [22–24]. *tert*-Butyldimethylsilyl-protected propargyl alcohol **2c** had a greatly reduced reactivity (Table 3, entry 2) and the obtained yield was less than 10%. For aromatic terminal alkynes, moderate yields (Table 3, entries 3–6) were obtained regardless of the electron-donating or electron-withdrawing groups, indicating the electronic effect on the aromatic ring of **2** had little effect on the results of the reactions. Due to the decrease in the activity of the alkyne, the conjugated 2,4-hexadiyne (**2h**) produced a poor yield of only 31% (Table 3, entry 7). To study the chemical selectivity of the reaction, we examined conjugated alkyne substrates. For 2-methylbut-1-en-3-yne (**2i**) containing both terminal alkenes and alkynes, the activity of the olefin was greater than that of the alkyne, which favors alkene cycloaddition products with a ratio of 89:11. The observed selectivity of product **4i** is similar to that previously reported [11] (Table 3, entry 8), indicating the results of this type of reaction are mainly determined by the stability of the free radical intermediate during the reaction. Further investigation revealed cycloaddition preferentially proceeded with the terminal alkene or alkyne. Product **4j** was obtained with a 41% yield due to cycloaddition with an olefin group in **2j** (Table 3, entry 9). When **2k** was applied in the reaction, the addition product was obtained with a yield of 44% with the alkyne group being involved in the reaction (Table 3, entry 10). The reaction with **2l** mainly produced 1,3-diene product **4l** with a yield of 40% (Table 3, entry 11). In addition, we also examined some alkynes containing heterocycles, such as 3-ethynylpyridine and 2-ethynylthiophene. Because the heteroatoms were axially tightly coordinated to the dirhodium(II) catalyst, coordination of the cyclopropylamine with the dirhodium(II) catalyst was inhibited, resulting in no cycloaddition reaction occurring.

**Table 3:** Substrate scope of alkyne derivatives^a^.

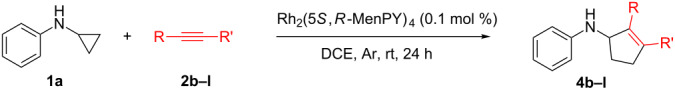					

Entry	Substrate		Product		Yield^b^(%)

1	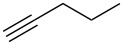	**2b**	N.D.	**4b**	N.R.
2	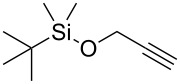	**2c**	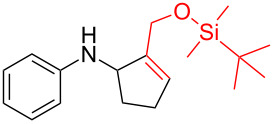	**4c**	<10
3	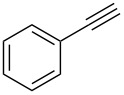	**2d**	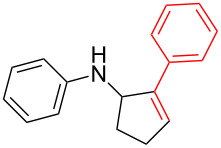	**4d**	65
4	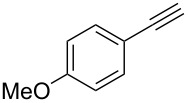	**2e**	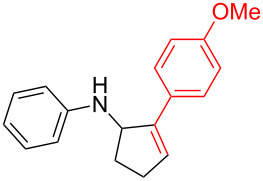	**4e**	64
5	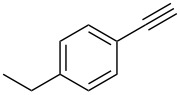	**2f**	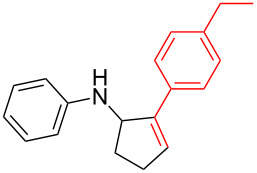	**4f**	65
6	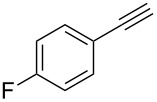	**2g**	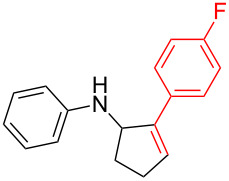	**4g**	64
7	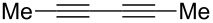	**2h**	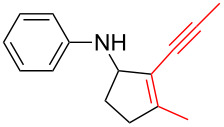	**4h**	31
8^c^	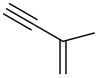	**2i**	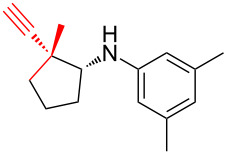	**4i**^d,e^	45 (17:83)^f^
9	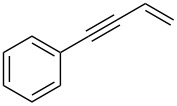	**2j**	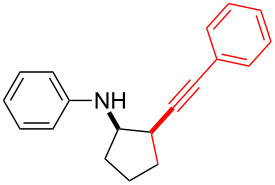 , 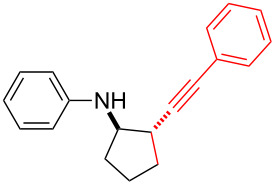	**4j**	41 (30:70)^f^
10	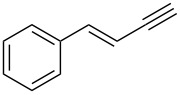	**2k**	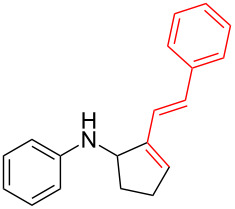	**4k**	44
11^c^	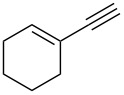	**2l**	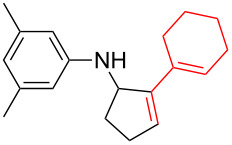	**4l**^d^	40

^a^Reaction conditions: **1a** (1 mmol, 0.2 M in degassed solvent), **2b**–**l** (5 mmol), catalyst (0.1 mol %) at room temperature for 24 h unless otherwise noted. ^b^Isolated yield. ^c^*N*-Arylaminocyclopropane **1d** was used instead of **1a**. ^d^Major isomer shown. ^e^Isomer ratio 89:11. ^f^Diastereoisomeric ratios (*cis/trans*) were determined by ^1^H NMR spectroscopy of the crude products.

**Scheme 2:**
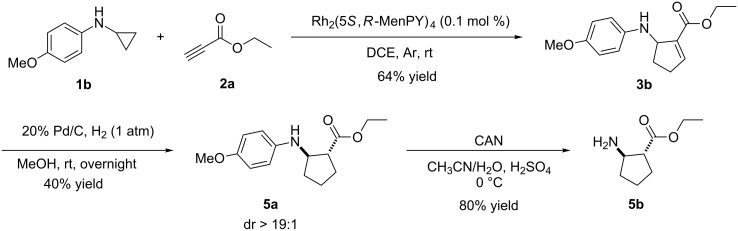
Synthesis of *trans-*ethyl 2-aminocyclopentanecarboxylate.

To explore the synthetic practicality of this transformation, racemic compound **3b** resulting from **1b** and **2a** was reduced using hydrogen and palladium on carbon (Scheme 2). It is interesting to note that only *trans*-**5a** was obtained and no *cis* product formed. After further removal of the *para*-methoxyphenyl (PMP) group using ammonium cerium nitrate (CAN), the cyclic β-amino acid ester **5b** was obtained with a yield of 80%. Cyclic β-amino acids and derivatives have good bioactivity and are widely used as key synthetic intermediates in biomedical research [25–29]. Thus, based on this cycloaddition protocol, a convenient strategy can be established to synthesize *trans*-cyclic β-amino acids.

**Scheme 3:**
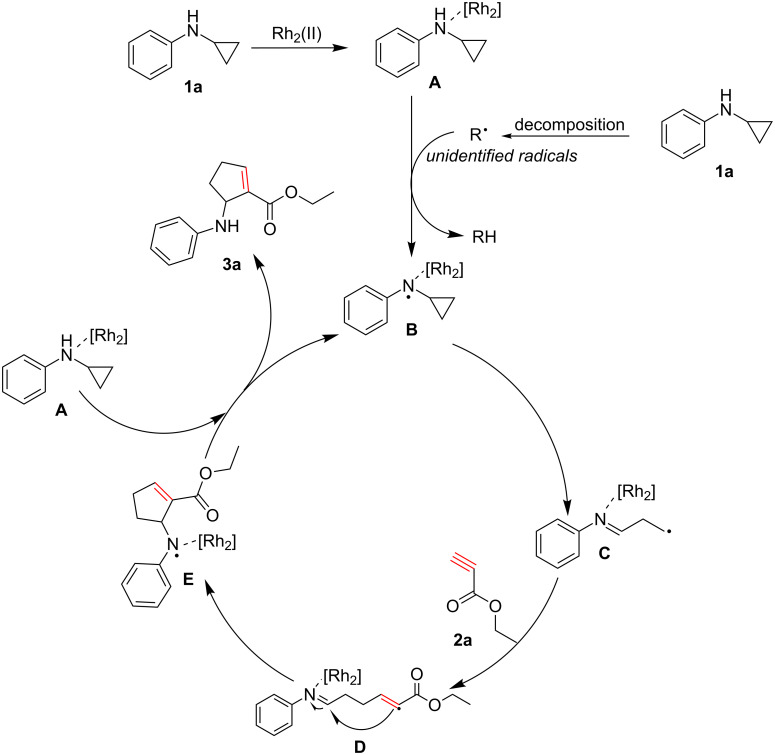
Proposed mechanism.

The mechanism of the [3 + 2] cycloaddition reaction of **1a** and **2a** is similar to that previously reported [18] (Scheme 3). The distonic radical cation **C** resulting from cyclopropane ring opening reacts with alkyne substrate **2a** generating radical **D**. The intermediate radical **D** yielded **E** through intramolecular radical addition. After hydrogen atom transfer (HAT) from complex **A**, the desired product is obtained with regeneration of the N-centered radical **B**, which continues to catalyzing the reaction.

## Conclusion

In conclusion, we report on the [3 + 2] cycloaddition reaction catalyzed by dirhodium(II) based on arylcyclopropylamine, which broadens the scope of this method to the alkynyl group. This study demonstrated that this cycloaddition method has potential synthetic practicality by providing a convenient way to synthesize *trans*-cyclic β-amino acid derivatives. Further application of this method with other cycloaddition partners and asymmetric synthesis of the chiral ring with the help of chiral auxiliaries are currently underway.

## Experimental

**General procedure for the [3 + 2] annulation of cyclopropylanilines:** An oven-dried Schlenk tube equipped with a stirring bar was charged with Rh_2_(5*S*,*R*-MenPY)_4_ (0.1 mol %), alkyne (5.0 mmol), and dry DCE (5 mL). The tube was degassed through three freeze–pump–thaw cycles. After evacuating and backfilling the tube with argon three times, cyclopropylamine (1 mmol) was added. The reaction mixture was stirred at room temperature for 24 hours. After the reaction was complete, the mixture was concentrated and the residue was purified by flash chromatography to obtain the desired allylic amine.

## Supporting Information

File 1 Experimental procedures, compound characterization, and NMR spectra.
