# Supplementary material for: Dirhodium(II)-catalyzed [3 + 2] cycloaddition of N-arylaminocyclopropane with alkyne derivatives
Source: Beilstein J Org Chem. 2019 Feb 25;15:542–50. doi: 10.3762/bjoc.15.48 (PMC6404392; doi:10.3762/bjoc.15.48)
Supplement: File 1 — Experimental procedures, compound characterization, and NMR spectra. [file Beilstein_J_Org_Chem-15-542-s001.pdf]

BEILSTEIN JOURNAL OF ORGANIC CHEMISTRY

Supporting Information

for

Dirhodium(II)-catalyzed [3 + 2] cycloaddition of *N*-arylaminocyclopropane with alkyne derivatives

Wentong Liu, Yi Kuang, Zhifan Wang, Jin Zhu and Yuanhua Wang

Beilstein J. Org. Chem. **2019,** *15,* 542–550. doi:10.3762/bjoc.15.48

Experimental procedures, compound characterization, and NMR spectra

License and Terms: This is a supporting information file under the terms of the Creative Commons Attribution License (http://creativecommons.org/licenses/by/4.0). Please note that the reuse, redistribution and reproduction in particular requires that the authors and source are credited.
The license is subject to the *Beilstein Journal of Organic Chemistry* terms and conditions: (https://www.beilstein-journals.org/bjoc)

Table of contents

1	General considerations	S2
2	Experimental procedures and spectroscopic data	S2
2.1	Synthesis of Rh_2(5*S*,*R*-MenPY)$_4$	S2
2.2	Synthesis of [Pd(allyl)(brettphos)]OTf	S3
2.3	Synthesis of *N*-aryl aminocyclopropanes	S3
2.4	Characterization of [3 + 2] cycloaddition products	S6
3	Synthesis of *trans*-ethyl 2-aminocyclopentanecarboxylate	S15
4	References	S16
5	^{1}H and ^{13}C NMR spectra of compounds	S17

1. General considerations

All reactions were carried out under a nitrogen or argon atmosphere unless otherwise stated. Commercially available metal catalysts and reagents were purchased from Adamas-beta, Sigma-Aldrich, Alfa-Aesar or TCI. Rh_2(5*S*,*R*-MenPY)$_4$[1] was prepared as described in the literature. **2j**[2] and **2k**[3] were prepared as described in the literature. All the solvents, unsaturated esters, and alkynes were distilled or purified before using. column chromatography was carried out with silica gel (200–300 mesh) using hexane and ethyl acetate as eluent.

NMR spectra were recorded on a Bruker AV II-400 spectrometer. The ^{1}H NMR (400 MHz) chemical shifts were recorded relative to $CDCl_3$, DMSO-d_6 as the internal reference ($CDCl_3$: δ_H = 7.26 ppm; DMSO-d_6: δ_H = 2.50 ppm). The ^{13}C NMR (100 MHz) chemical shifts were given using $CDCl_3$ or DMSO-d_6 as the internal standard ($CDCl_3$: δ_C = 77.16 ppm; DMSO-d_6: δ_C = 39.52 ppm).

2. Experimental procedures and spectroscopic data

2.1 Synthesis of Rh_2(5S,R-MenPY)$_4$

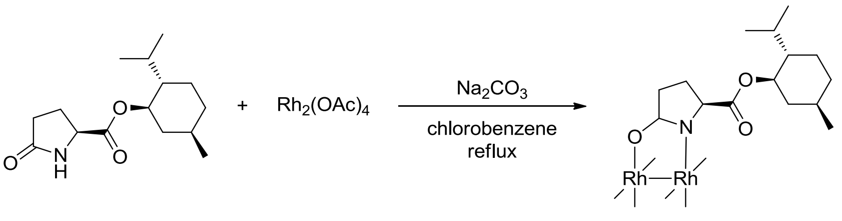

Rh_2(5*S*,*R*-MenPY)$_4$ was synthesized according to the literature.[1] Dirhodium(II) acetate (800 mg, 1.8 mmol), (*S*)-(1*R*,2*S*,5*R*)-2-isopropyl-5-methylcyclohexyl 2-oxopyrrolidine-5-carboxylate (3.50 g, 13.1 mmol) and chlorobenzene (80 mL), were mixed in around bottom flask fitted with a Soxhlet extraction apparatus into which was placed a celluose thimble containing 2:1 Na_2CO_3/sand. The resulting mixture was heated at reflux for 16 hours. After cooling to room temperature, the solvent was removed under vacuo, and the resulting deep red solid was chromatographed on BAKERBOND-CN (40 mm reverse phase silica) eluting with MeOH, initially removed unreacted ligand (brown band), then the desired catalyst, which elutes as a red band: yield 1.31 g (57% yield) of a blue solid. ^{1}H NMR (400 MHz, $CDCl_3$): δ 4.75-4.56 (m, 4H), 4.16-4.10 (m, 2H), 4.10-4.03 (m, 2H), 2.70-2.50 (m, 4H), 2.30-1.77 (m, 20H), 1.75-1.62 (m, 8H), 1.55-1.34 (m, 8H), 1.10-0.70 (m, 48H).

2.2 Synthesis of [Pd(allyl)(brettphos)]OTf

[Pd(allyl)(brettphos)]OTf was synthesized according to the literature.[4] A dry Schlenk flask equipped with a Teflon-coated magnetic stirring bar was charged with $[(allyl)PdCl]_2$ (183 mg, 0.5 mmol) followed by AgOTf (257 mg, 1.0 mmol). The flask was fitted with a rubber septum, evacuated, and backfilled with nitrogen. This evacuation/nitrogen backfill cycle was repeated two additional times. Solvent (10 mL of THF) was added, and the reaction mixture was stirred at room temperature for 30 min while protected from light. A second dry Schlenk flask was equipped with a magnetic stirring bar, fitted with a Schlenk frit, and charged with BrettPhos (537 mg, 1.0 mmol). The flask was fitted with a rubber septum, and it was evacuated and backfilled with nitrogen. This evacuation/nitrogen backfill cycle was repeated two additional times. The solution from the first Schlenk flask was transferred through a millipore filter (to remove AgCl) into the second Schlenk flask containing the ligand, rinsing with 5 mL of additional solvent (THF). This mixture was stirred at room temperature for 2 h. Hexanes (450 mL) was then added to the mixture to fully precipitate the product. The solid materials were then collected by suction filtration, washed with additional hexanes, and dried in vacuo to give 734 mg (86%) of the title compound as a yellow solid. ^{1}H NMR(400 MHz, $CDCl_3$): δ 7.46 (d, *J* = 1.7 Hz, 1H), 7.35 (d, *J* = 1.7 Hz, 1H), 7.11-7.07 (m, 1H), 7.02-6.96 (m, 1H), 5.61-5.50 (m, 1H), 4.26 (d, *J* = 6.6 Hz, 1H), 3.95 (s, 3H), 3.57-3.50 (m, 1H), 3.41 (s, 3H), 3.07-2.97 (m, 1H), 2.88-2.76 (m, 1H), 2.71 (d, *J* = 12.5 Hz, 1H), 2.65-2.53 (m, 1H), 2.48-2.38 (m, 2H), 2.28-2.20 (m, 1H), 2.08-1.96 (m, 2H), 1.92-1.05 (m, 32H), 1.03-0.80 (m, 7H).

2.3 Synthesis of N-aryl aminocyclopropane

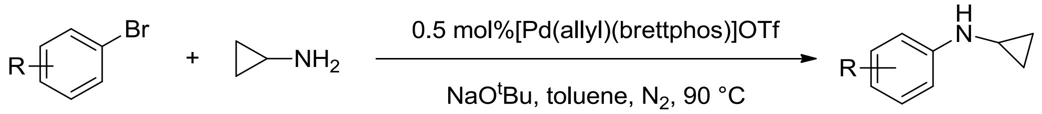

2.3.1 General procedure:

The *N*-aryl aminocyclopropanes were synthesized according to the literature.[5] An oven-dried Schlenk tube equipped with a stirring bar was charged with [Pd(allyl)(brettphos)]OTf (0.025 mmol, 0.5 mol %), and NaO*t*-Bu (7.5 mmol). The tube was evacuated and backfilled with nitrogen for three times. The amine (7.5 mmol), aryl bromine (5.0 mmol), and anhydrous toluene (40 mL) were added

sequentially via syringe. The tube was placed in a preheated oil bath and the contents were stirred for the indicated time. The tube was then removed from the oil bath and allowed to cool to room temperature. The reaction mixture was diluted with 10 mL of dichloromethane and filtered through a pad of Celite. The solution was concentrated in vacuo, and the residue was purified on silica gel using n-hexane/ethyl acetate as eluent.

***N*-Cyclopropylaniline (1a)[6]:** The general procedure was followed, and flash chromatography (*n*-hexane/EtOAc = 30/1) afforded 386 mg (58% yield). Colorless oil. ^{1}H NMR (400 MHz, $CDCl_3$): 7.22-7.18 (m, 2H), 6.82-6.78 (m, 2H), 6.76-6.72 (m, 1H), 4.18 (br s, 1H), 2.45-2.40 (m, 1H), 0.75-0.70 (m, 2H), 0.53-0.50 (m, 2H).

***N*-Cyclopropyl-4-methoxyaniline (1b)[7]:** The general procedure was followed, and flash chromatography (*n*-hexane/EtOAc = 10/1) afforded 375 mg (46% yield). Colorless oil. ^{1}H NMR (400 MHz, $CDCl_3$): δ 6.83-6.75 (m, 4H), 3.99 (br s, 1H), 3.77 (s, 3H), 2.40 (m, 1H), 0.75-0.68 (m, 2H), 0.53-0.48 (m, 2H).

4-(*tert*-Butyl)-*N*-cyclopropylaniline (1c): The general procedure was followed, and flash chromatography (*n*-hexane/EtOAc = 30/1) afforded 511 mg (54% yield). Colorless oil. ^{1}H NMR (400 MHz, $CDCl_3$) δ 7.24 (dd, J = 10.5, 2.1 Hz, 2H), 6.77 (d, J = 8.4 Hz, 2H), 4.09 (br s, 1H), 2.42 (m, 1H), 1.30 (s, 9H), 0.74-0.68 (m, 2H), 0.54-0.48 (m, 2H). ^{13}C NMR (100 MHz, $CDCl_3$) δ 146.44, 140.62, 126.01, 112.97, 33.99, 31.71, 25.51, 7.45.

3,5-Dimethyl-*N*-cyclopropylaniline (1d)[8]: The general procedure was followed, and flash chromatography (*n*-hexane/EtOAc = 30/1) afforded 508 mg (63% yield). Colorless oil. ^{1}H NMR (400 MHz, $CDCl_3$): δ 6.47 (s, 2H), 6.45 (s, 1H), 4.10 (s, 1H), 2.47-2.41 (m, 1H), 2.30 (s, 6H), 0.78-0.72 (m, 2H), 0.56-0.50 (m, 2H).

***N*-cyclopropylnaphthalen-1-amine (1e)[9]:** The general procedure was followed, and flash chromatography (*n*-hexane/EtOAc = 30/1) afforded 660 mg (72% yield). White solid. ^{1}H NMR (400 MHz, $CDCl_3$) δ 7.88-7.82 (m, 1H), 7.80-7.72 (m, 1H), 7.55-7.40 (m, 3H), 7.36-7.30 (m, 1H), 7.17-7.08 (m, 1H), 4.90 (br s, 1H), 2.64-2.58 (m, 1H), 0.91-0.85 (m, 2H), 0.70-0.63 (m, 2H).

***N*-cyclopropyl-3,5-bis(trifluoromethyl)aniline (1f)**[10]**:** The general procedure was followed, and flash chromatography (*n*-hexane/EtOAc = 30/1) afforded 942 mg (70% yield). White solid. ^{1}H NMR (400 MHz, $CDCl_3$): δ 7.18 (s, 1H), 7.12 (s, 2H), 4.56 (br s, 1H), 2.51-2.44 (m, 1H), 0.87-0.81 (m, 2H), 0.58-0.52 (m, 2H).

***N*-cyclopropyl-4-fluoroaniline (1g):** The general procedure was followed, and flash chromatography (*n*-hexane/EtOAc = 30/1) afforded 476 mg (63% yield). Colorless oil. ^{1}H NMR (400 MHz, $CDCl_3$) δ 6.91 (t, *J* = 8.8 Hz, 2H), 6.73 (dd, *J* = 9.1, 4.5 Hz, 2H), 4.09 (br s, 1H), 2.43-2.36 (m, 1H), 0.76-0.69 (m, 2H), 0.53-0.48 (m, 2H). ^{13}C NMR (100 MHz, $CDCl_3$) δ 157.38, 155.04 (s), 145.10 (d, *J* = 1.8 Hz), 115.61 (d, *J* = 22.3 Hz), 113.92 (d, *J* = 7.4 Hz), 25.83, 7.48.

***N*-cyclopropyl-4-(trifluoromethyl)aniline (1h)**[11]**:** The general procedure was followed, and flash chromatography (*n*-hexane/EtOAc = 30/1) afforded 942 mg (70% yield). White solid. ^{1}H NMR (400 MHz, CDCl3): δ 7.42 (d, *J* =8.6 Hz, 1H), 6.79 (d, *J* =8.5 Hz, 1H), 4.45 (br s, 1H), 2.50-2.42 (m, 1H), 0.82-0.76 (m, 2H), 0.56-0.51 (m, 2H).

***N*-cyclopropyl-[1,1'-biphenyl]-2-amine (1i)**[11]**:** The general procedure was followed, and flash chromatography (*n*-hexane/EtOAc = 30/1) afforded 638 mg (61% yield). White solid. ^{1}H NMR (400 MHz, $CDCl_3$): δ 7.50-7.40 (m, 4H), 7.39-7.34 (m, 1H), 7.33-7.28 (m, 1H), 7.18 (dd, *J* = 8.2, 1.1 Hz, 1H), 7.12 (dd, *J* = 7.4, 1.6 Hz, 1H), 6.84 (td, *J* = 7.4,1.2 Hz, 1H), 4.43 (br s, 1H), 2.43-2.37 (m, 1H), 0.76-0.71 (m, 2H), 0.53-0.48 (m, 2H).

***N*-cyclopropyl-2-isopropylaniline (1j)**[12]**:** The general procedure was followed, and flash chromatography (*n*-hexane/EtOAc = 100/1) afforded 456 mg (52% yield). Colorless oil. ^{1}H NMR (400 MHz, $CDCl_3$) δ 7.21-7.07 (m, 3H), 6.84-6.74 (m, 1H), 4.21 (s, 1H), 2.80 (p, *J* = 6.8 Hz, 1H), 2.45 (tt, *J* = 6.4, 3.6 Hz, 1H), 1.29-1.23 (m, 6H), 0.82-0.73 (m, 2H), 0.61-0.52 (m, 2H).

2.4Characterization of [3 + 2] cycloaddition products

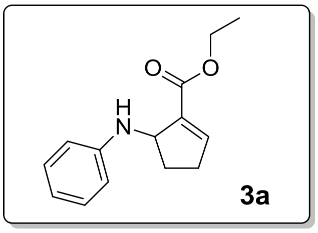

Ethyl 5-(phenylamino)cyclopent-1-enecarboxylate (3a)[13]**:** The general procedure was followed, and flash chromatography (*n*-hexane/EtOAc = 50/1) afforded 155 mg (67% yield). Light yellow oil. ^{1}H NMR (400 MHz, $CDCl_3$) δ 7.18 (t, *J* = 7.9 Hz, 2H), 7.01 (s, 1H), 6.72 (t, *J* = 7.3 Hz, 1H), 6.66 (d, *J* = 7.9 Hz, 2H), 4.69 (d, *J* = 7.4 Hz, 1H), 4.20 (q, *J* = 7.1 Hz, 2H), 4.02 (br s, 1H), 2.70-2.62 (m, 1H), 2.55-2.44 (m, 1H), 2.40-2.31(m, 1H), 2.03-1.97 (m, 1H), 1.26 (t, *J* = 7.1 Hz, 3H). ^{13}C NMR (100 MHz, $CDCl_3$) δ 164.79, 147.95, 147.46, 137.20, 129.27, 117.76, 113.96, 60.54, 58.82, 31.98, 31.32, 14.36.

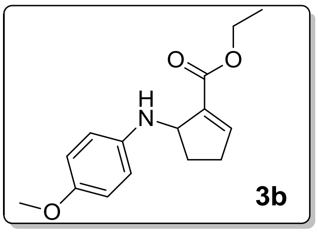

Ethyl 5-((4-methoxyphenyl)amino)cyclopent-1-enecarboxylate (3b): The general procedure was followed, and flash chromatography (*n*-hexane/EtOAc = 50/1) afforded 154 mg (59% yield). Yellow solid. ^{1}H NMR (400 MHz, $CDCl_3$) δ 6.98 (s, 1H), 6.81-6.76 (m, 2H), 6.67-6.62 (m, 2H), 4.60 (d, *J* = 7.5 Hz, 1H), 4.20 (q, *J* = 7.1 Hz, 2H), 3.86 (br s, 1H), 3.75 (s, 3H), 2.69-2.60 (m, 1H), 2.51- 2.47 (m, 1H), 2.39-2.27 (m, 1H), 2.01-1.94 (m, 1H), 1.26 (t, *J* = 7.1 Hz, 3H). ^{13}C NMR (100 MHz, $CDCl_3$) δ 164.92, 152.65, 147.19, 142.15, 137.30, 115.76, 114.92, 60.54, 59.93, 55.94, 31.88, 31.35, 14.39.

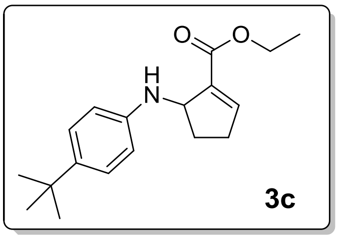

Ethyl 5-((4-(*tert*-butyl)phenyl)amino)cyclopent-1-enecarboxylate (3c): The general procedure was followed, and flash chromatography (*n*-hexane/EtOAc = 50/1) afforded 224 mg (78% yield). Yellow oil. ^{1}H NMR (400 MHz, $CDCl_3$) δ 7.22 (d, *J* = 8.6 Hz, 2H), 7.00 (s, 1H), 6.63 (d, *J* = 8.6 Hz, 2H), 4.66 (d, *J* = 7.4 Hz, 1H), 4.20 (q, *J* = 7.1 Hz, 2H), 3.83 (br s, 1H), 2.71-2.58 (m, 1H), 2.48 (m, 1H), 2.40-2.29 (m, 1H), 2.05 -1.96 (m, 1H), 1.29 (s, 9H), 1.25 (t, *J* = 7.1 Hz, 3H). ^{13}C NMR (100 MHz, $CDCl_3$) δ 164.85, 147.39, 145.59, 140.57, 137.28, 126.05, 113.76, 60.51, 59.02, 33.98, 32.03, 31.68, 31.34, 14.38.

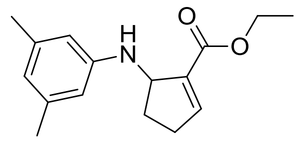

3d

Ethyl 5-((3,5-dimethylphenyl)amino)cyclopent-1-enecarboxylate (3d): The general procedure was followed, and flash chromatography (*n*-hexane/EtOAc = 50/1) afforded 236 mg (91% yield). Yellow solid. ^{1}H NMR (400 MHz, $CDCl_3$) δ 7.01 (s, 1H), 6.40 (s, 1H), 6.31 (s, 2H), 4.67 (d, *J* = 7.4 Hz, 1H), 4.21 (q, *J* = 7.1 Hz, 2H), 3.27 (br s, 1H), 2.72-2.58 (m, 1H), 2.56-2.42 (m, 1H), 2.42-2.28 (m, 1H), 2.25 (s, 6H), 2.05-1.92 (m, 1H), 1.27 (t, *J* = 7.1 Hz, 3H). ^{13}C NMR (100 MHz, $CDCl_3$) δ 164.80, 147.99, 147.42, 138.87, 137.26, 119.76, 111.88, 60.51, 58.73, 32.10, 31.30, 21.63, 14.36.

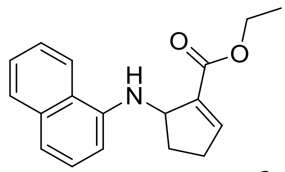

3e

Ethyl 5-(naphthalen-1-ylamino)cyclopent-1-enecarboxylate (3e): The general procedure was followed, and flash chromatography (*n*-hexane/EtOAc = 50/1) afforded 239 mg (85% yield). Yellow oil. ^{1}H NMR (400 MHz, $CDCl_3$) δ 7.91-7.80 (m, 2H), 7.52-7.37 (m, 3H), 7.32 (d, *J* = 8.2 Hz, 1H), 7.14 (s, 1H), 6.74 (d, *J* = 7.5 Hz, 1H), 5.01 (br s, 1H), 4.95-4.79 (m, 1H), 4.27 (dd, *J* = 7.1, 2.9 Hz, 2H), 2.83-2.63 (m, 1H), 2.64-2.41 (m, 2H), 2.11 (m, 1H), 1.30 (t, *J* = 7.1 Hz, 3H). ^{13}C NMR (100 MHz, $CDCl_3$) δ 164.92, 147.97, 143.17, 136.64, 134.43, 128.61, 126.48, 125.76, 124.82,

124.26, 120.35, 117.87, 106.00, 60.58, 59.02, 31.80, 31.47, 14.32.

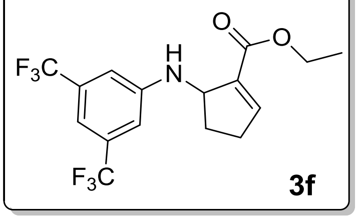

3f

Ethyl 5-((3,5-bis(trifluoromethyl)phenyl)amino)cyclopent-1-enecarboxylate (3f): The general procedure was followed, and flash chromatography (*n*-hexane/EtOAc = 50/1) afforded 132 mg (36% yield). Light yellow solid. ^{1}H NMR (400 MHz, $CDCl_3$) δ 7.15 (s, 1H), 7.06 (s, 1H), 6.98 (s, 2H), 4.75 (s, 1H), 4.44 (d, *J* = 5.9 Hz, 1H), 4.20 (q, *J* = 7.1 Hz, 2H), 2.73-2.64 (m, 1H), 2.60-2.50 (m, 1H), 2.46-2.42 (m, 1H), 1.96-1.90 (m, 1H), 1.24 (t, *J* = 7.1 Hz, 3H). ^{13}C NMR (100 MHz, $CDCl_3$) δ 164.31, 148.24, 147.96, 136.20, 132.48, 132.15, 131.83, 124.95, 122.24, 112.60 (d, *J* = 3.6 Hz), 110.32 (dt, *J* = 7.9, 4.1 Hz), 60.66, 58.34, 31.65, 31.16, 14.15.

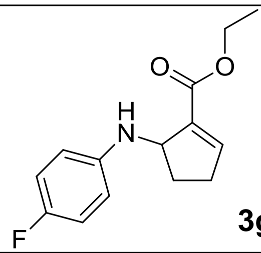

3g

Ethyl 5-((4-fluorophenyl)amino)cyclopent-1-enecarboxylate (3g): The general procedure was followed, and flash chromatography (*n*-hexane/EtOAc = 50/1) afforded 167 mg (67% yield). Yellow solid. ^{1}H NMR (400 MHz, $CDCl_3$) δ 7.00 (t, *J* = 3.0 Hz, 1H), 6.88 (t, *J* = 8.8 Hz, 2H), 6.59 (dd, *J* = 9.0, 4.5 Hz, 2H), 4.61 (d, *J* = 7.5 Hz, 1H), 4.20 (q, *J* = 7.1 Hz, 2H), 3.94 (br s, 1H), 2.66 (m, 1H), 2.49 (m, 1H), 2.34 (m, 1H), 1.95 (m, 1H), 1.26 (t, *J* = 7.1 Hz, 3H). ^{13}C NMR (100 MHz, $CDCl_3$) δ 164.81, 157.39, 155.05, 147.39, 144.30 (d, *J* = 1.9 Hz), 137.05, 115.67 (d, *J* = 22.3 Hz), 114.97 (d, *J* = 7.4 Hz), 60.59, 59.58, 31.84, 31.33, 14.36.

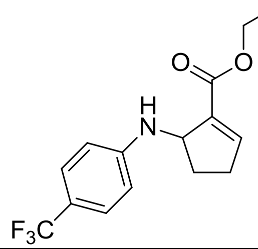

3h

Ethyl 5-((4-(trifluoromethyl)phenyl)amino)cyclopent-1-enecarboxylate (3h): The general procedure was followed, and flash chromatography (*n*-hexane/EtOAc = 50/1) afforded 72 mg (24% yield). Yellow oil. ^{1}H NMR (400 MHz, $CDCl_3$) δ 7.40 (d, *J* = 8.6 Hz, 2H), 7.03 (s, 1H), 6.64 (d, *J* = 8.6 Hz, 2H), 4.73 (d, *J* = 4.0 Hz 1H), 4.30 (br s, 1H), 4.19 (q, *J* = 7.1 Hz, 2H), 2.73-2.59 (m, 1H), 2.52 (m, 1H), 2.46-2.30 (m, 1H), 2.03-1.87 (m, 1H), 1.24 (t, *J* = 7.1 Hz, 3H). ^{13}C NMR (100 MHz, $CDCl_3$) δ 164.42, 150.16, 147.75, 136.59, 126.51 (q, *J* = 3.8 Hz), 123.67, 118.93 (d, *J* = 32.6 Hz), 112.63, 60.55, 58.19, 31.74, 31.17, 14.20.

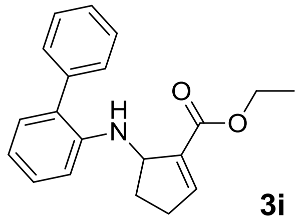

3i

Ethyl 5-([1,1'-biphenyl]-2-ylamino)cyclopent-1-enecarboxylate (3i): The general procedure was followed, and flash chromatography (*n*-hexane/EtOAc = 50/1) afforded 61 mg (20% yield). Yellow solid. ^{1}H NMR (400 MHz, $CDCl_3$) δ 7.44-7.37 (m, 4H), 7.36-7.29 (m, 1H), 7.29-7.23 (m, 1H), 7.10 (dd, *J* = 7.4, 1.5 Hz, 1H), 6.95 (s, 1H), 6.80 (dd, *J* = 13.9, 7.6 Hz, 2H), 4.71 (d, *J* = 5.7 Hz, 1H), 4.20 (br s, 1H), 4.15 (q, *J* = 7.1 Hz, 2H), 2.58-2.52 (m, 1H), 2.49-2.41 (m, 1H), 2.39-2.33 (m, 1H), 2.00-1.94 m, 1H), 1.20 (t, *J* = 7.1 Hz, 3H). ^{13}C NMR (100 MHz, $CDCl_3$) δ 164.56, 147.72, 144.77, 139.72, 137.14, 130.49, 129.46, 128.90, 128.67, 128.18, 127.19, 117.18, 111.60, 60.46, 58.80, 32.16, 31.25, 14.32.

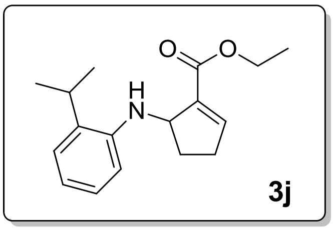

Ethyl 5-((2-isopropylphenyl)amino)cyclopent-1-enecarboxylate (3j): The general procedure was followed, and flash chromatography (*n*-hexane/EtOAc = 50/1) afforded 101 mg (37% yield). Yellow oil. ^{1}H NMR (400 MHz, $CDCl_3$) δ 7.15 (dd, *J* = 15.5, 7.7 Hz, 2H), 7.07 (s, 1H), 6.77 (t, *J* = 7.5 Hz, 1H), 6.73 (d, *J* = 8.0 Hz, 1H), 4.70 (d, *J* = 6.9 Hz, 1H), 4.22 (q, *J* = 7.1 Hz, 2H), 4.15 (s, 1H), 2.85 (m, 1H), 2.73-2.61 (m, 1H), 2.51 (m, 1H), 2.45-2.34 (m, 1H), 2.02 (m, 1H), 1.30-1.21 (m, 9H). ^{13}C NMR (100 MHz, $CDCl_3$) δ 164.93, 147.96, 144.68, 137.10, 133.23, 126.63, 125.04, 117.69, 111.77, 60.58, 58.87, 32.17, 31.39, 27.22, 22.64, 22.27, 14.39.

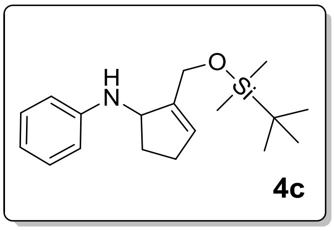

***N*-(2-(((*tert*-Butyldimethylsilyl)oxy)methyl)cyclopent-2-en-1-yl)aniline (4c):** The general procedure was followed, and flash chromatography (*n*-hexane/EtOAc = 50/1) afforded 30 mg (10% yield). Light yellow oil. ^{1}H NMR (400 MHz, $CDCl_3$) δ 7.16 (dd, *J* = 8.5, 7.4 Hz, 2H), 6.68 (t, *J* = 7.3 Hz, 1H), 6.61 (d, *J* = 7.6 Hz, 2H), 5.83 (d, *J* = 1.2 Hz, 1H), 4.44 (d, *J* = 5.6 Hz, 1H), 4.26 (d, *J* = 13.9 Hz, 2H), 4.04 (br s, 1H), 2.49-2.41 (m, 1H), 2.40-2.26 (m, 2H), 1.83-1.77(m, 1H), 0.90 (s, 9H), 0.06 (d, *J* = 2.0 Hz, 6H). ^{13}C NMR (101 MHz, $CDCl_3$) δ 148.11, 144.09, 129.71, 129.34, 117.12, 113.33, 61.33, 60.04, 32.43, 30.24, 29.85, 26.09, -5.22.

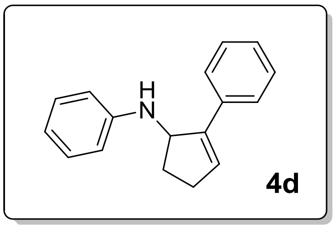

***N*-(2-phenylcyclopent-2-en-1-yl)aniline (4d)[14]:** The general procedure was

followed, and flash chromatography (*n*-hexane/EtOAc = 50/1) afforded 151 mg (65% yield). Light yellow oil. ^{1}H NMR (400 MHz, $CDCl_3$) δ 7.49 (d, *J* = 7.3 Hz, 2H), 7.29 (t, *J* = 7.4 Hz, 2H), 7.24 -7.15 (m, 3H), 6.70 (t, *J* = 7.3 Hz, 1H), 6.62 (d, *J* = 7.7 Hz, 2H), 6.40 (s, 1H), 4.88 (d, *J* = 7.1 Hz, 1H), 3.75 (br s, 1H), 2.69-2.60 (m, 1H), 2.54-2.46 (m, 1H), 2.37-2.30 (m, 1H), 2.05-1.99 (m, 1H). ^{13}C NMR (100 MHz, $CDCl_3$) δ 147.75, 142.87, 130.23, 129.45, 128.69, 127.50, 126.33, 117.14, 113.17, 59.15, 31.73, 31.13.

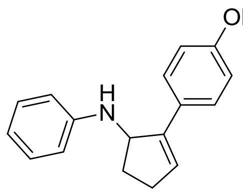

4e

***N*-(2-(4-Methoxyphenyl)cyclopent-2-en-1-yl)aniline (4e):** The general procedure was followed, and flash chromatography (*n*-hexane/EtOAc = 50/1) afforded 170 mg (64% yield). Light yellow oil. ^{1}H NMR (400 MHz, $CDCl_3$) δ 7.48-7.40 (m, 2H), 7.24-7.17 (m, 2H), 6.88-6.81 (m, 2H), 6.71 (t, *J* = 7.3 Hz, 1H), 6.64 (d, *J* = 7.7 Hz, 2H), 6.27 (d, *J* = 2.2 Hz, 1H), 4.85 (d, *J* = 7.1 Hz, 1H), 3.80 (s, 3H), 3.76 (br s, 1H), 2.68-2.60 (m, 1H), 2.53-2.50 (m, 1H), 2.39-2.30(m, 1H), 2.06-1.99 (m, 1H). ^{13}C NMR (100 MHz, $CDCl_3$) δ 159.09, 147.82, 142.30, 129.43, 127.95, 127.53, 117.08, 114.07, 113.16, 59.28, 55.41, 31.73, 31.06.

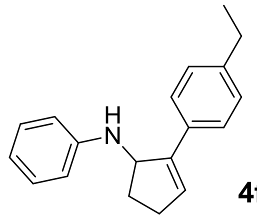

4f

***N*-(2-(4-Ethylphenyl)cyclopent-2-en-1-yl)aniline (4f)[13]:** The general procedure was followed, and flash chromatography (*n*-hexane/EtOAc = 50/1) afforded 171 mg (65% yield). Light yellow solid. ^{1}H NMR (400 MHz, $CDCl_3$) δ 7.43 (d, *J* = 8.2 Hz, 2H), 7.26-7.18 (m, 2H), 7.15 (d, *J* = 8.2 Hz, 2H), 6.72 (t, *J* = 7.3 Hz, 1H), 6.65 (d, *J* = 7.6 Hz, 2H), 6.37 (m, 1H), 4.88 (d, *J* = 6.8 Hz, 1H), 3.79 (br s, 1H), 2.64 (q, *J* = 7.6 Hz, 3H), 2.55-2.47 (m, 1H), 2.40-2.31 (m, 1H), 2.07-2.00 (m, 1H), 1.24 (t, *J* = 7.6 Hz, 3H). ^{13}C NMR (100 MHz, $CDCl_3$) δ 147.82, 143.70, 142.73, 132.14, 129.42, 129.22,

128.19, 126.31, 117.06, 113.15, 59.18, 31.73, 31.09, 28.71, 15.67.

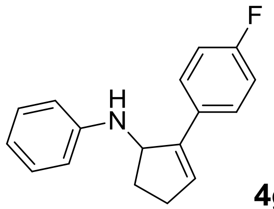

4g

***N*-(2-(4-Fluorophenyl)cyclopent-2-en-1-yl)aniline:** The general procedure was followed, and flash chromatography (*n*-hexane/EtOAc = 50/1) afforded 162 mg (64% yield). Light yellow solid. ^{1}H NMR (400 MHz, $CDCl_3$) δ 7.45 (m, 2H), 7.25-7.14 (m, 2H), 6.96 (m, 2H), 6.71 (t, *J* = 7.3 Hz, 1H), 6.62 (d, *J* = 7.7 Hz, 2H), 6.30 (s, 1H), 4.83 (d, *J* = 7.2 Hz, 1H), 3.69 (br s, 1H), 2.66-2.58 (m, 1H), 2.52-2.45 (m, 1H), 2.38-2.29 (m, 1H), 2.03-1.97 (m, 1H). ^{13}C NMR (100 MHz, $CDCl_3$) δ 163.46, 161.00, 147.59, 141.90, 130.95 (d, *J* = 3.4 Hz), 129.76 (d, *J* = 1.9 Hz), 129.46, 127.93 (d, *J* = 7.9 Hz), 117.28, 115.60, 115.39, 113.20, 59.28, 31.68, 31.05.

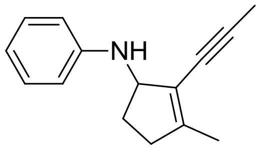

4h

***N*-(3-Methyl-2-(prop-1-yn-1-yl)cyclopent-2-en-1-yl)aniline (4h):** The general procedure was followed, and flash chromatography (*n*-hexane/EtOAc = 50/1) afforded 65 mg (31% yield). Yellow oil. ^{1}H NMR (400 MHz, $CDCl_3$) δ 7.21-7.14 (m, 2H), 6.70 (t, *J* = 7.3 Hz, 1H), 6.65 (d, *J* = 7.7 Hz, 2H), 4.42 (d, *J* = 5.4 Hz, 1H), 3.92 (br s, 1H), 2.55-2.42 (m, 1H), 2.40-2.28 (m, 2H), 1.98 (s, 3H), 1.89 (s, 3H), 1.79-1.69 (m, 1H). ^{13}C NMR (100 MHz, $CDCl_3$) δ 149.06, 148.11, 129.26, 120.45, 117.29, 113.54, 90.67, 75.01, 62.48, 35.83, 31.49, 16.20, 4.69.

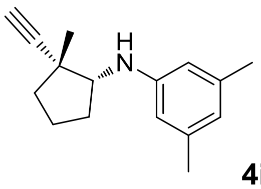

4i

***N*-((1*R*,2*R*)-2-Ethynyl-2-methylcyclopentyl)-3,5-dimethylaniline (4i)**[14]**:** The general procedure was followed, and flash chromatography (*n*-hexane/EtOAc = 50/1) afforded 102 mg (45% yield). Light yellow oil. ^{1}H NMR (400 MHz, $CDCl_3$) δ 6.39 (s, 2H), 6.36 (s, 1H), 3.95 (t, *J* = 7.6 Hz, 1H), 3.50 (br s, 1H), 2.38-2.26 (m, 1H), 2.23 (s, 6H), 2.18 (s, 1H), 2.11-1.99 (m, 1H), 1.83-1.66 (m, 3H), 1.47-1.35 (m, 1H), 1.22 (s, 3H). ^{13}C NMR (100 MHz, $CDCl_3$) δ 147.96, 138.82, 119.21, 111.27, 92.20, 68.45, 63.12, 40.37, 40.13, 32.50, 21.49 (d, *J* = 4.2 Hz), 20.96.

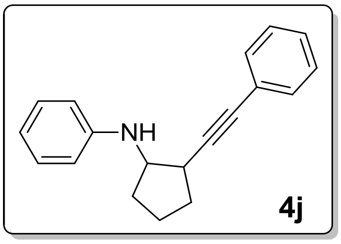

The general procedure was followed, and flash chromatography (*n*-hexane/EtOAc = 50/1) afforded two diastereoisomers. Data for **4j-*cis***: Colorless oil (35mg, 13%). ^{1}H NMR (400 MHz, $CDCl_3$) δ 7.40-7.32 (m, 2H), 7.31-7.25 (m, 3H), 7.17 (dd, *J* = 11.5, 4.3 Hz, 2H), 6.72-6.63 (m, 3H), 4.22 (s, 1H), 3.88 (q, *J* = 6.4 Hz, 1H), 3.25 (q, *J* = 6.4 Hz, 1H), 2.16-1.85 (m, 4H), 1.79-1.61 (m, 2H). ^{13}C NMR (101 MHz, $CDCl_3$) δ 148.05, 131.75, 129.35, 128.38, 127.98, 123.60, 117.50, 113.79, 89.70, 84.47, 57.11, 35.74, 31.57 (d, *J* = 4.5 Hz), 22.05. Data for **4j-*trans***: Light yellow oil (72 mg, 28% yield). ^{1}H NMR (400 MHz, $CDCl_3$) δ 7.39 (dd, *J* = 6.5, 3.2 Hz, 2H), 7.28 (dd, *J* = 5.0, 2.0 Hz, 3H), 7.23-7.15 (m, 2H), 6.76-6.67 (m, 3H), 3.92 (dd, *J* = 12.3, 5.3 Hz, 1H), 3.78 (br s, 1H), 2.91-2.80 (m, 1H), 2.40-2.27 (m, 1H), 2.16-2.05 (m, 1H), 1.86 (m, 3H), 1.54-1.46 (m, 1H). ^{13}C NMR (100 MHz, $CDCl_3$) δ 147.57, 131.61, 129.27, 128.20, 127.67, 123.77, 117.47, 113.48, 92.40, 81.90, 61.52, 38.23, 33.23, 31.94, 23.24.

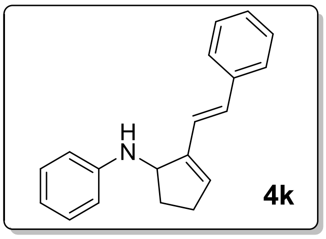

(*E*)-*N*-(2-Styrylcyclopent-2-en-1-yl)aniline: The general procedure was followed, and flash chromatography (*n*-hexane/EtOAc = 50/1) afforded 115 mg (44% yield). Yellow oil. ^{1}H NMR (400 MHz, $CDCl_3$) δ 7.37 (d, *J* = 7.4 Hz, 2H), 7.29 (t, *J* = 7.6 Hz,

2H), 7.21 (m, 3H), 6.92 (d, J = 16.3 Hz, 1H), 6.72 (t, J = 7.3 Hz, 1H), 6.66 (d, J = 7.6 Hz, 2H), 6.60 (d, J = 16.3 Hz, 1H), 6.07 (s, 1H), 4.73 (d, J = 7.1 Hz, 1H), 3.80 (br s, 1H), 2.65-2.57 (m, 1H), 2.48-2.41 (m, 1H), 2.34-2.24 (m, 1H), 2.04-1.98 (m, 1H). ^{13}C NMR (100 MHz, $CDCl_3$) δ 147.92, 142.90, 137.53, 135.05, 129.86, 129.44, 128.72, 127.58, 126.50, 123.50, 117.12, 113.24, 58.27, 31.83, 31.02.

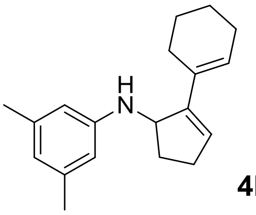

4l

***N*-(2-(Cyclohex-1-en-1-yl)cyclopent-2-en-1-yl)-3,5-dimethylaniline**[14]**:** The general procedure was followed, and flash chromatography (*n*-hexane/EtOAc = 50/1) afforded 107 mg (40% yield). Light yellow oil. ^{1}H NMR (400 MHz, $CDCl_3$) δ 6.36 (s, 1H), 6.26 (s, 2H), 5.83 (s, 2H), 4.56 (d, J = 7.0 Hz, 1H), 3.66 (br s, 1H), 2.60-2.47 (m, 1H), 2.39-2.32 (m, 1H), 2.25 (s, 6H), 2.17-2.04 (m, 3H), 2.00-1.94 (m, 1H), 1.76-1.64 (m, 3H), 1.64-1.51 (m, 3H). ^{13}C NMR (100 MHz, $CDCl_3$) δ 148.10, 145.08, 139.03, 131.40, 127.46, 125.88, 118.91, 110.89, 58.31, 31.70, 30.85, 26.38, 25.82, 22.87, 22.45, 21.70.

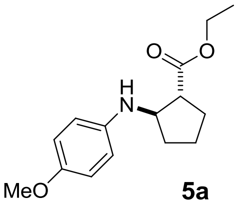

5a

***trans*-Ethyl 2-((4-methoxyphenyl)amino)cyclopentanecarboxylate (5a)**[13]**:** colorless oil (53 mg, 40%), Silica gel column chromatography (10:1 hexane/EtOAc). ^{1}H NMR (400 MHz, $CDCl_3$) δ 6.76 (d, J = 9.0 Hz, 2H), 6.60 (d, J = 8.9 Hz, 2H), 4.16-4.06 (m, 2H), 4.01 (dd, J = 13.0, 6.1 Hz, 1H), 3.74 (s, 3H), 3.19 (br s, 1H), 2.68-2.57 (m, 1H), 2.22-2.13 (m, 1H), 2.04-1.89 (m, 2H), 1.82-1.73 (m, 2H), 1.55-1.47 (m, 1H), 1.22 (t, J = 7.1 Hz, 3H).

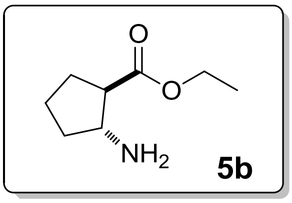

***trans*-Ethyl 2-aminocyclopentanecarboxylate (5b):** yellow-brown oil (24 mg, 80%). ^{1}H NMR (400 MHz, $CDCl_3$) δ 4.15 (q, *J* = 7.1 Hz, 2H), 3.43 (dd, *J* = 15.7, 8.1 Hz, 1H), 2.40 (dd, *J* = 17.2, 8.7 Hz, 1H), 2.04-1.98 (m, 1H), 1.89-1.81 (m, 3H), 1.73-1.65 (m, 1H), 1.45-1.35 (m, 1H), 1.26 (t, *J* = 7.2 Hz, 3H). ^{13}C NMR (100 MHz, $CDCl_3$) δ 175.53, 60.58, 57.29, 53.78, 35.37, 28.31, 22.76, 14.44.

3. Synthesis of *trans*-ethyl 2-aminocyclopentanecarboxylate

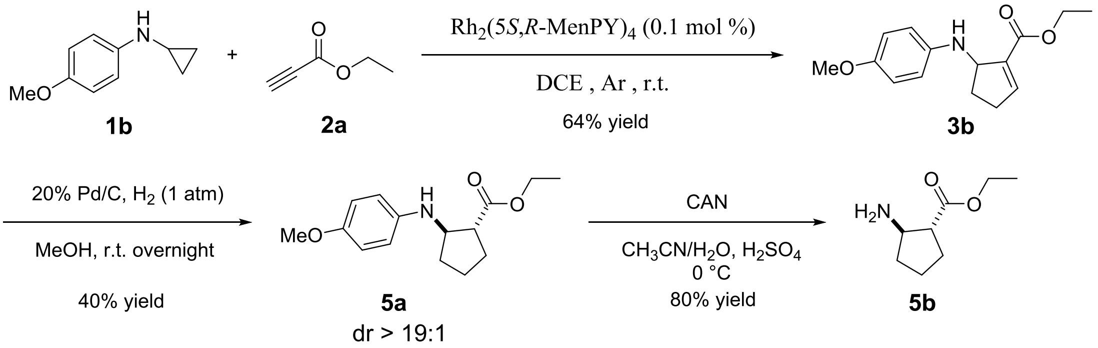

General procedure was performed to provide the [3 + 2] product **3b**. For the hydrogenation: To a clean dried 2-neck round bottom flask equipped with a stirring bar was added **3b** (131 mg, 0.5 mmol). After stirring in anhydrous MeOH (2 mL) for 5 min. Pd(C) (20 mg) was added carefully under N_2 atmosphere. A balloon filled with H_2 was equipped to the flask and the stirring was continued for 12 h at room temperature. After completion, celite was added to the reaction and stirred for additional 5 min prior to filtering through a pad of celite and washing with MeOH. The solution was concentrated in vacuum and purified by silica gel flash chromatography (10:1 hexane/EtOAc) to afford **5a** (53 mg, 40%).

Deprotection of PMP: To a pre-cooled solution of *trans*-*p*-methoxyphenyl amine **5a** (50 mg, 0.19 mmol) in 2 mL CH_3CN/H_2O (v/v = 3:1) was slowly added concentrated H_2SO_4 (26 μL, 0.38 mmol) at 0 °C. Ceric ammonium nitrate (208 mg, 0.38 mmol) was then added in one portion, and the mixture was stirred for one hour at 0 °C. The resulting mixture was then diluted with water (2 mL) and dichloromethane (2 mL). The aqueous phase was separated and basified to pH 10–11 using Na_2CO_3

solution. The basic aqueous layer was then extracted with ethyl acetate (3 × 5 mL). The combined organic layers were dried over Na_2SO_4 and concentrated to give the product **5b** (24 mg, 80%) as a colorless oil.

4. References

[1] Wang, Y. H.; Wolf, J.; Zavalij, P.; Doyle, M. P. *Angew. Chem. Int. Ed.* **2008**, *47*, 1439-1442.

[2] J. Waser, J. C. González-Gómez, H. Nambu, P. Huber, E. M. Carreira, *Org. Lett.*, 2005, *7*, 4249.

[3] Kazuhiro, M.; Toyohiko, A.; Takayuki, S. *Synlett*, **1994**, 107.

[4] DeAngelis, A. J.; Gildner, P. G.; Chow, R. T.; Colacot, J. *J. Org. Chem.* **2015**, *80*, 6794-6813.

[5] Gildner, P. G.; DeAngelis, A. J.; Colacot, T. J. *Org. Lett.* **2016**, *18*, 1442-1445.

[6] Gillaspy, M.; Lefker, B. A.; Hada, W. A.; Hoover, D. J. *Tetrahedron Lett*. **1995**, *36*, 7399-7402.

[7] Gildner, P. G.; DeAngelis, A. J.; Colacot, T. J. *Org. Lett.* **2016**, *18*, 1442-1445.

[8] Loeppky, R. N.; Elomari, S. *J. Org. Chem*. **2000**, *65*, 96-103.

[9] Cui, W.; Loeppky, R. N. *Tetrahedron* **2001**, *57*, 2953.

[10] Junya, S.; Hideyuki, S. Nitrogen-containing heterocyclic compound and use of same, EP2336105.

[11] Maity, S.; Zhu, M.; Shinabery, R. S.; Zheng, N. *Angew. Chem. Int. Ed*. **2012**, *51*, 222-226.

[12] Nguyen, T. H.; Maity, S.; Zheng, N. *Beilstein. J. Org. Chem,* **2014**, *10*, 975.

[13] Kuang, Y.; Ning, Y. B.; Zhu, J.; Wang, Y. H. *Org. Lett.* **2018**, *20*, 2693-2697.

[14] Nguyen, T. H.; Morris, S. A.; Zheng, N. *Adv. Synth. Catal*. **2014**, *356*, 2831-2837.

5. ^{1}H and ^{13}C NMR spectrum of compounds

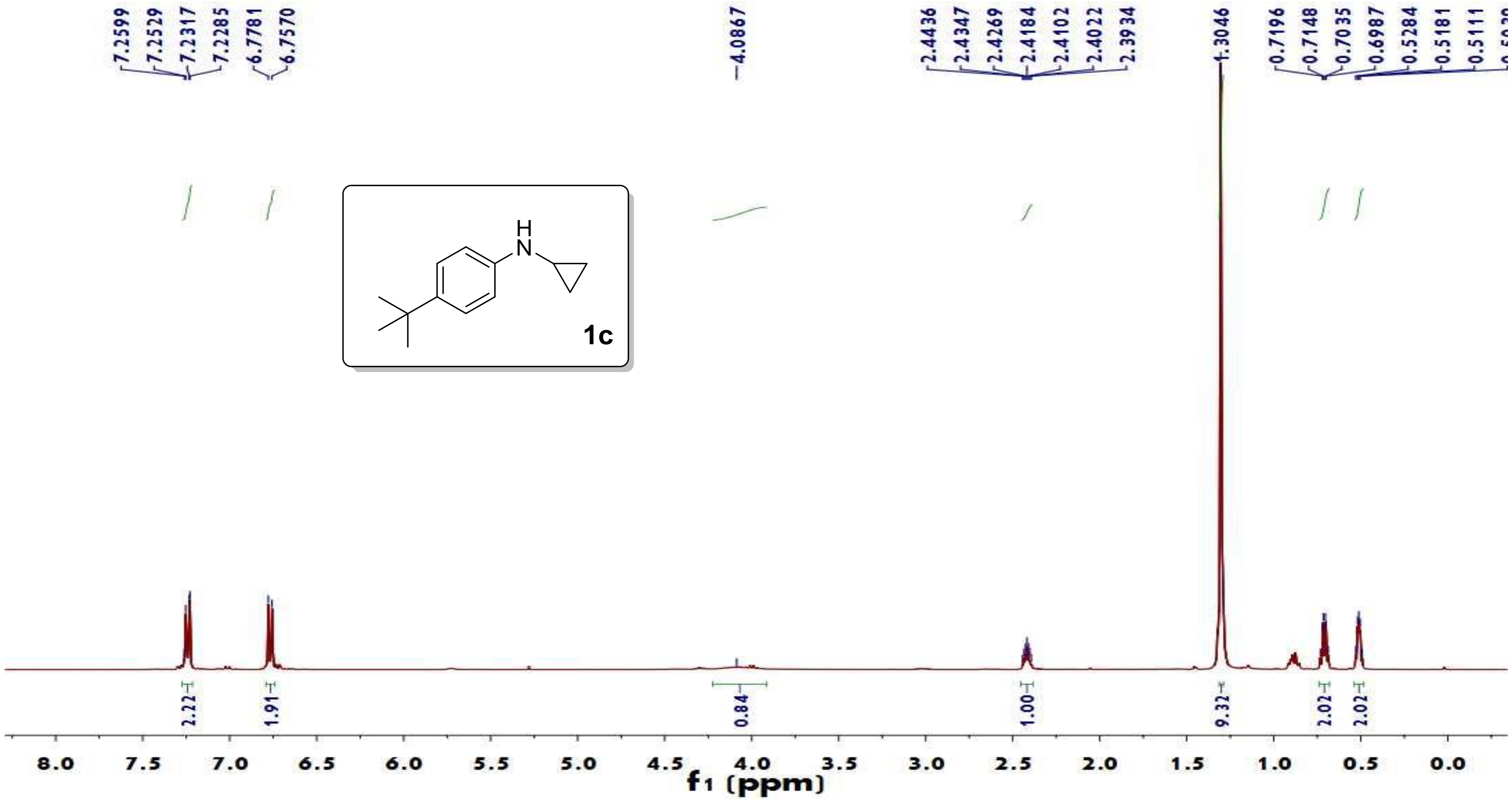

^{1}H NMR spectrum of 4-(*tert*-butyl)-*N*-cyclopropylaniline (400 MHz, $CDCl_3$).

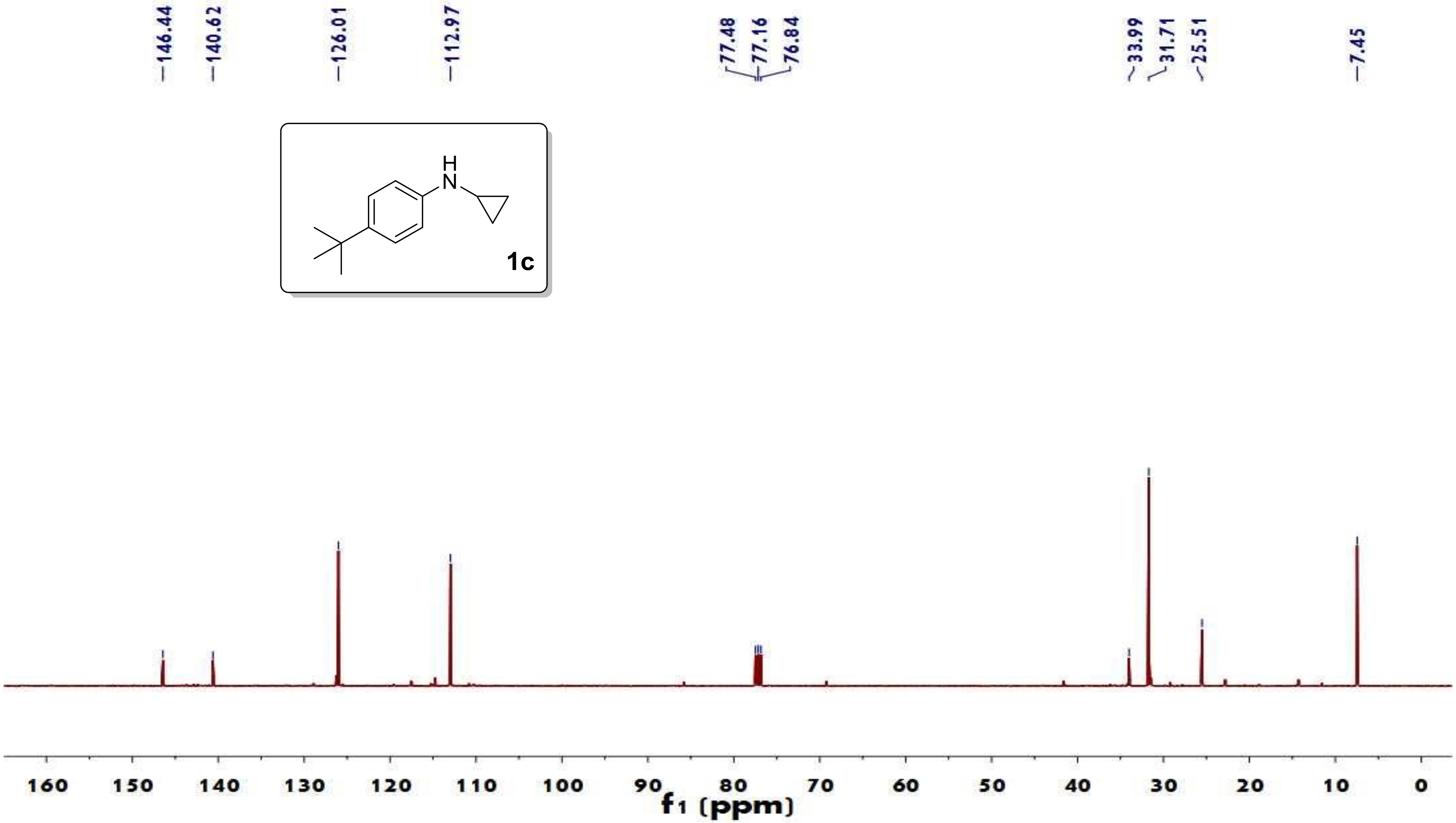

^{13}C NMR spectrum of 4-(*tert*-butyl)-*N*-cyclopropylaniline (400 MHz, $CDCl_3$).

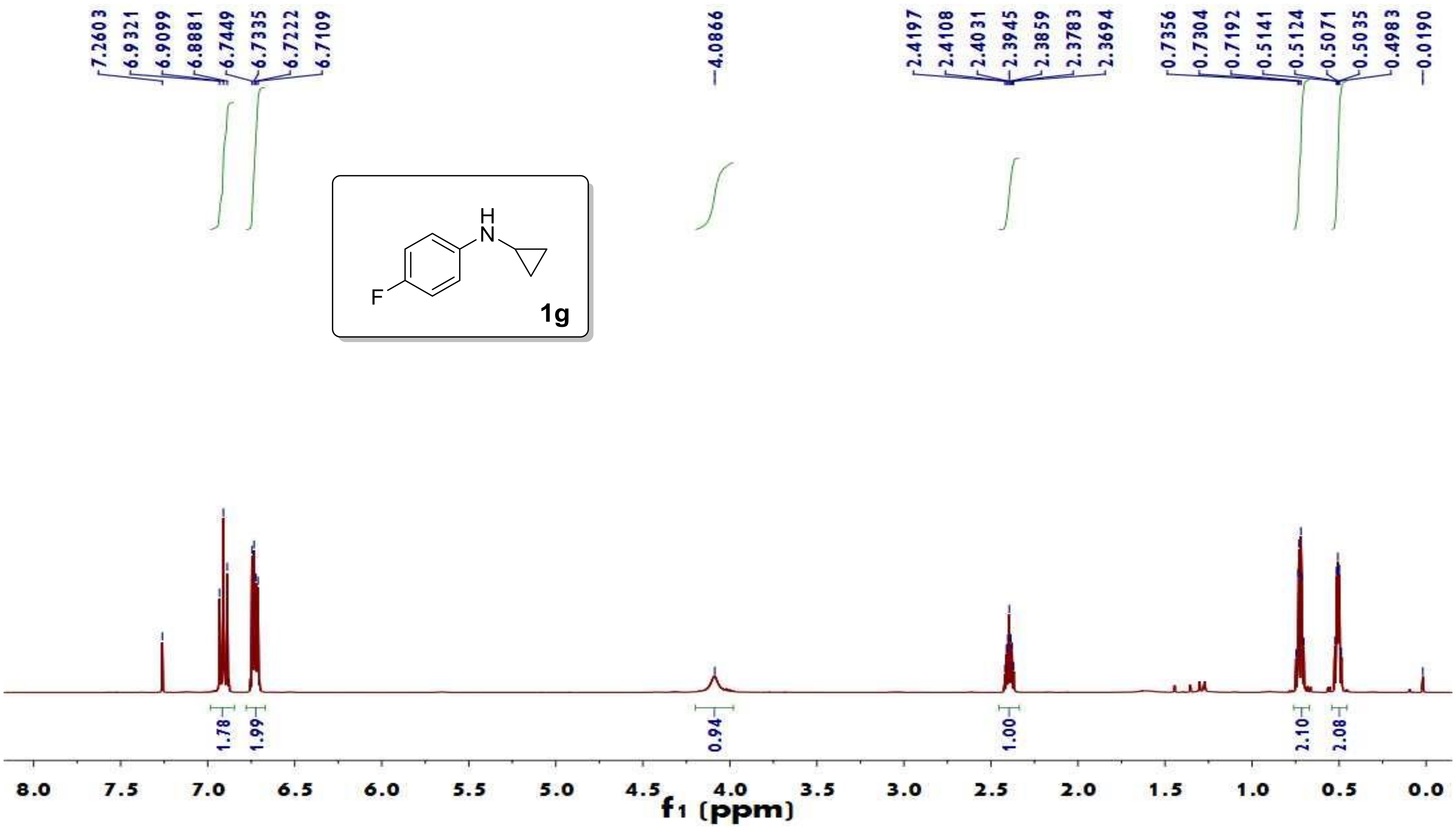

^{1}H NMR spectrum of *N*-cyclopropyl-4-fluoroaniline (400 MHz, $CDCl_3$).

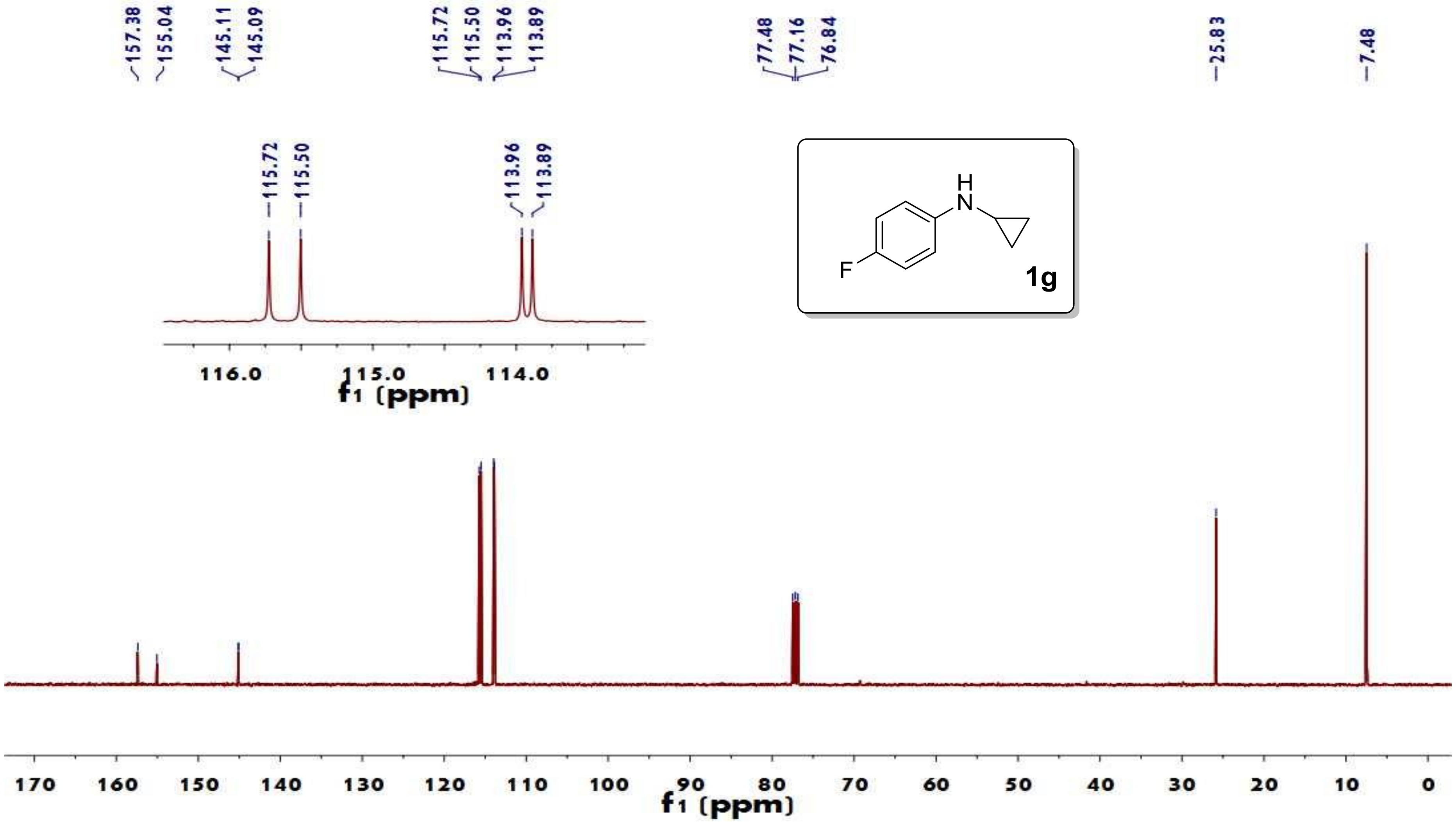

^{13}C NMR spectrum of *N*-cyclopropyl-4-fluoroaniline (400 MHz, $CDCl_3$).

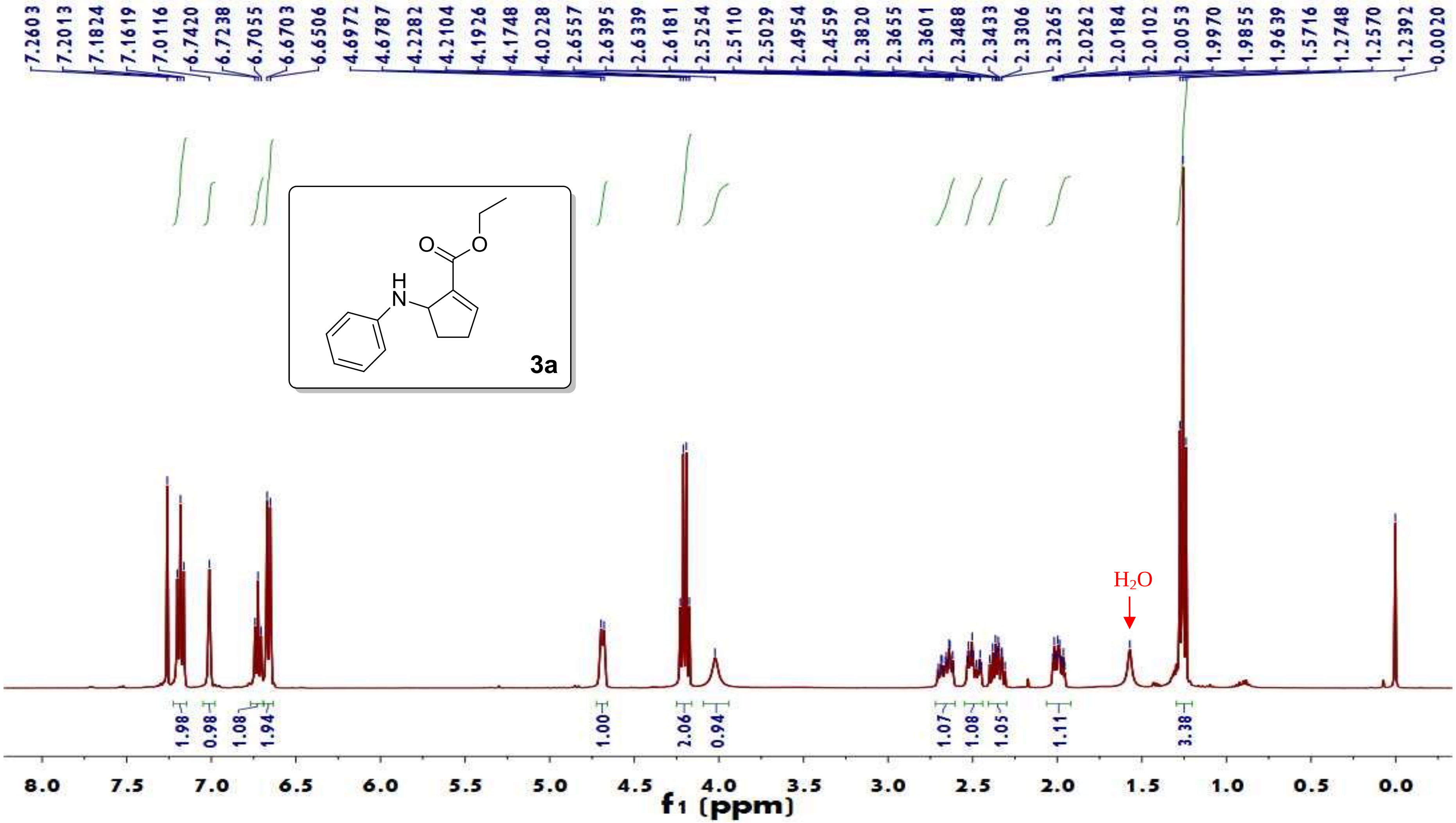

^{1}H NMR spectrum of 3a (400 MHz, $CDCl_3$).

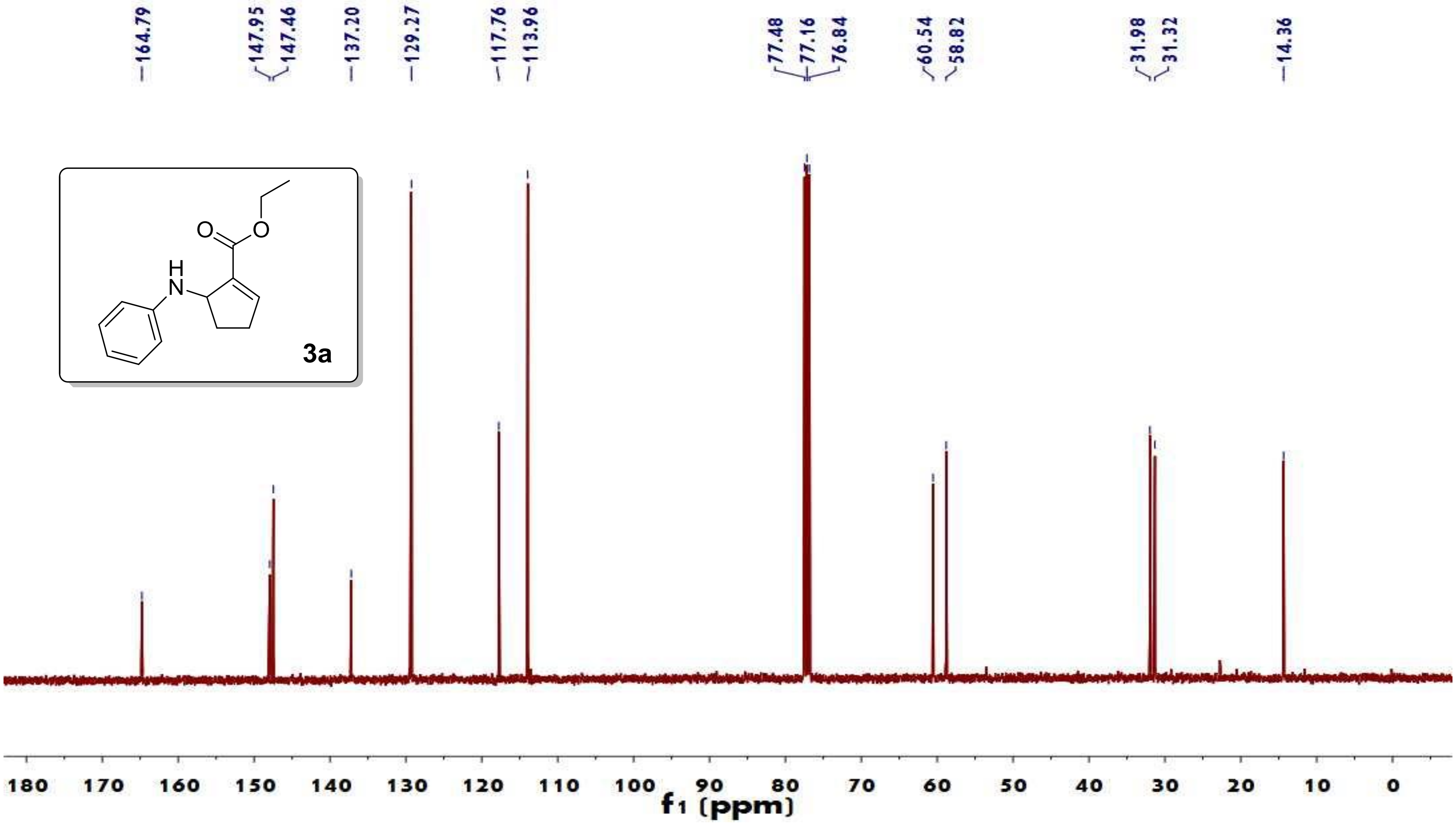

^{13}C NMR spectrum of 3a (400 MHz, $CDCl_3$).

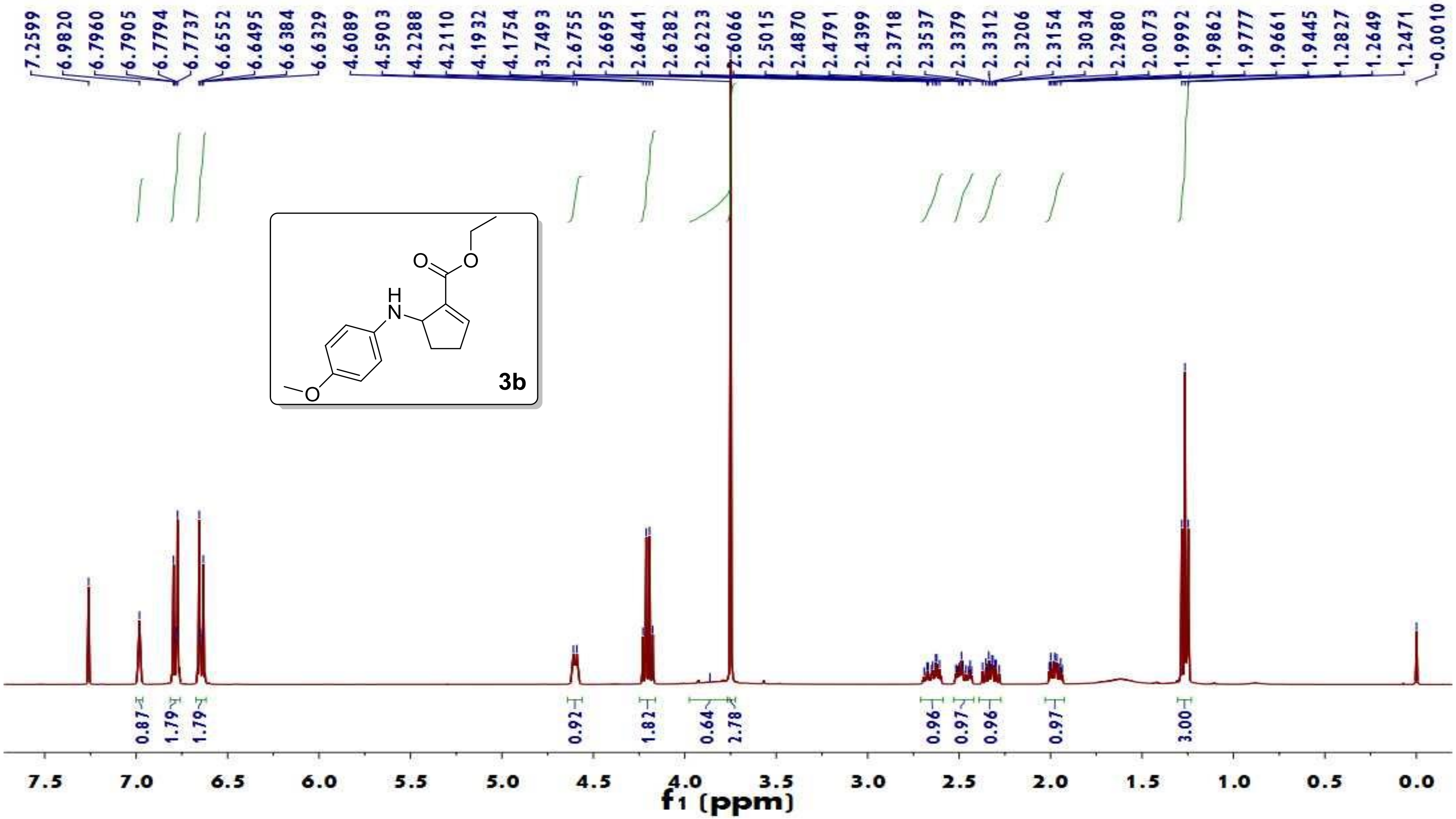

^{1}H NMR spectrum of 3b (400 MHz, $CDCl_3$).

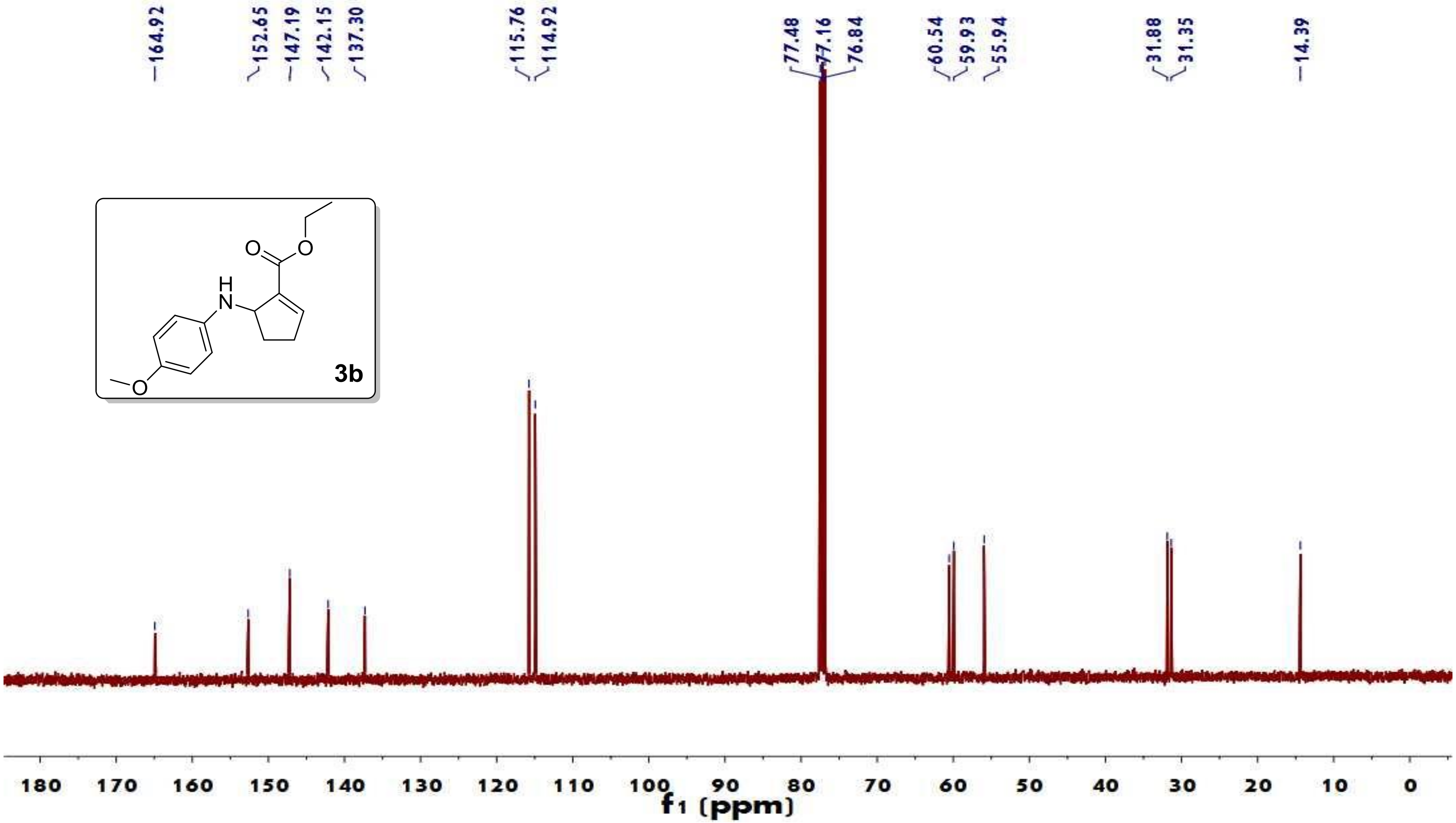

^{13}C NMR spectrum of 3b (400 MHz, $CDCl_3$).

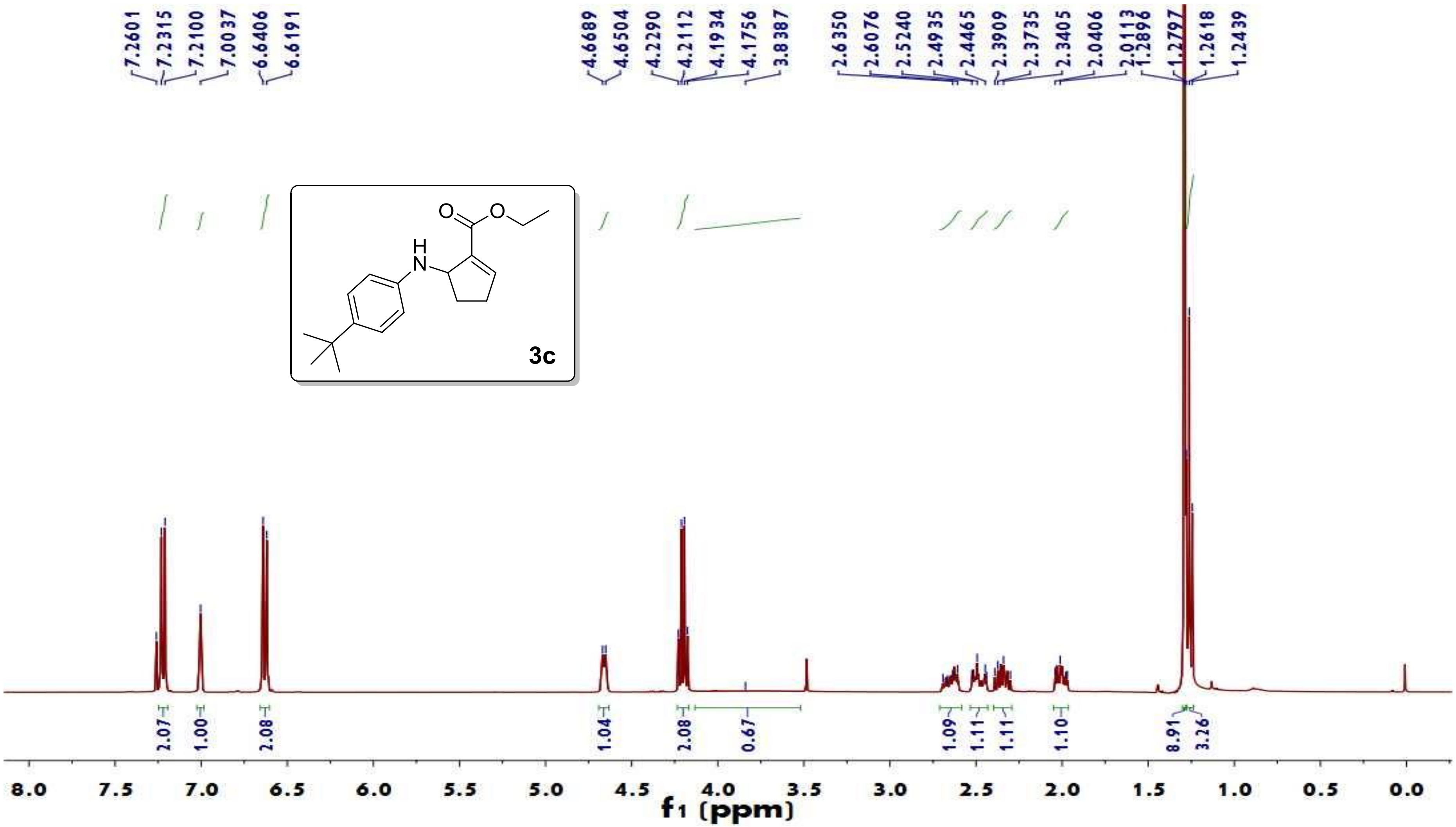

^{1}H NMR spectrum of 3c (400 MHz, $CDCl_3$).

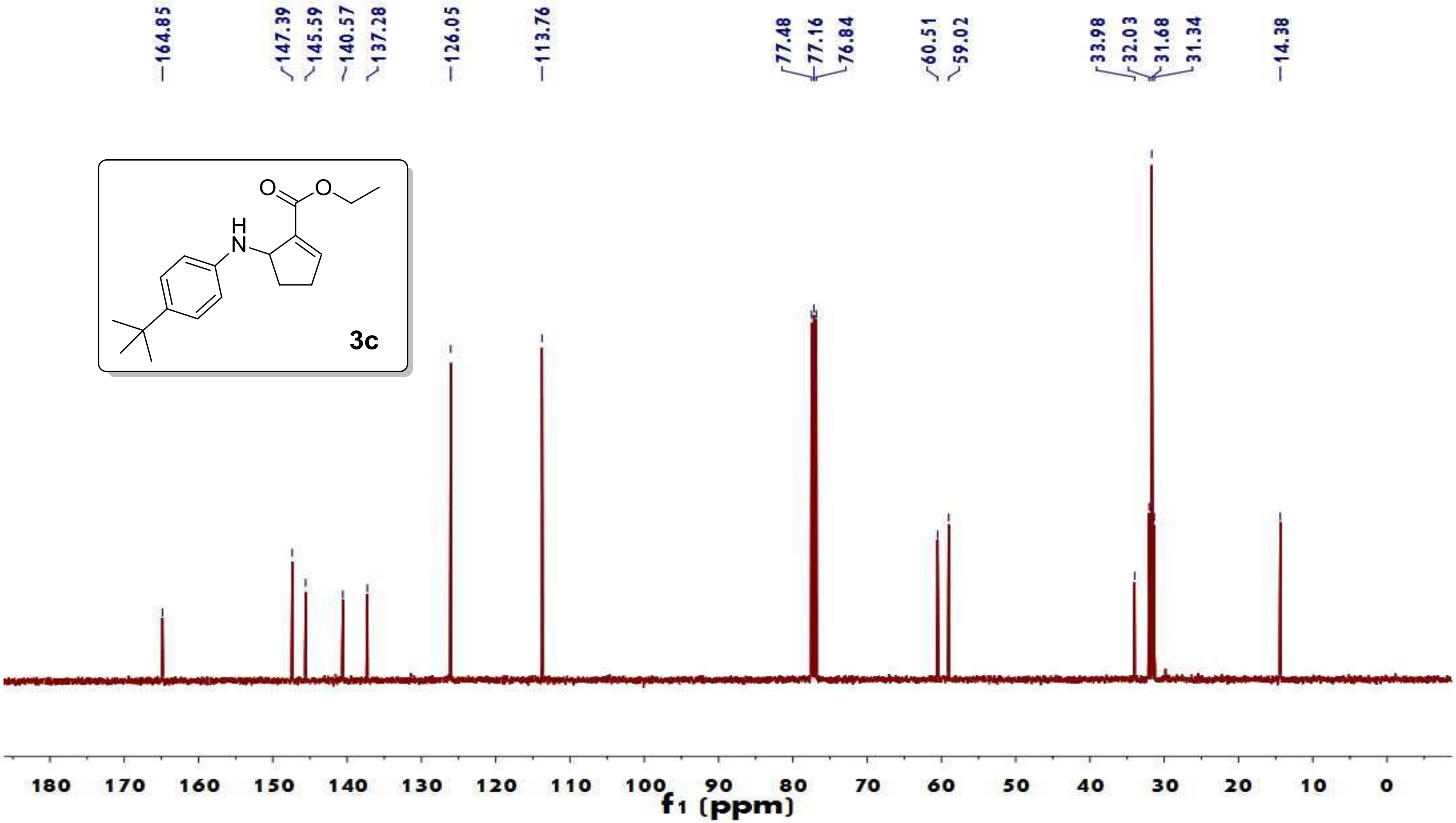

^{13}C NMR spectrum of 3c (400 MHz, CDCl$_3$).

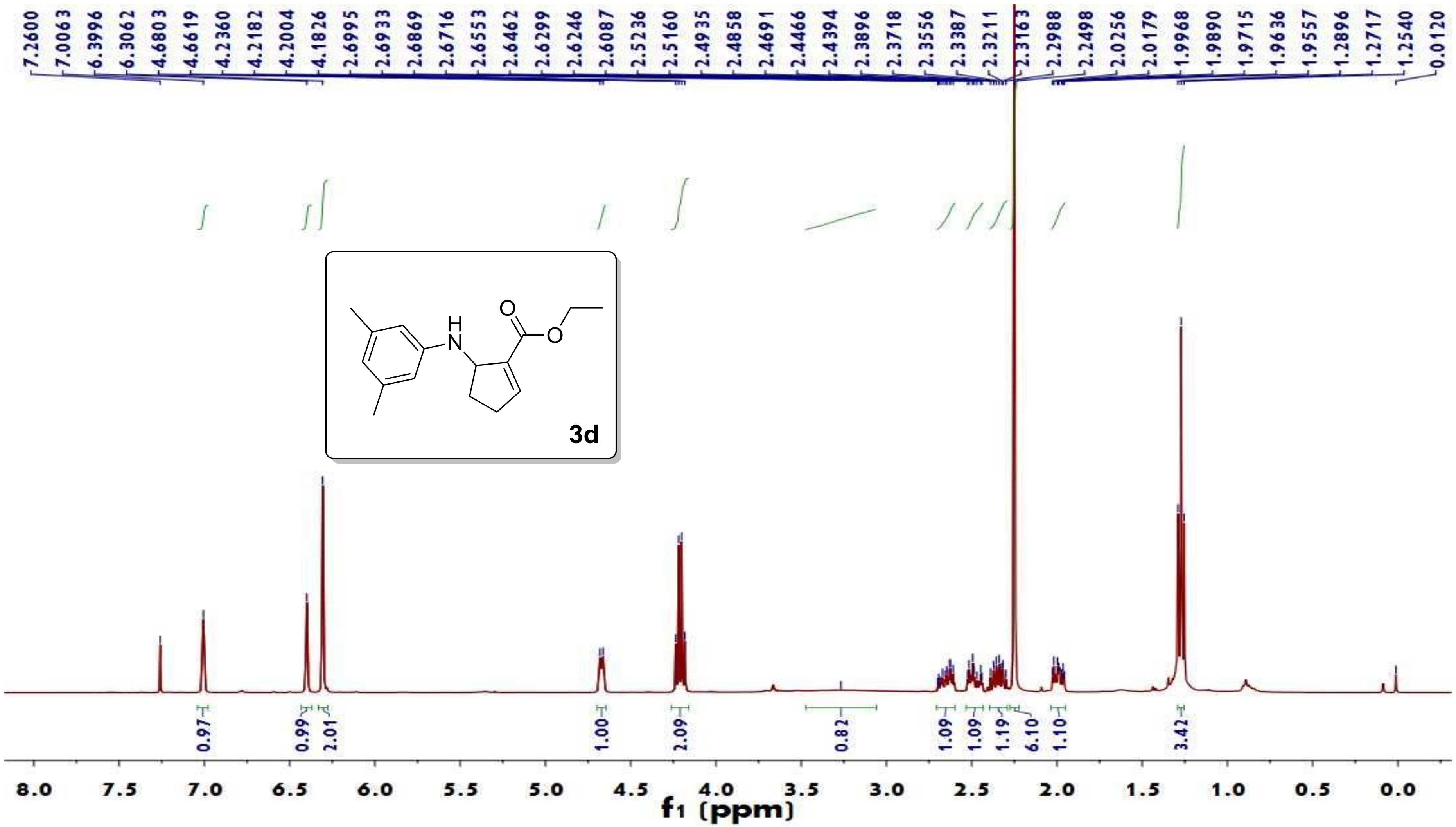

^{1}H NMR spectrum of 3d (400 MHz, $CDCl_3$).

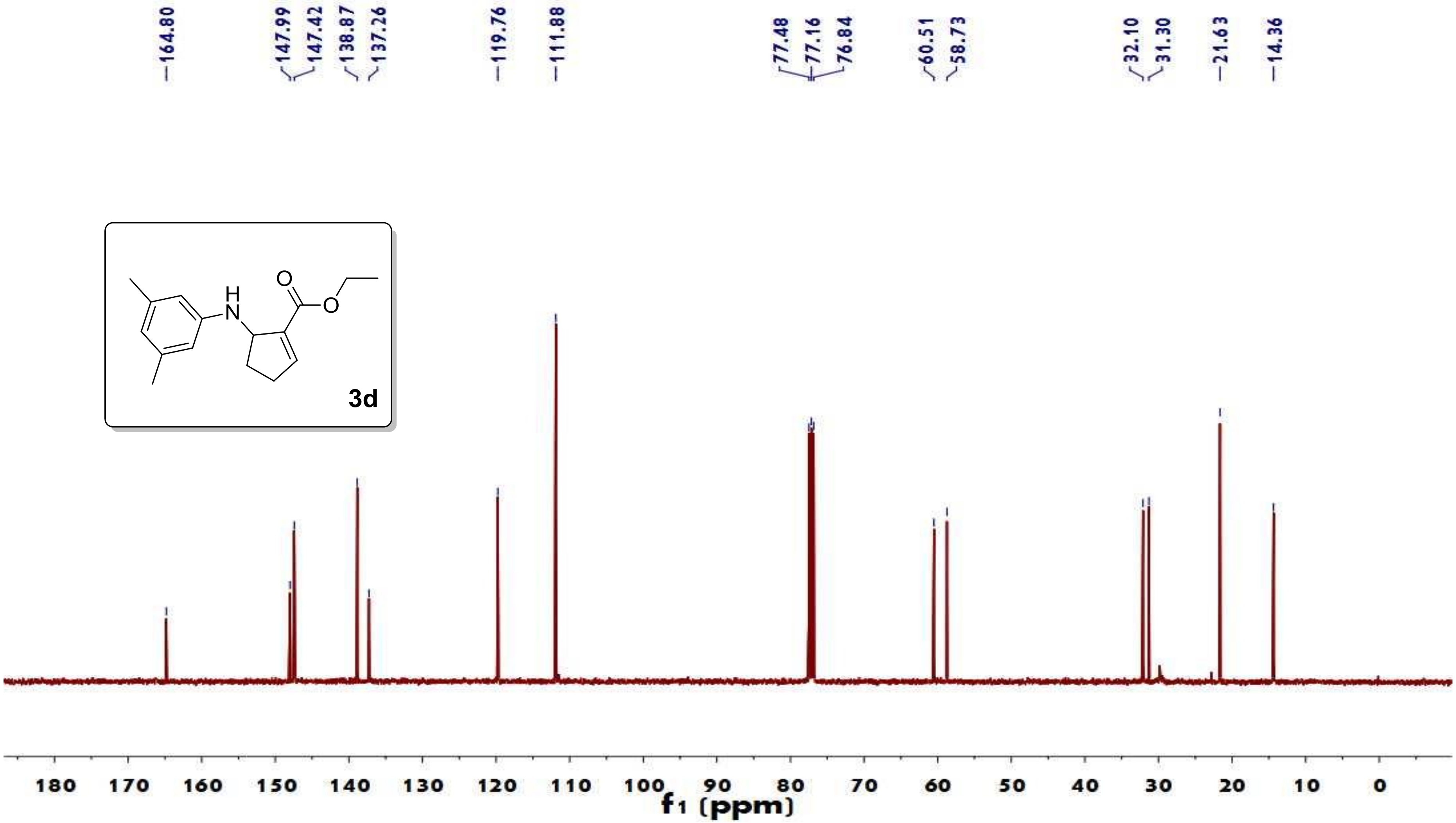

^{13}C NMR spectrum of 3d (400 MHz, $CDCl_3$).

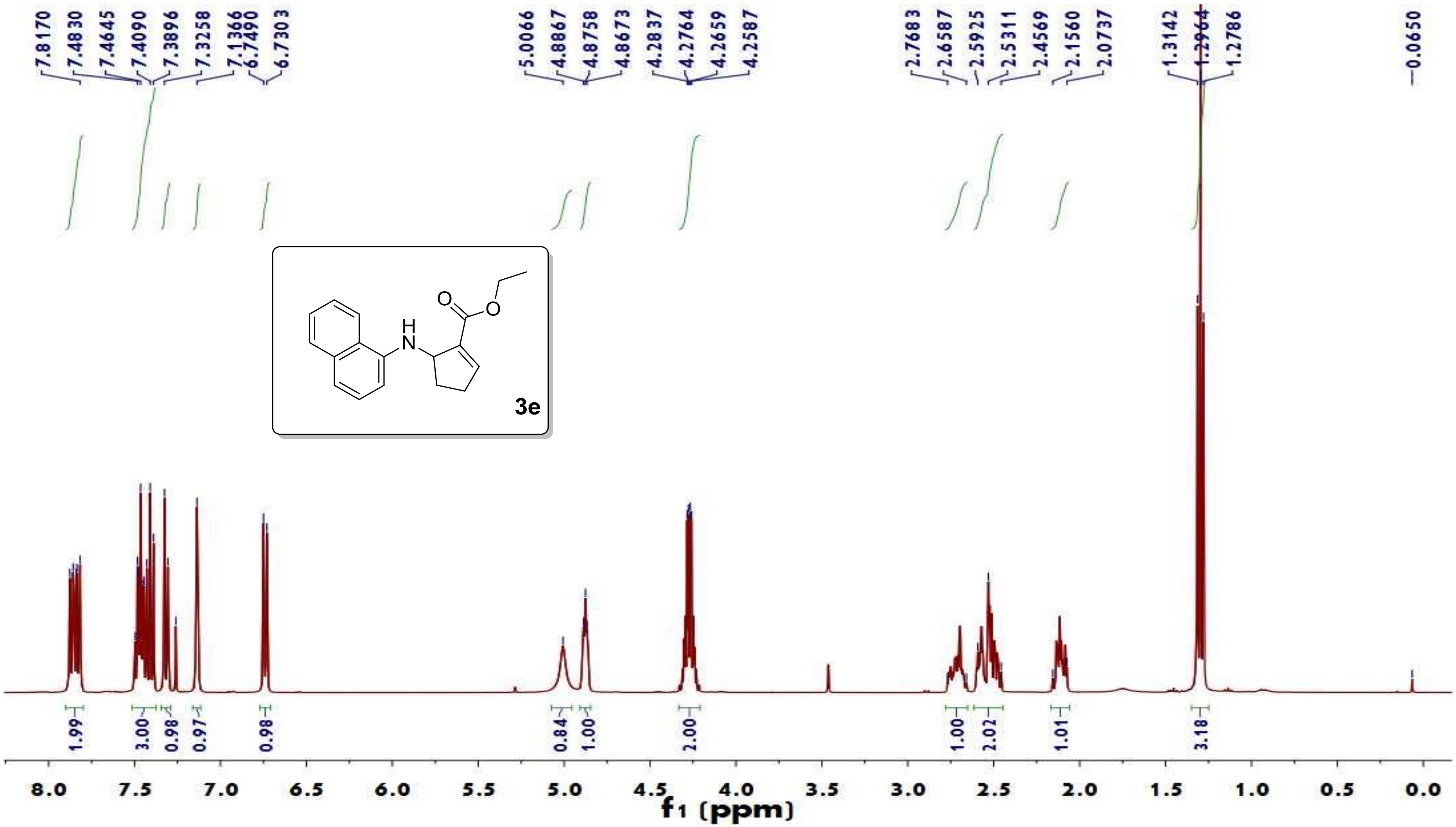

^{1}H NMR spectrum of 3e (400 MHz, $CDCl_3$).

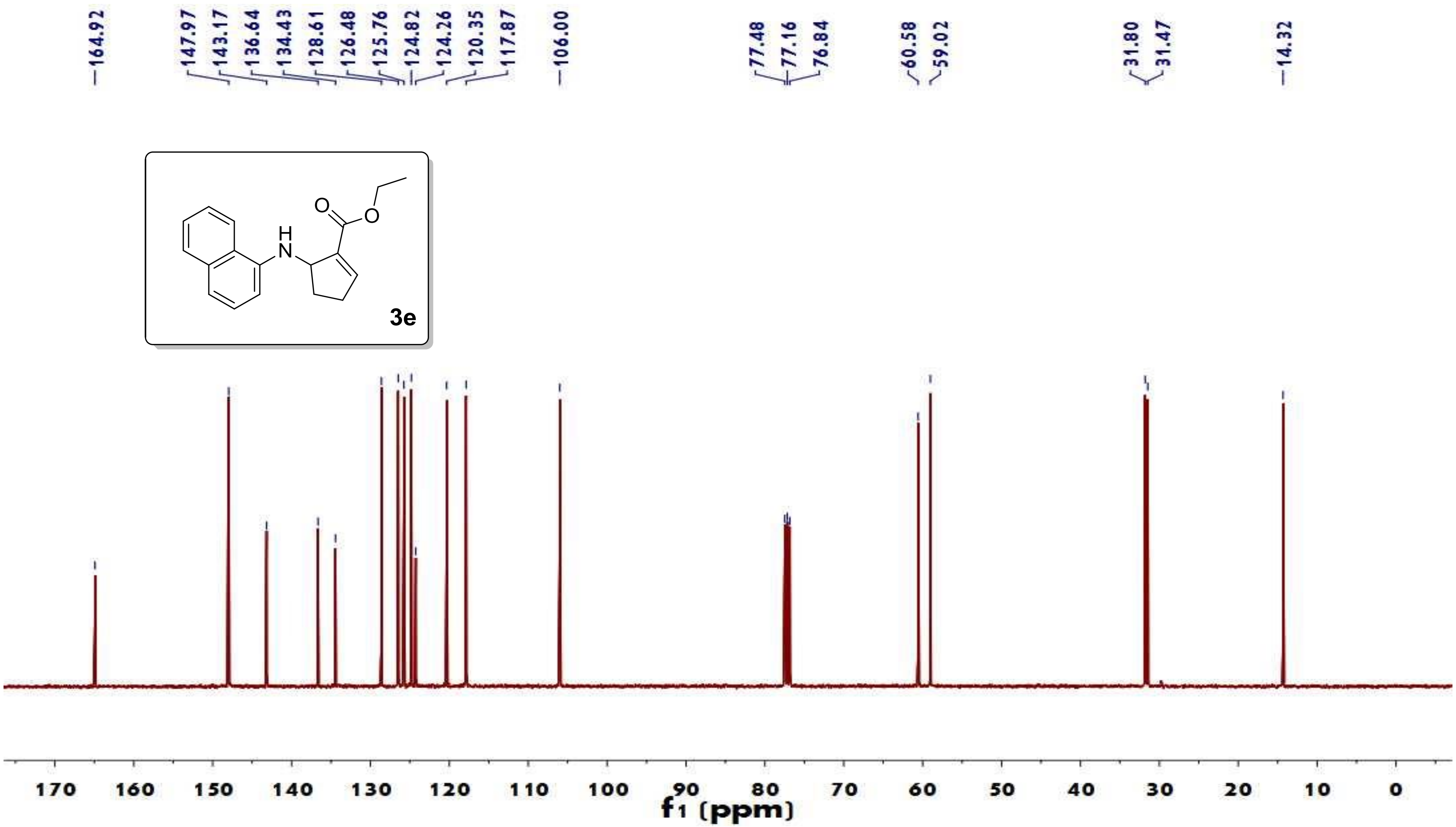

^{13}C NMR spectrum of 3e (400 MHz, CDCl$_3$).

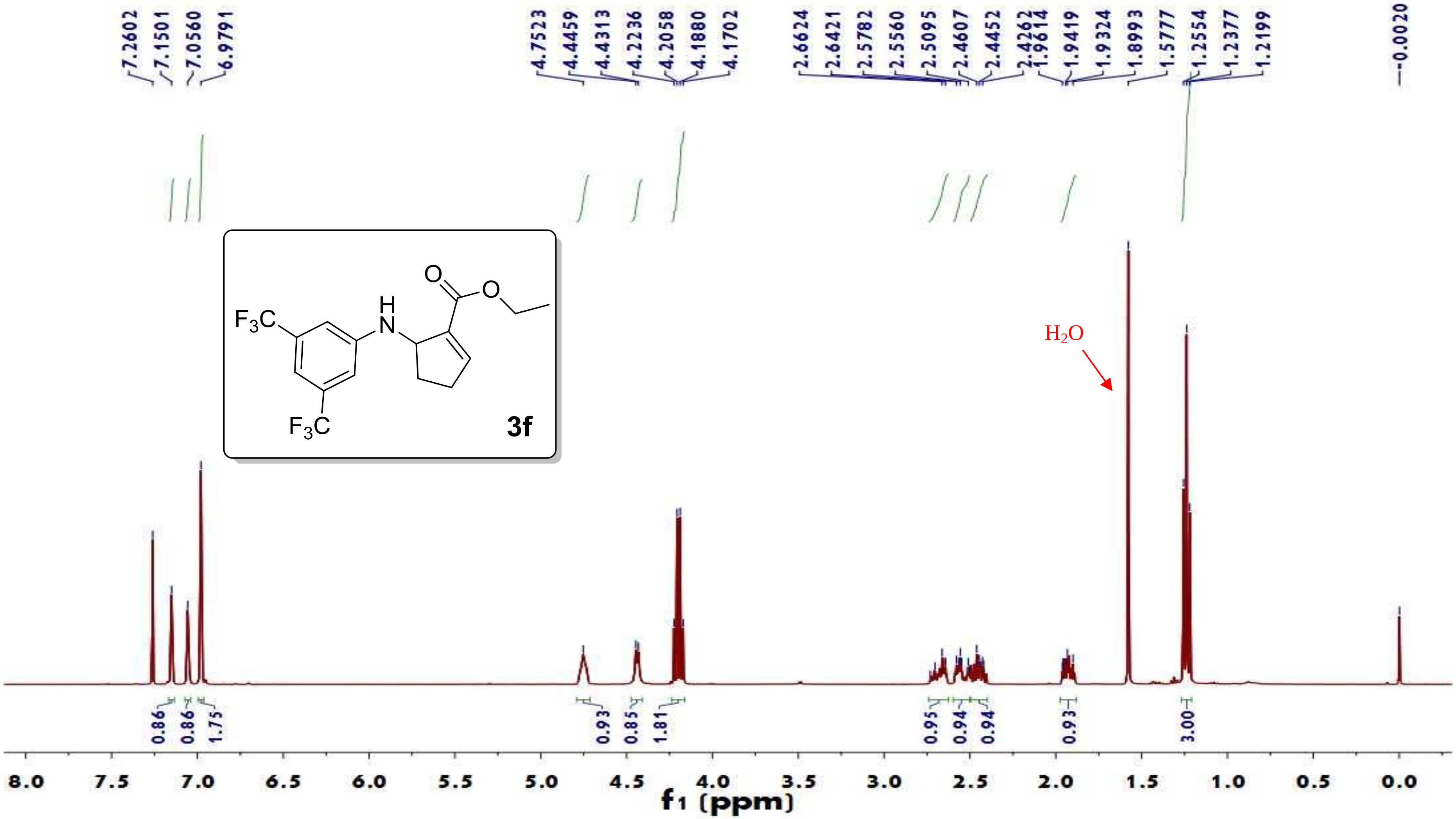

^{1}H NMR spectrum of 3f (400 MHz, $CDCl_3$).

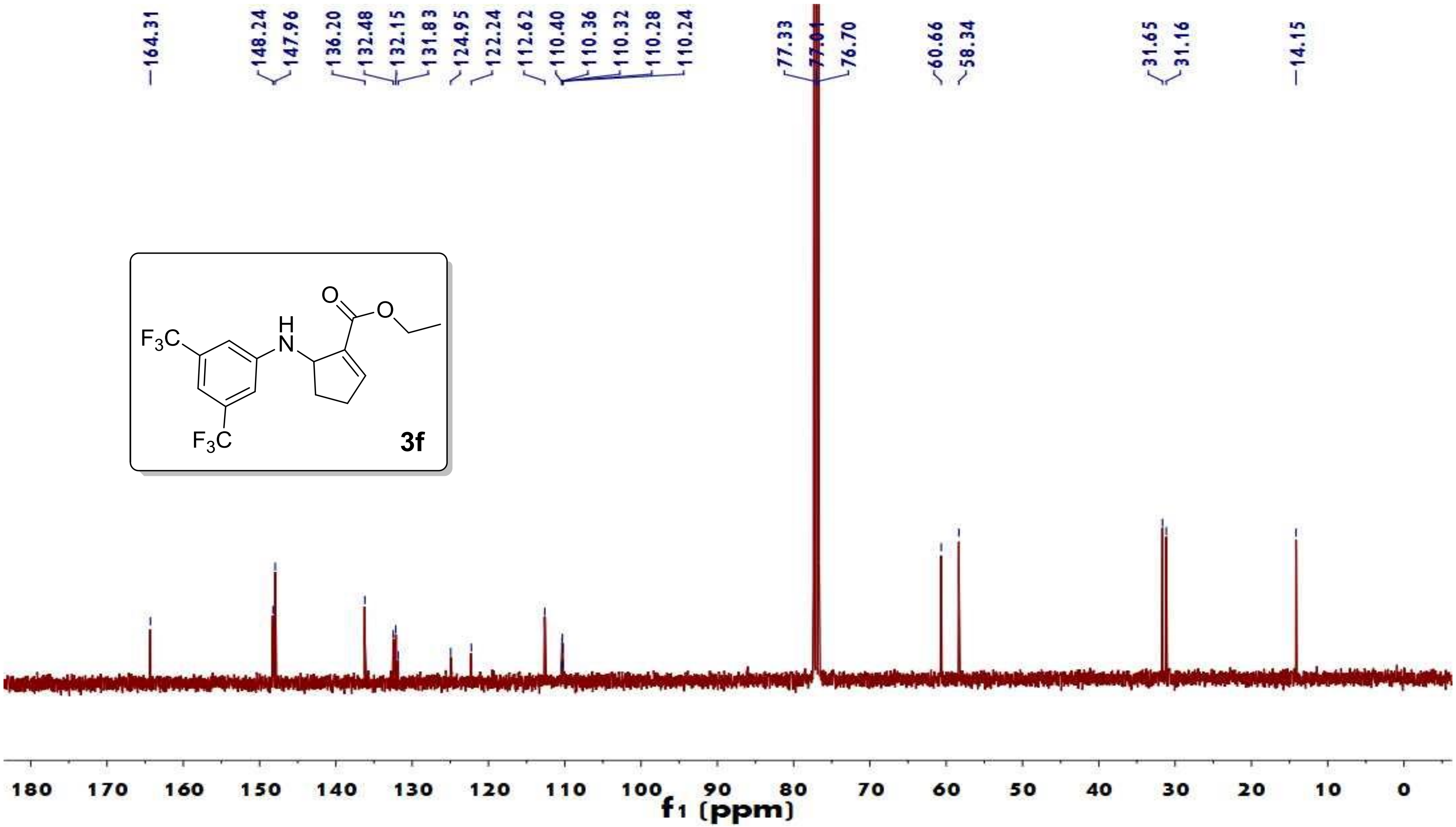

^{13}C NMR spectrum of 3f (400 MHz, CDCl$_3$).

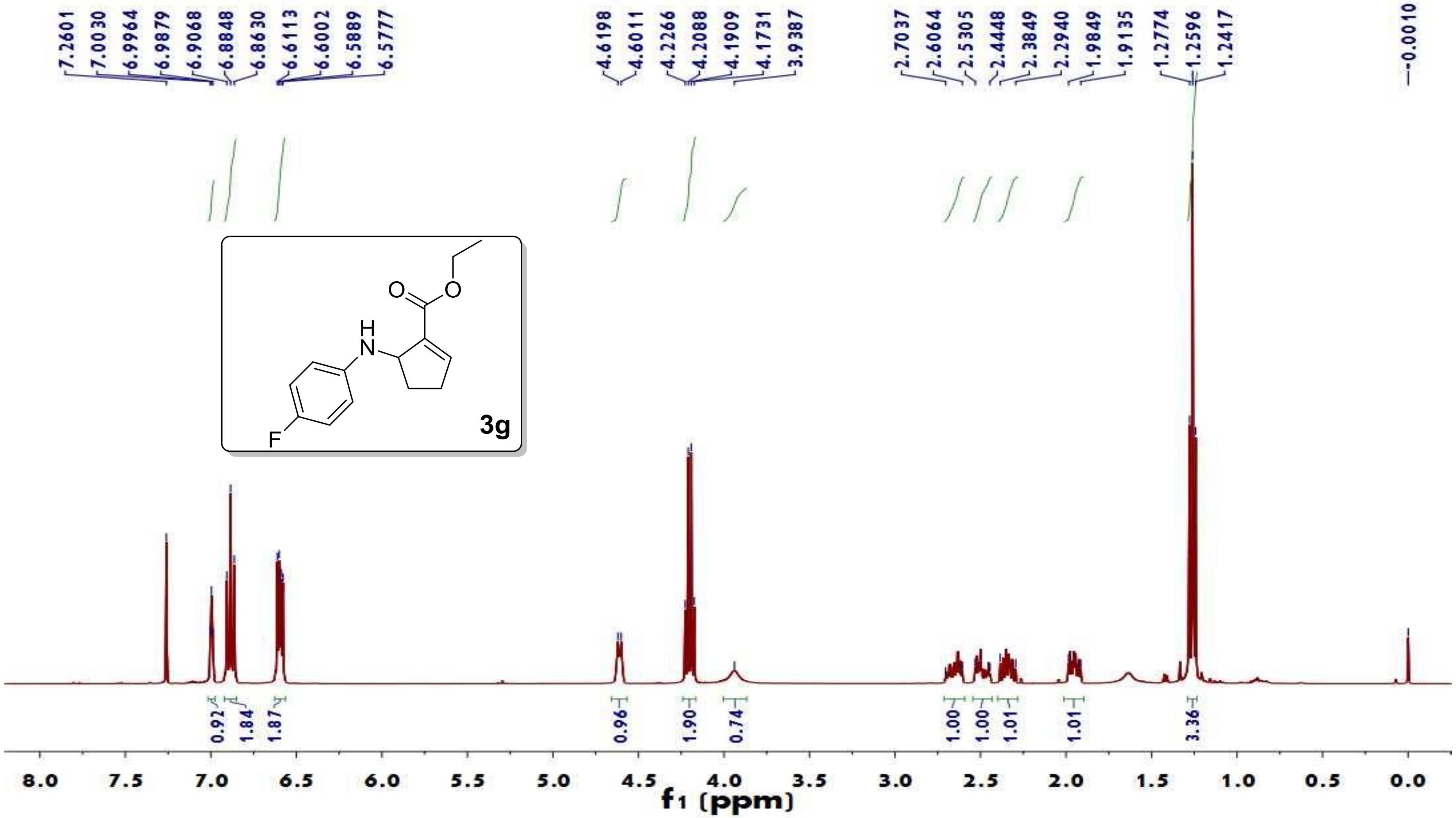

^{1}H NMR spectrum of 3g (400 MHz, $CDCl_3$).

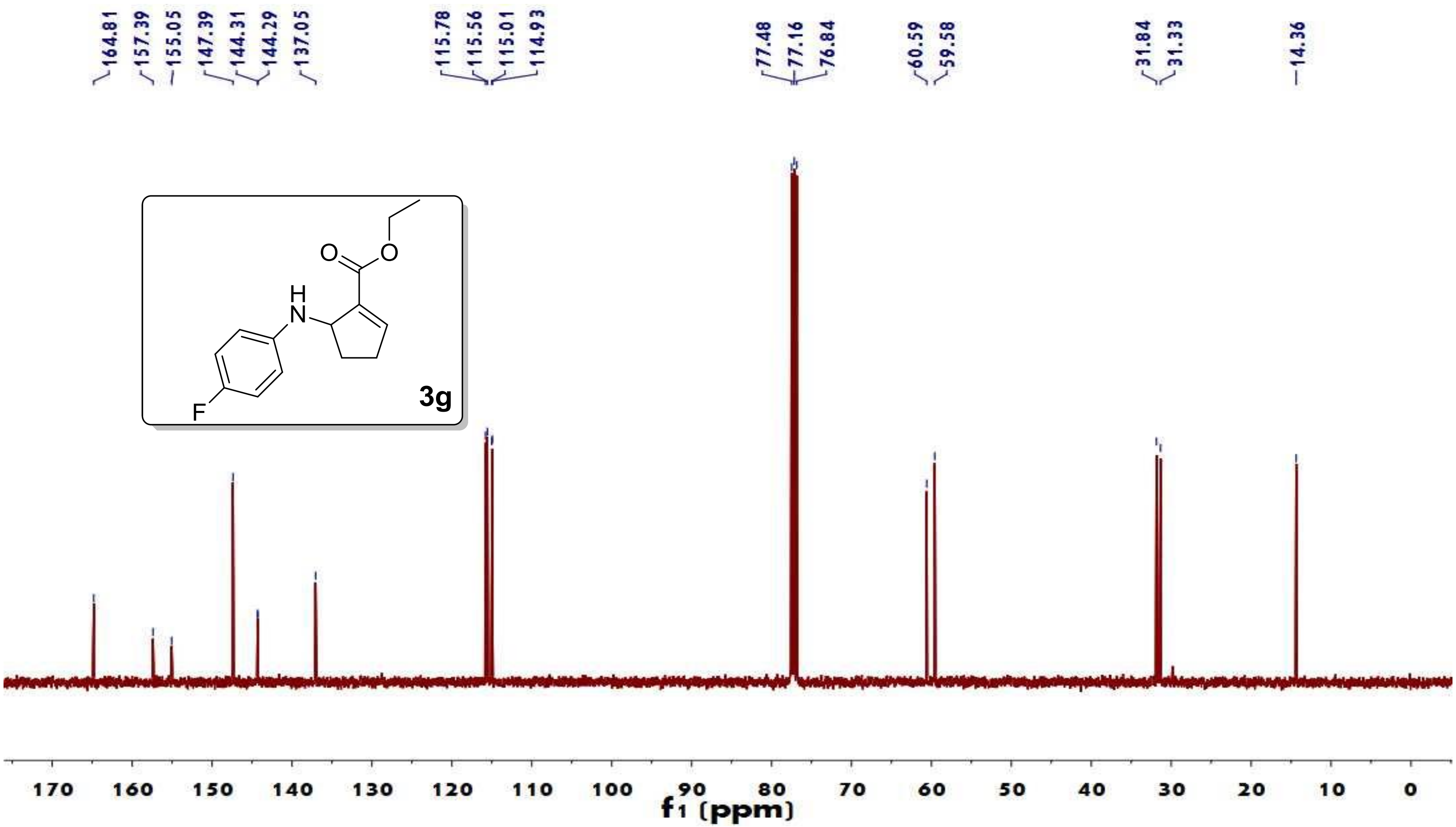

^{13}C NMR spectrum of 3g (400 MHz, $CDCl_3$).

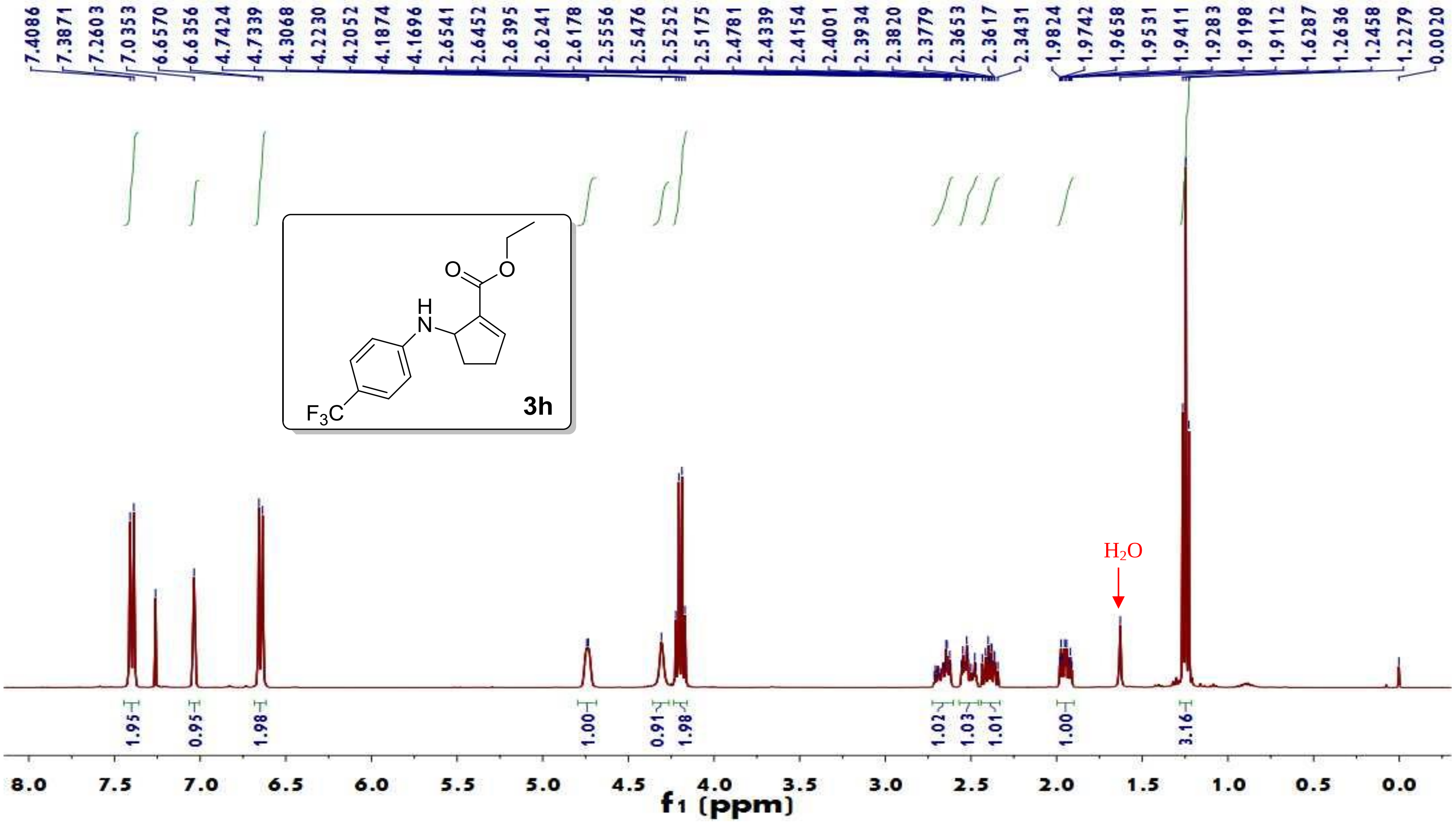

^{1}H NMR spectrum of 3h (400 MHz, $CDCl_3$).

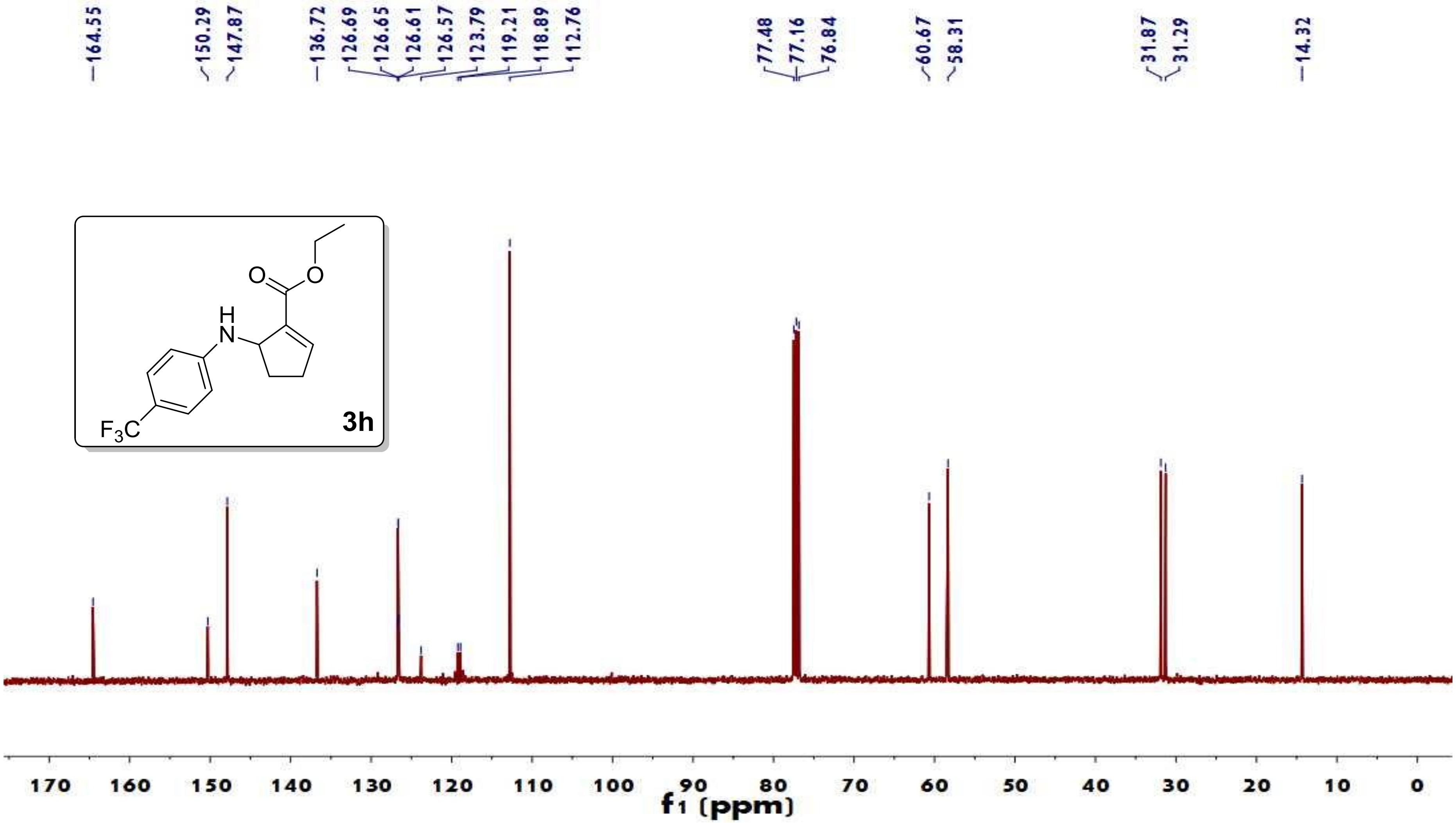

^{13}C NMR spectrum of 3h (400 MHz, $CDCl_3$).

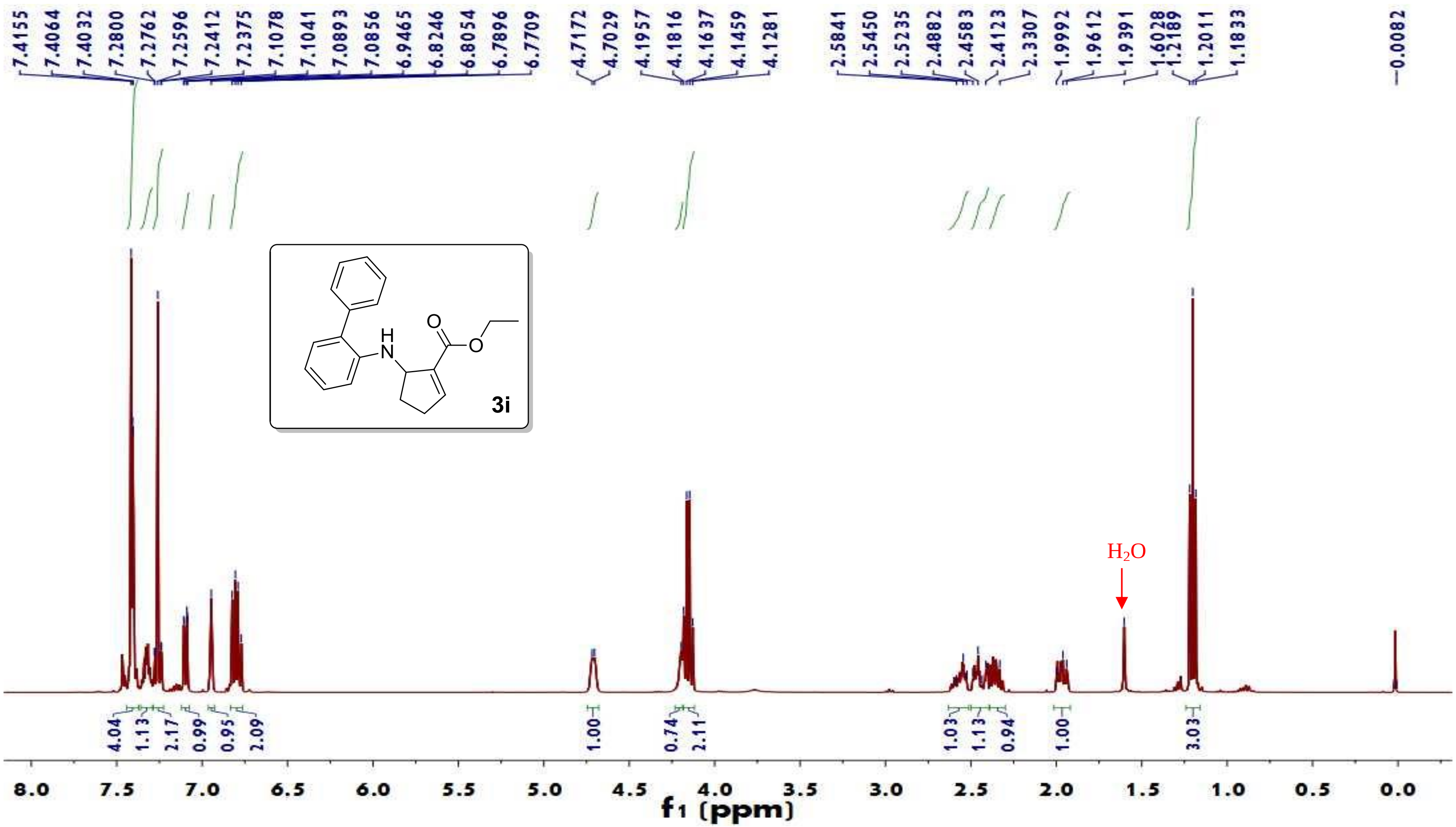

^{1}H NMR spectrum of 3i (400 MHz, $CDCl_3$).

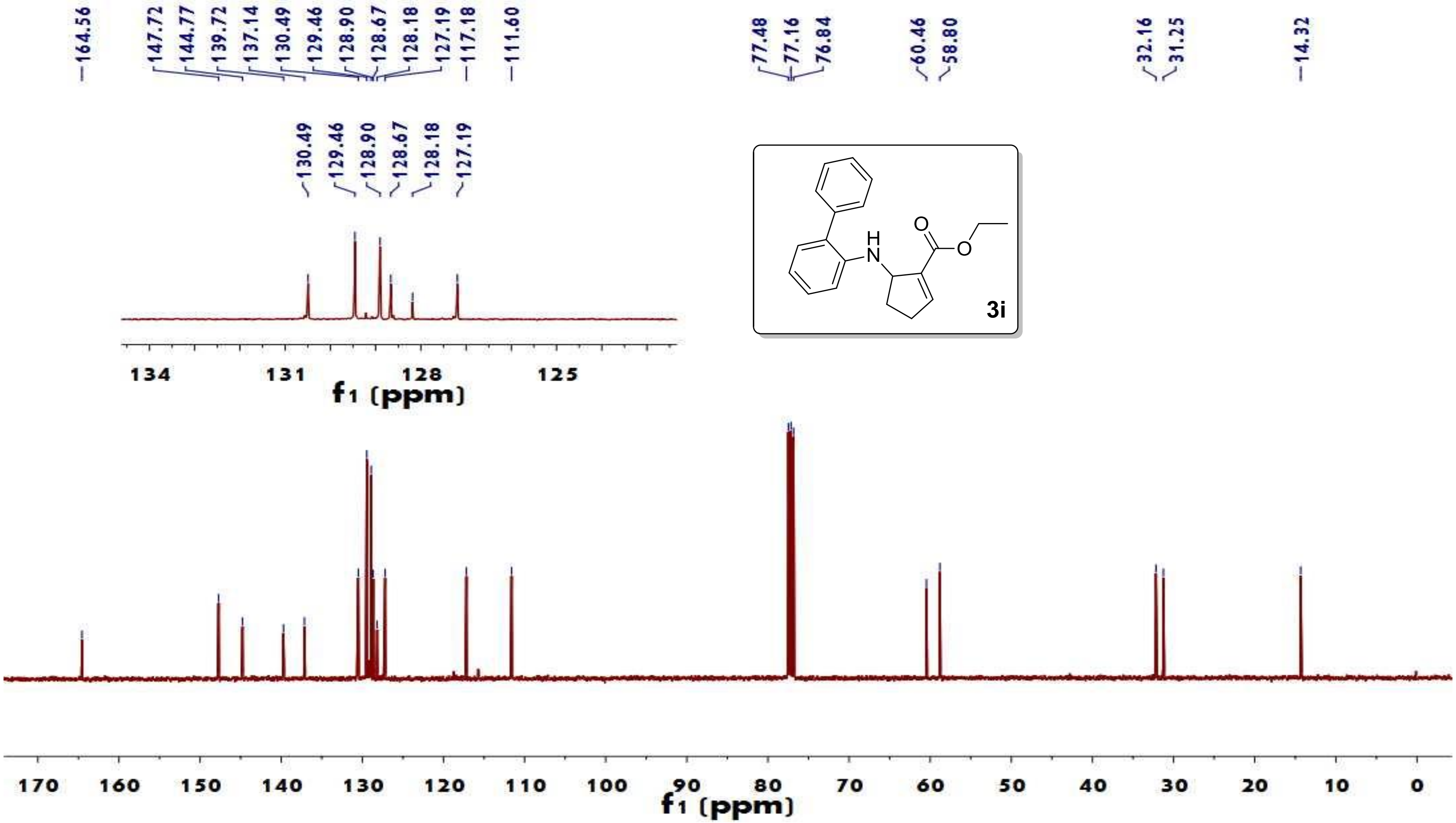

^{13}C NMR spectrum of 3i (400 MHz, $CDCl_3$).

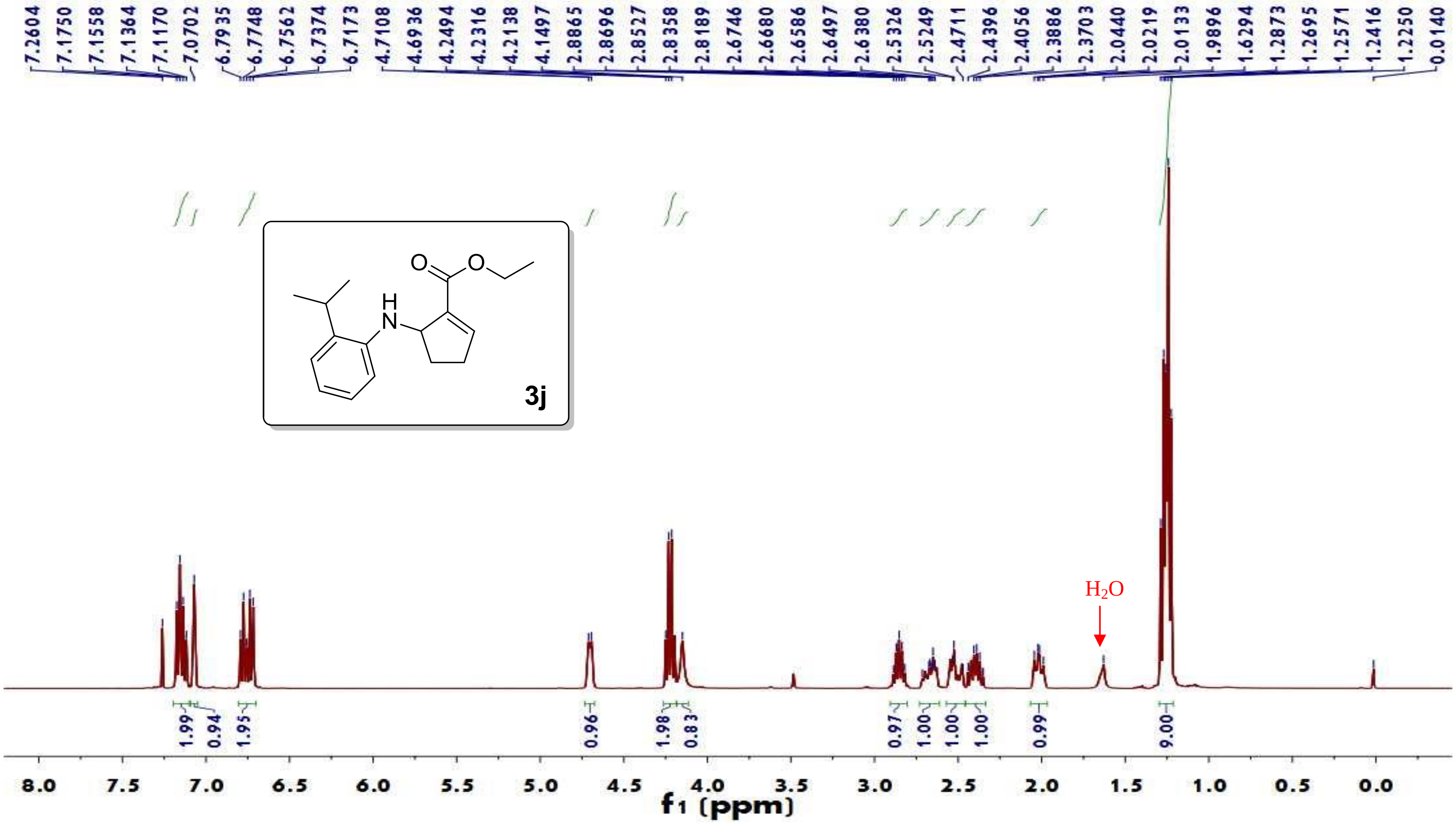

^{1}H NMR spectrum of 3j (400 MHz, $CDCl_3$).

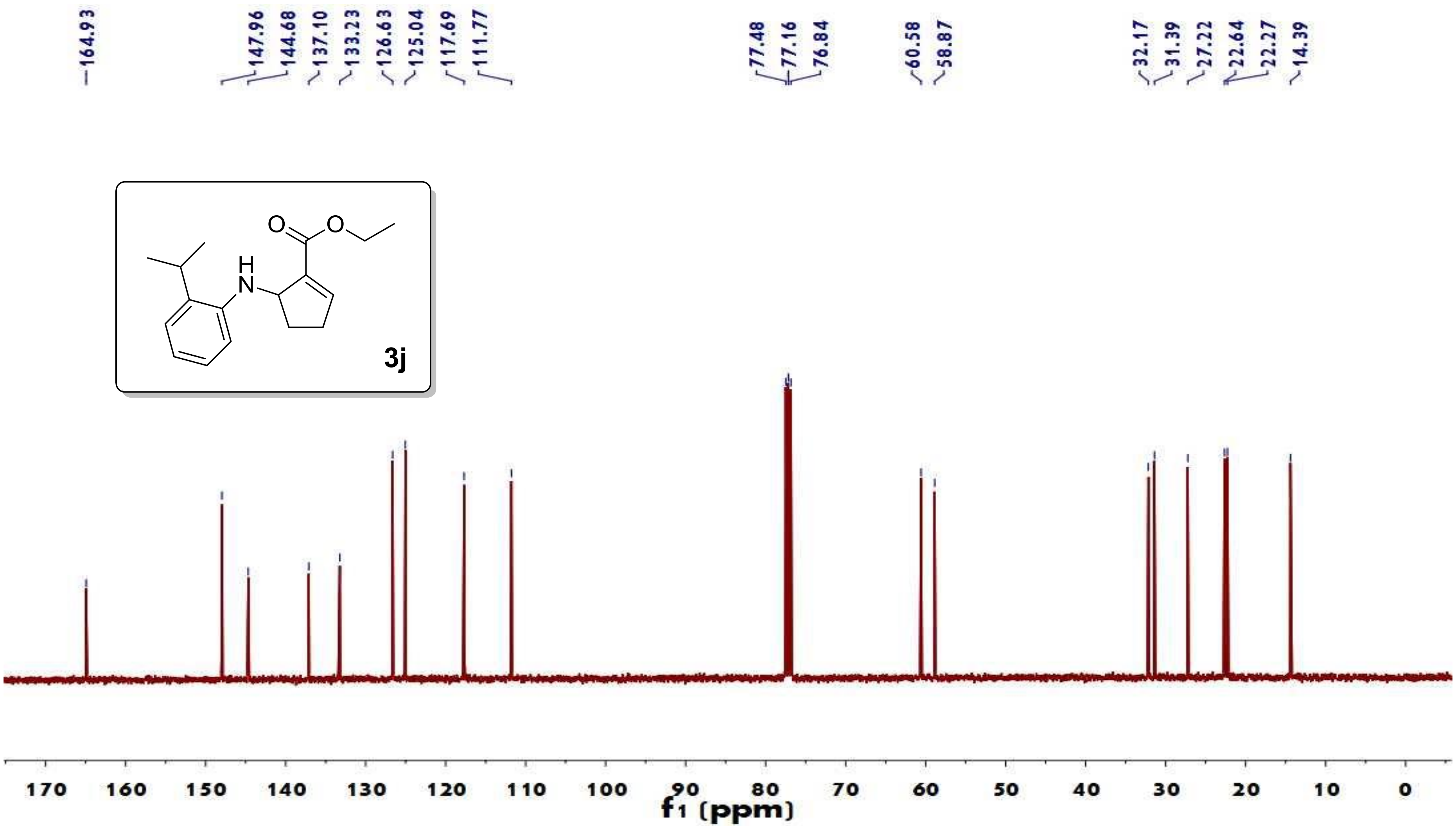

^{13}C NMR spectrum of 3j (400 MHz, $CDCl_3$).

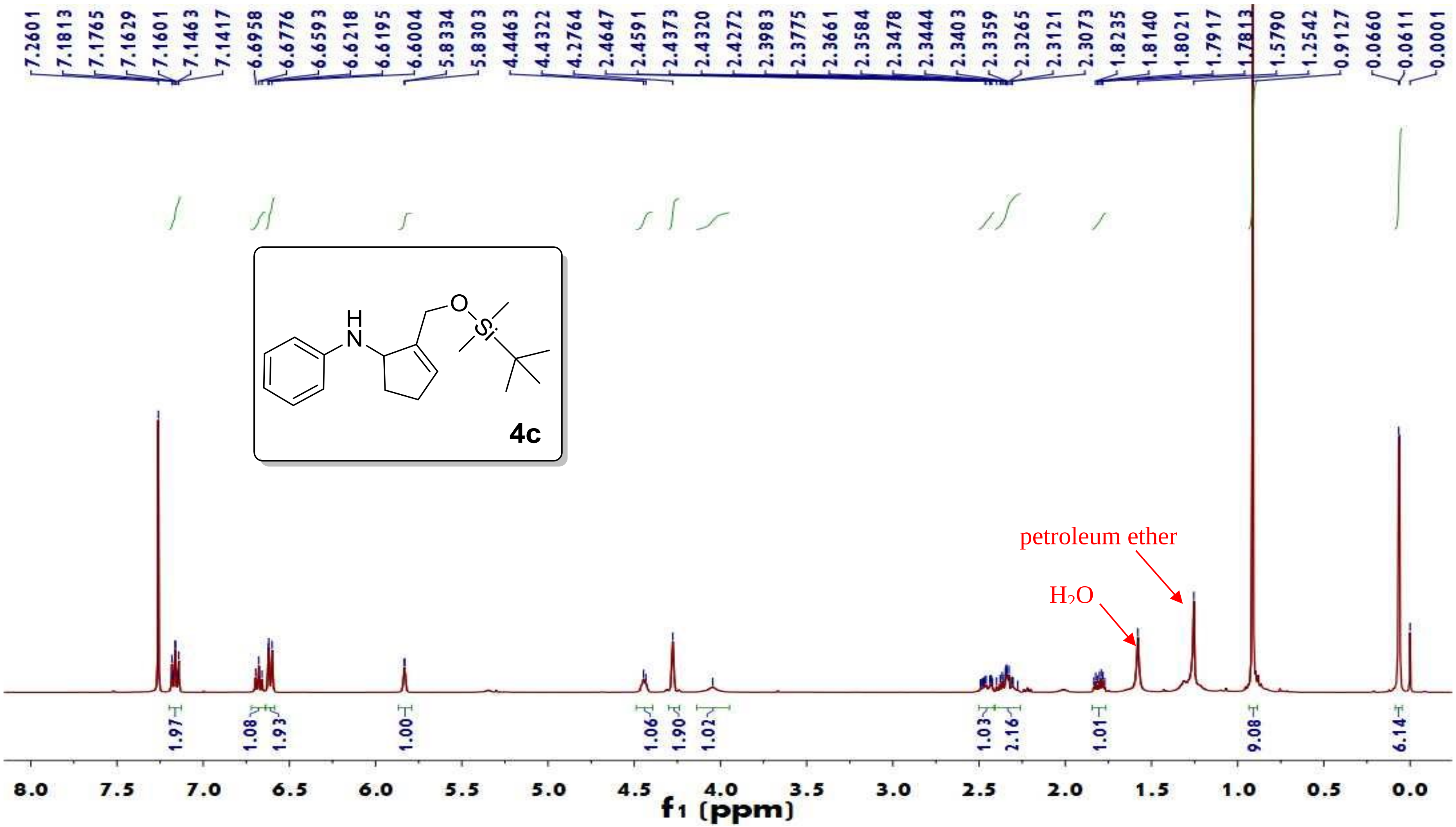

^{1}H NMR spectrum of 4c (400 MHz, $CDCl_3$).

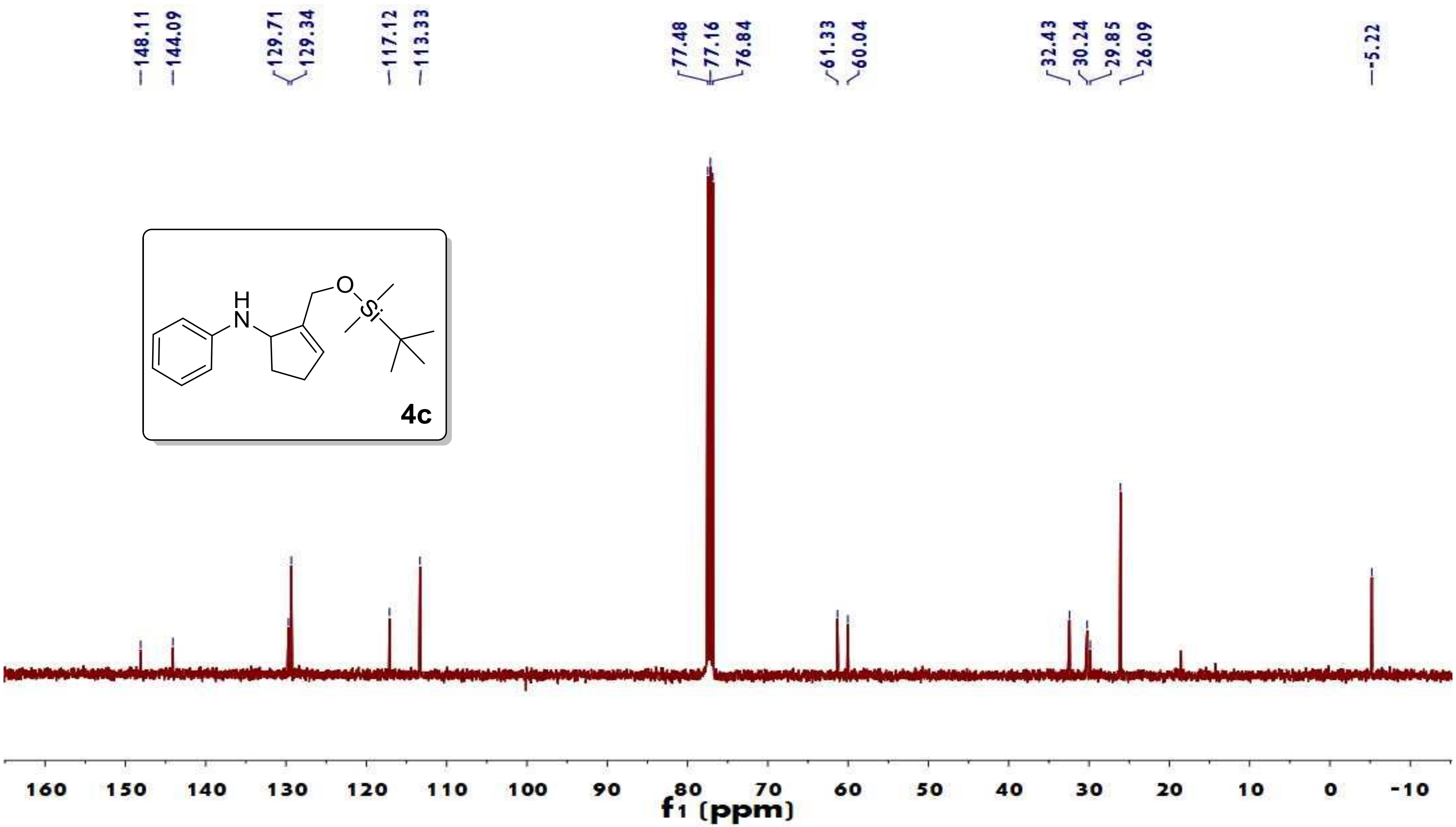

^{13}C NMR spectrum of 4c (400 MHz, $CDCl_3$).

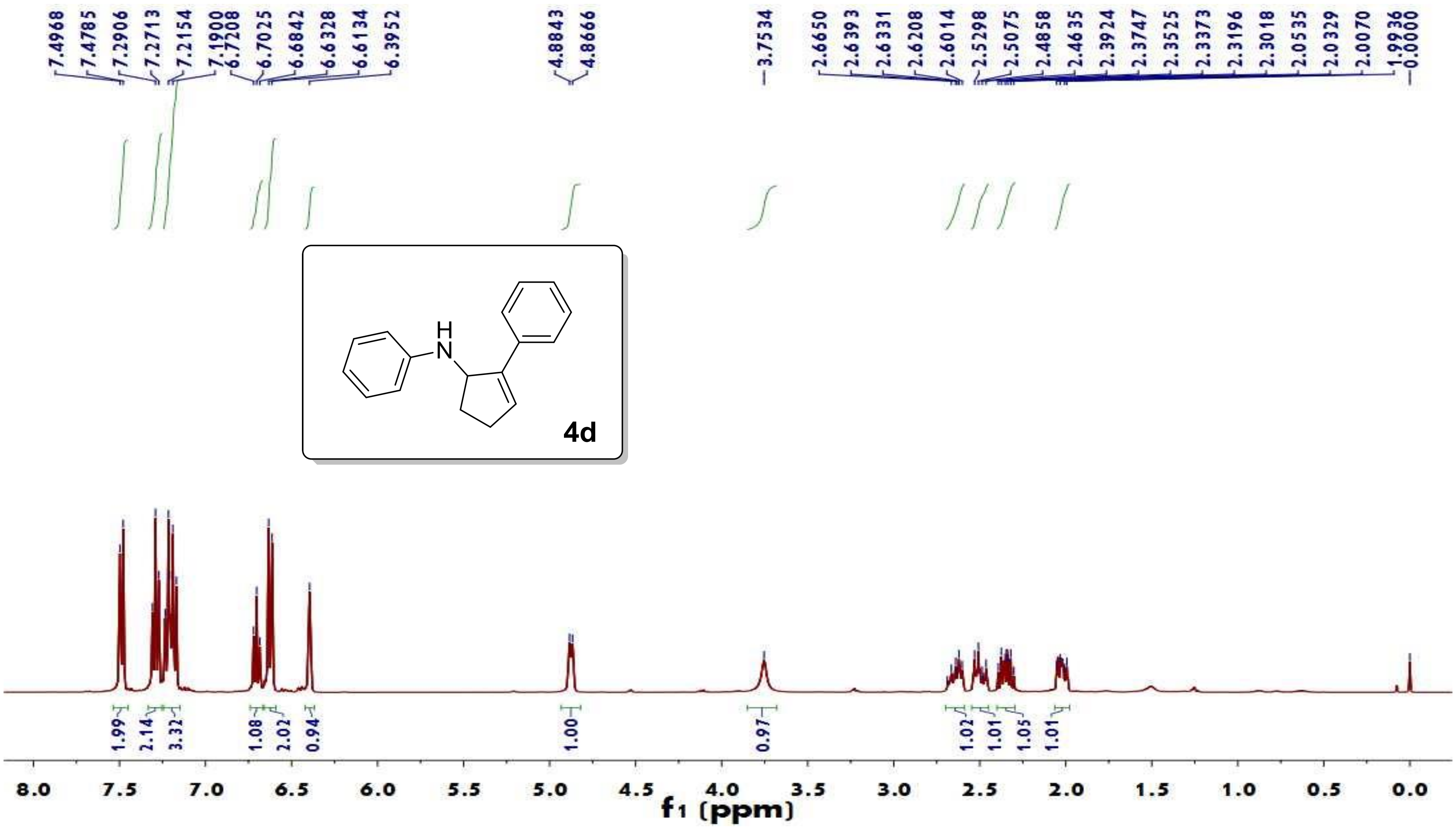

^{1}H NMR spectrum of 4d (400 MHz, $CDCl_3$).

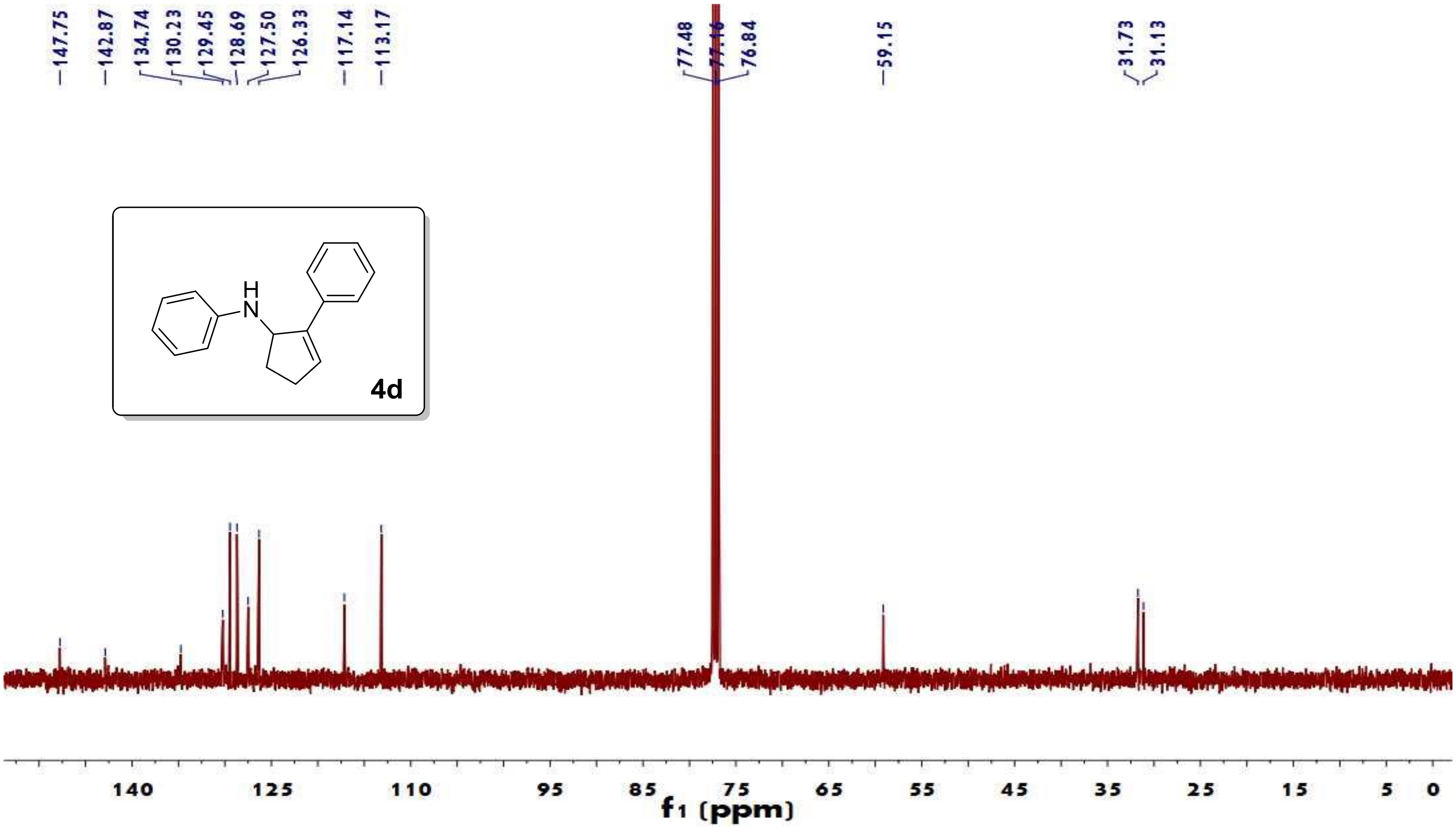

^{13}C NMR spectrum of 4d (400 MHz, $CDCl_3$).

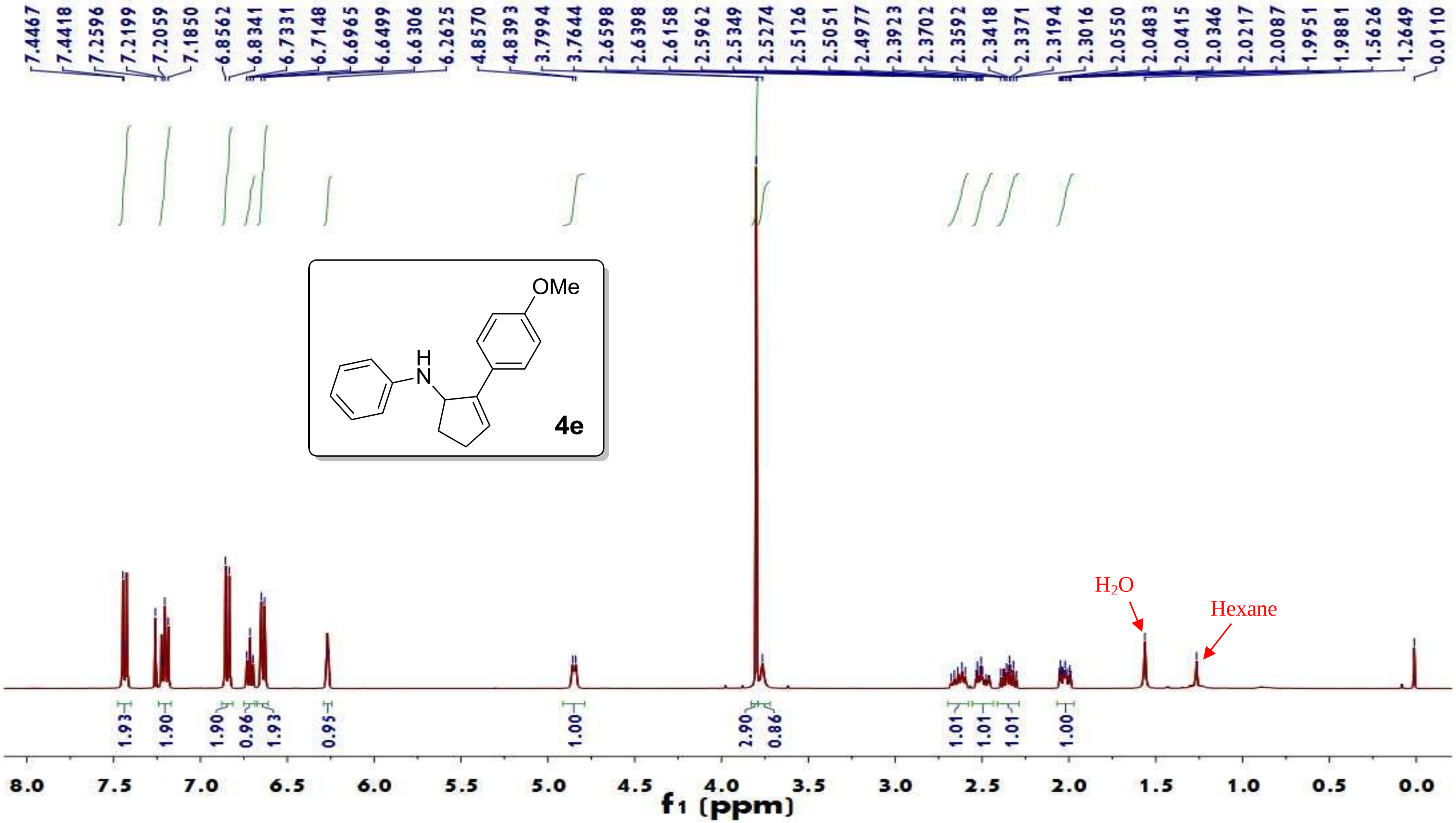

^{1}H NMR spectrum of 4e (400 MHz, $CDCl_3$).

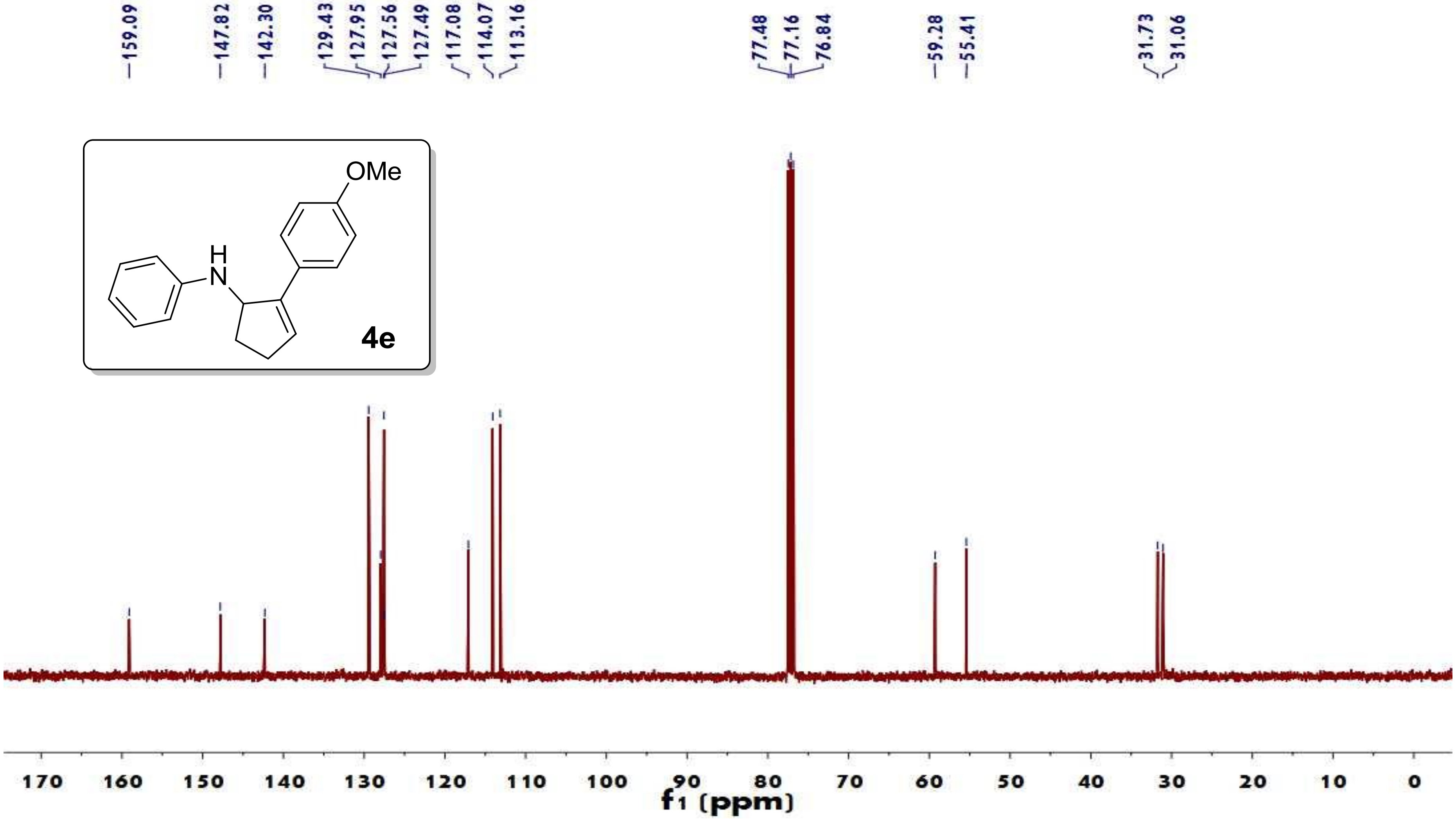

^{13}C NMR spectrum of 4e (400 MHz, $CDCl_3$).

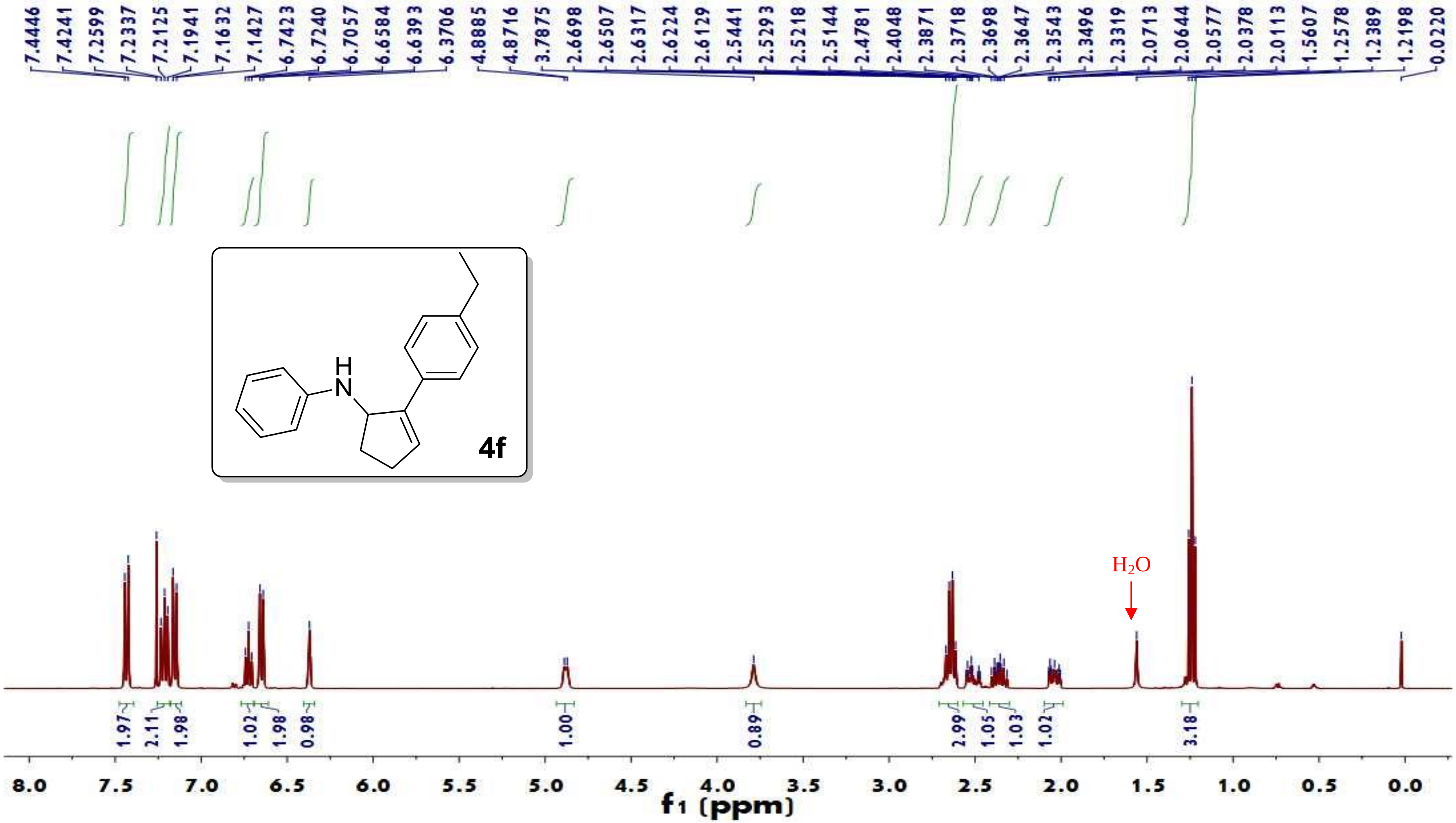

^{1}H NMR spectrum of 4f (400 MHz, $CDCl_3$).

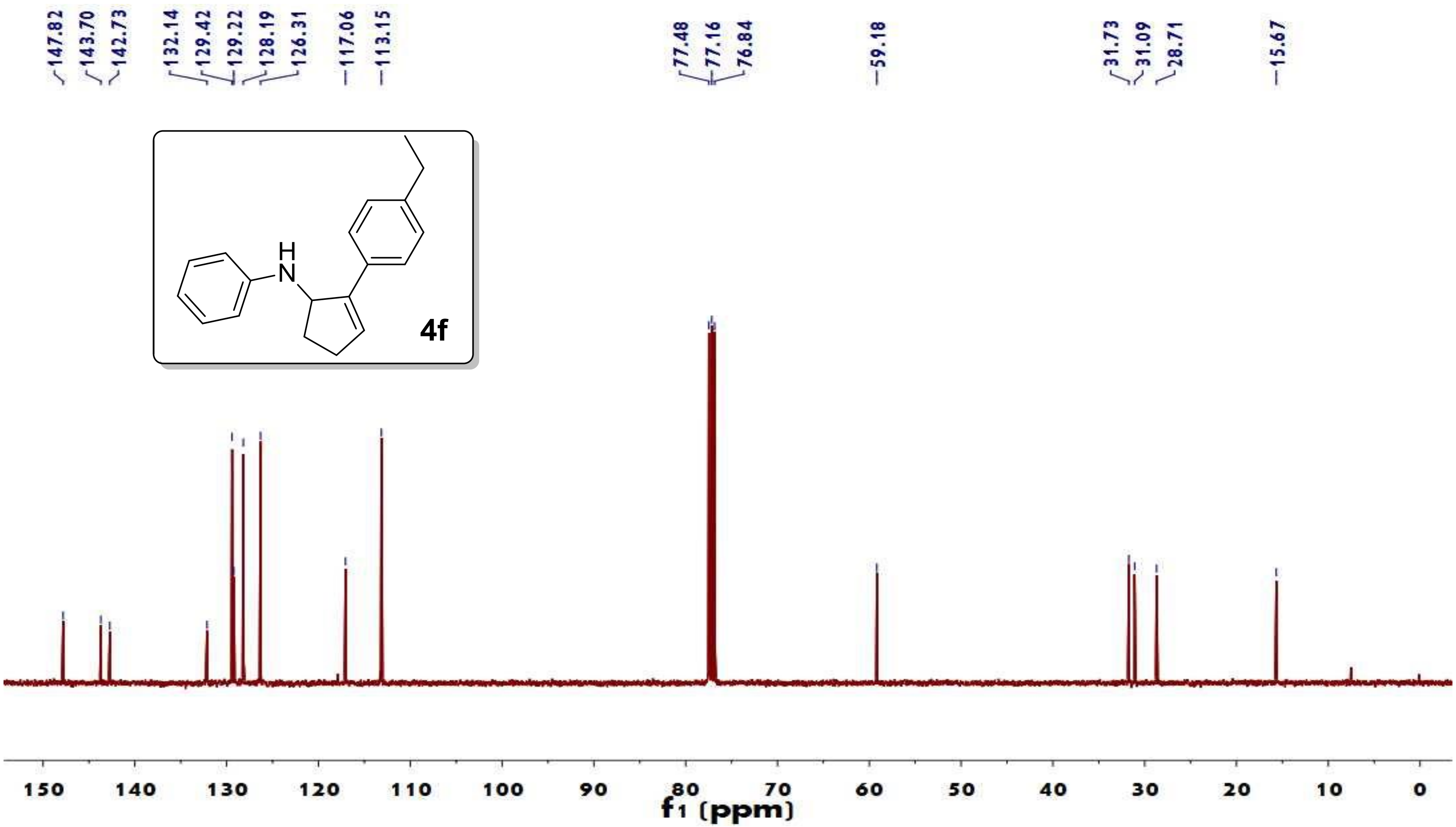

^{13}C NMR spectrum of 4f (400 MHz, $CDCl_3$).

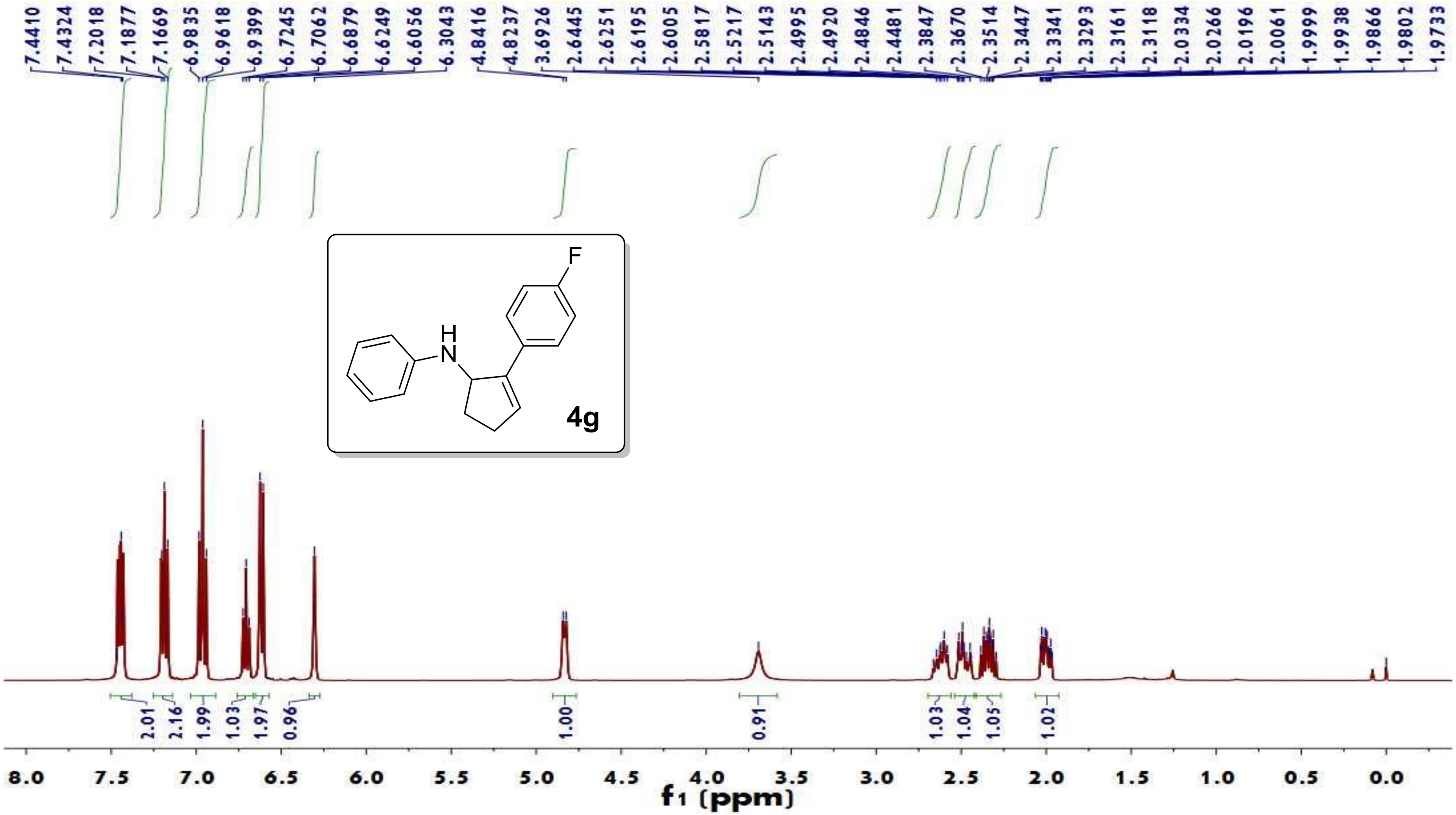

^{1}H NMR spectrum of 4g (400 MHz, $CDCl_3$).

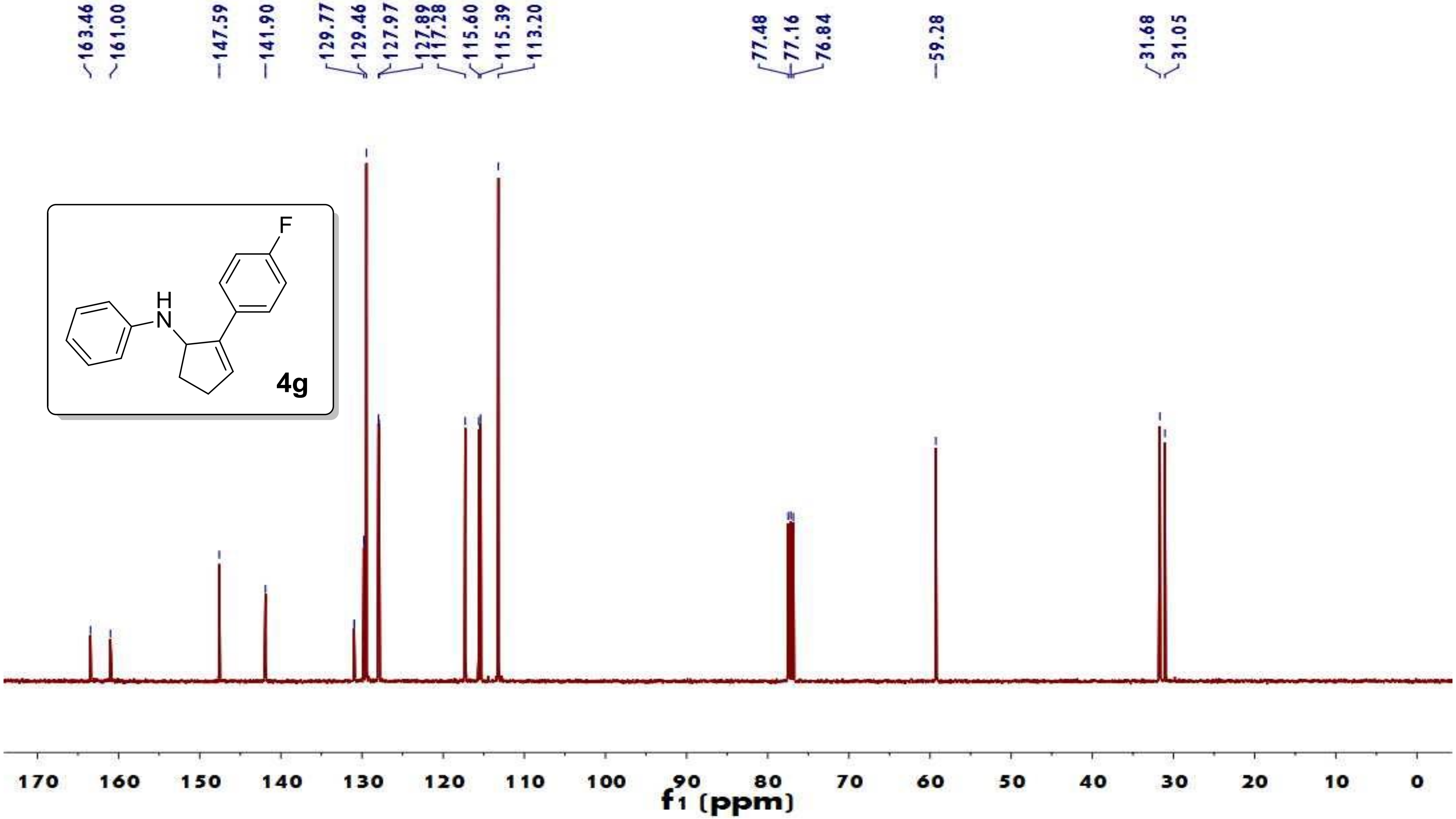

^{13}C NMR spectrum of 4g (400 MHz, CDCl$_3$.

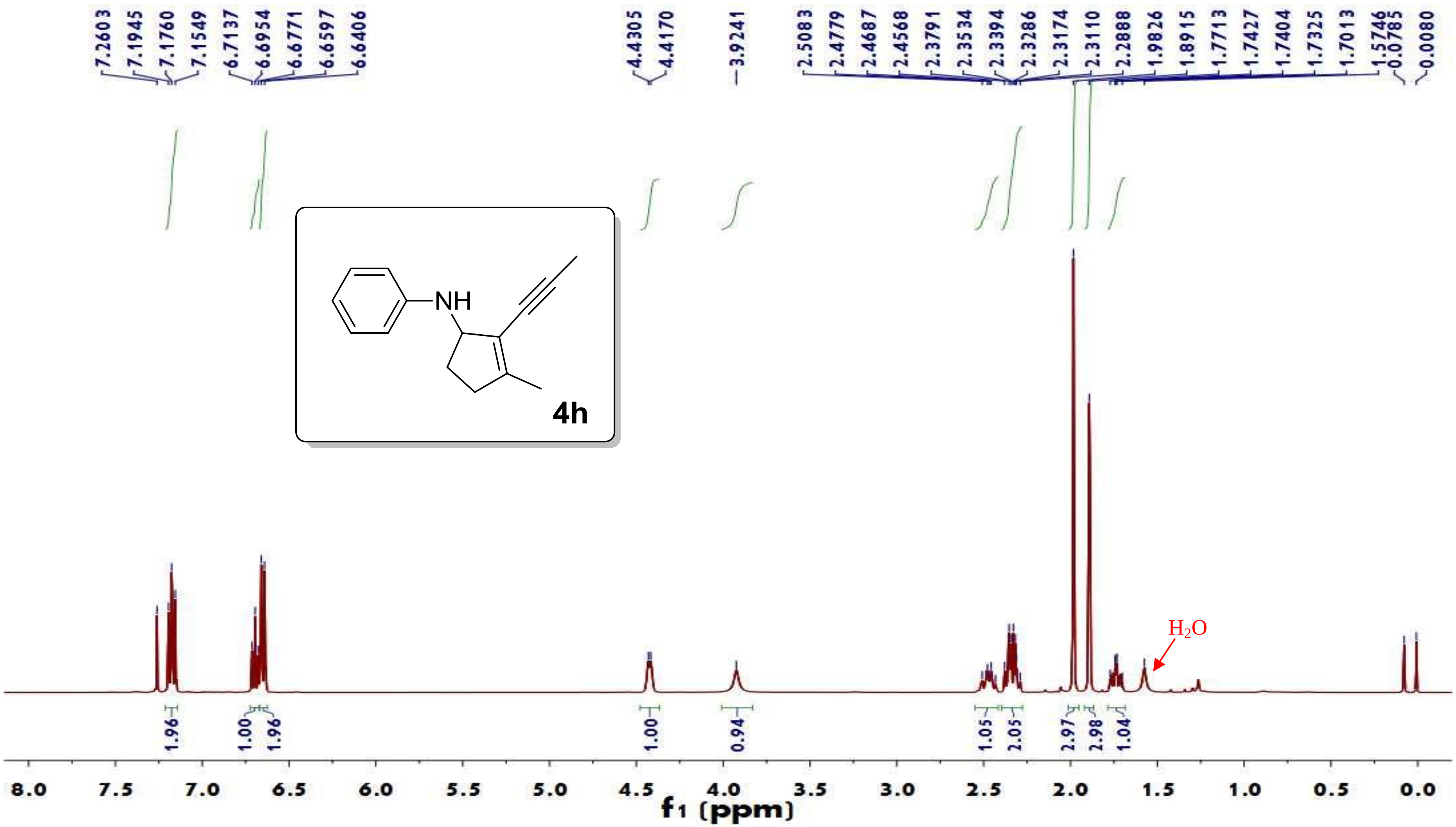

^{1}H NMR spectrum of 4h (400 MHz, $CDCl_3$).

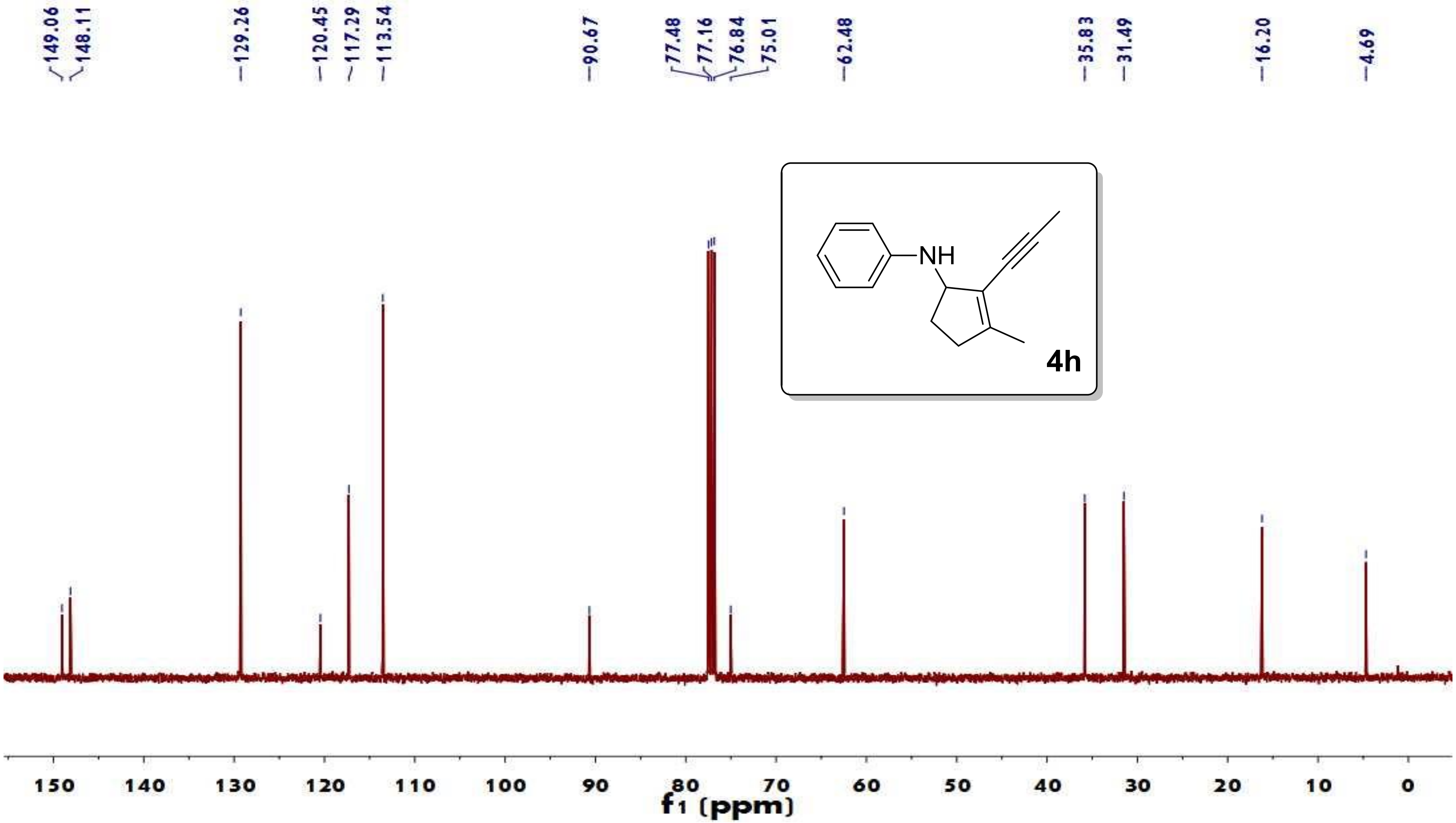

^{13}C NMR spectrum of 4h (400 MHz, $CDCl_3$).

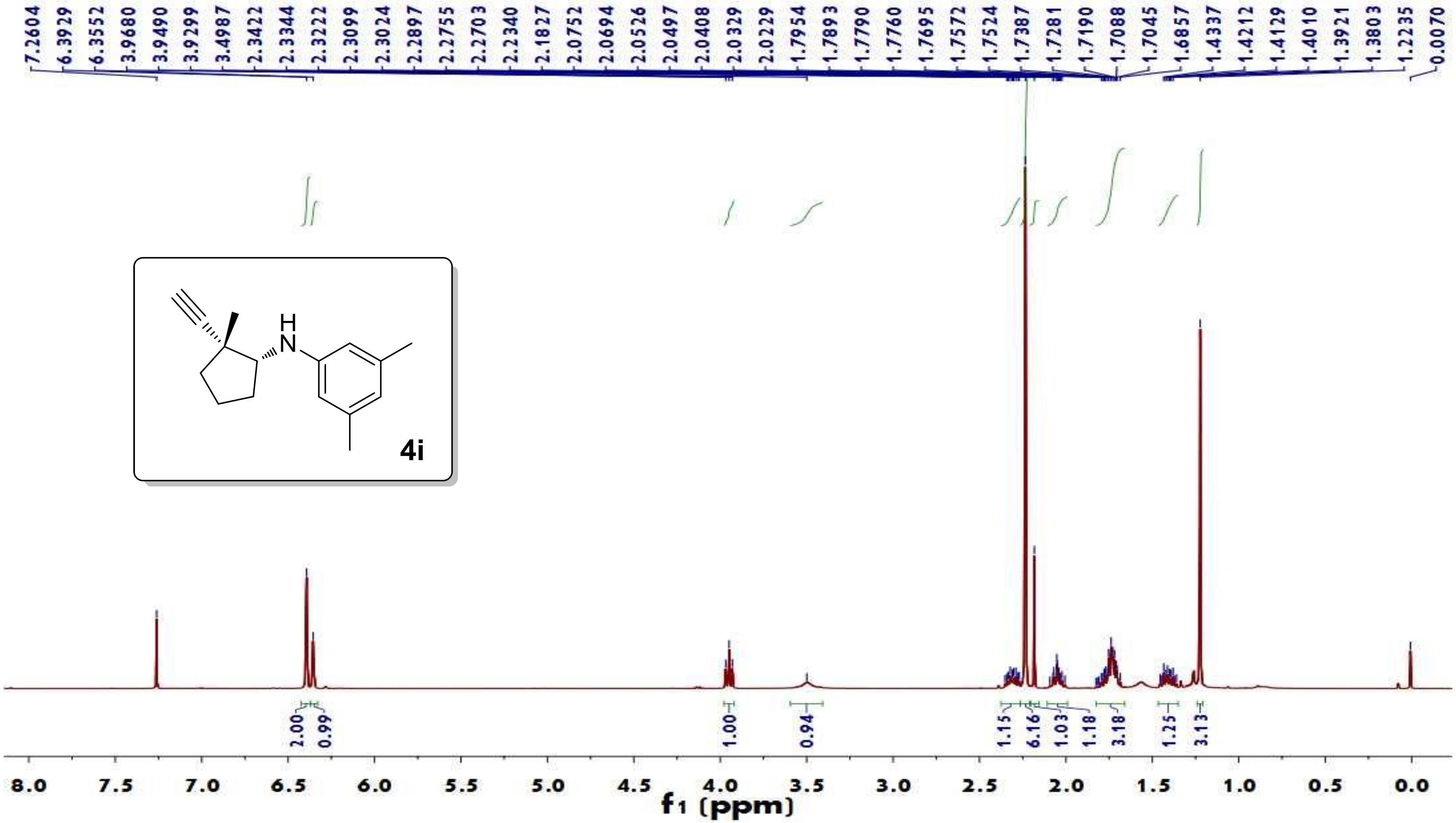

^{1}H NMR spectrum of 4i (400 MHz, $CDCl_3$).

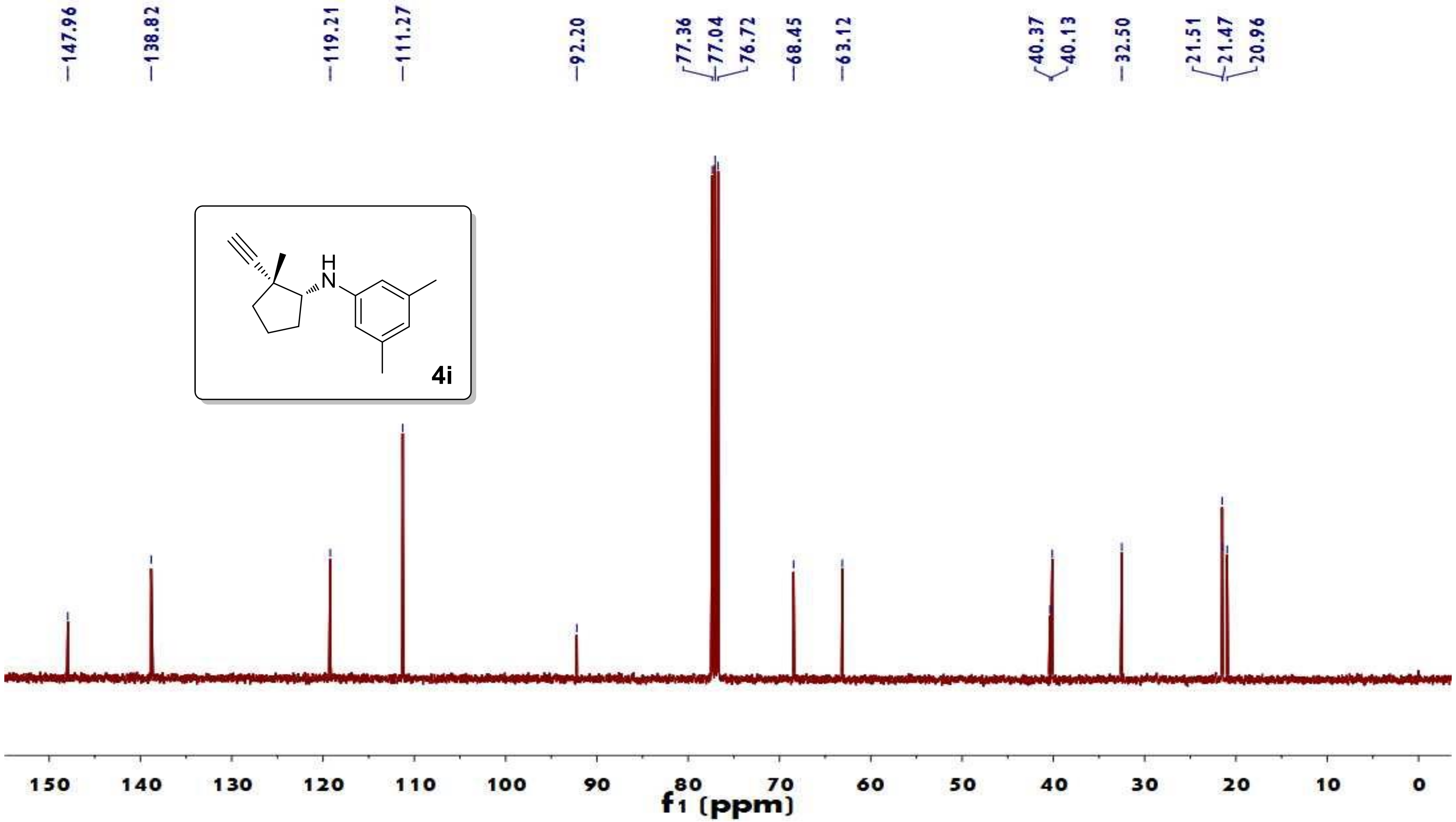

^{13}C NMR spectrum of 4i (400 MHz, CDCl$_3$.

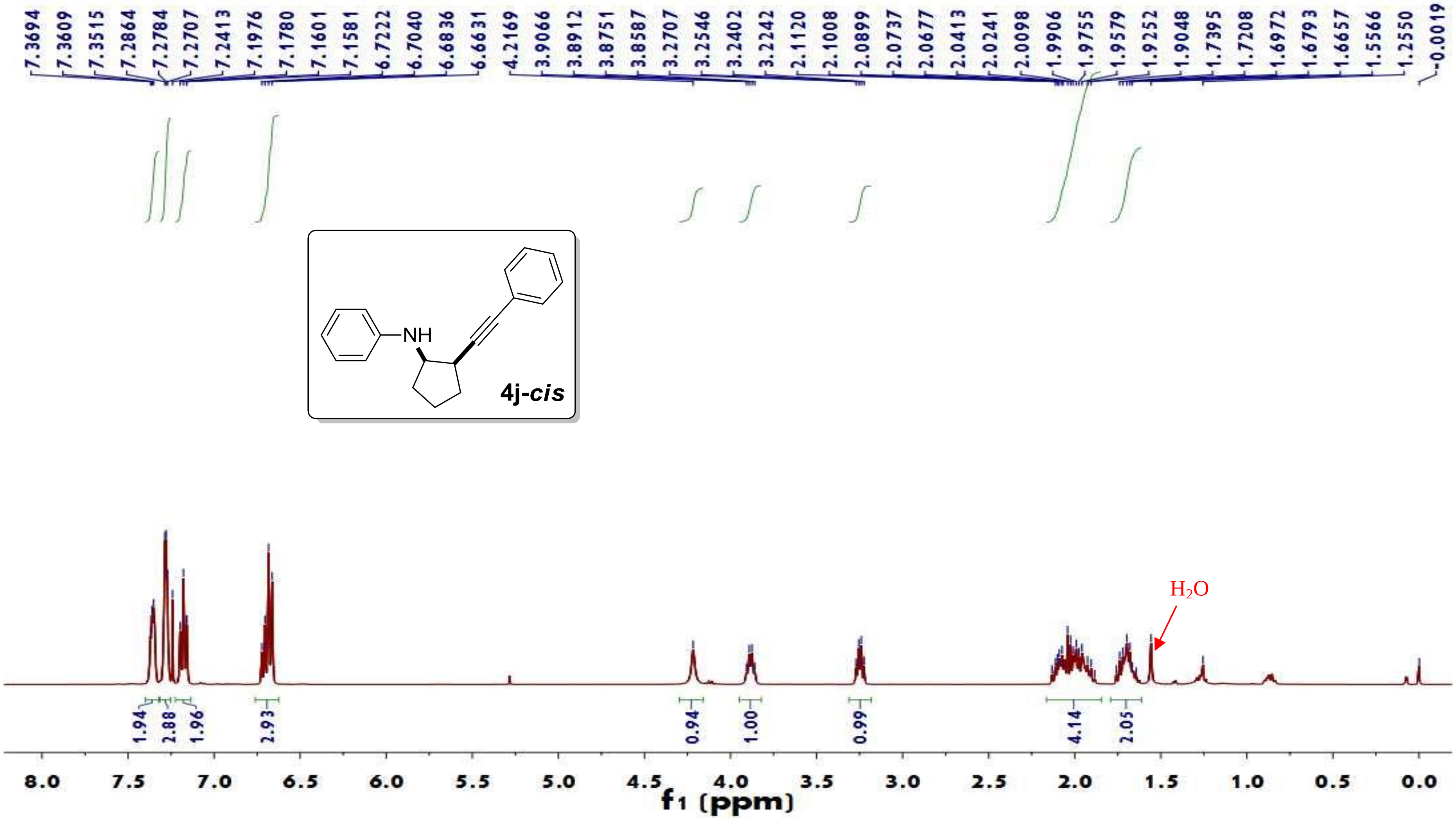

^{1}H NMR spectrum of 4j-*cis* (400 MHz, $CDCl_3$).

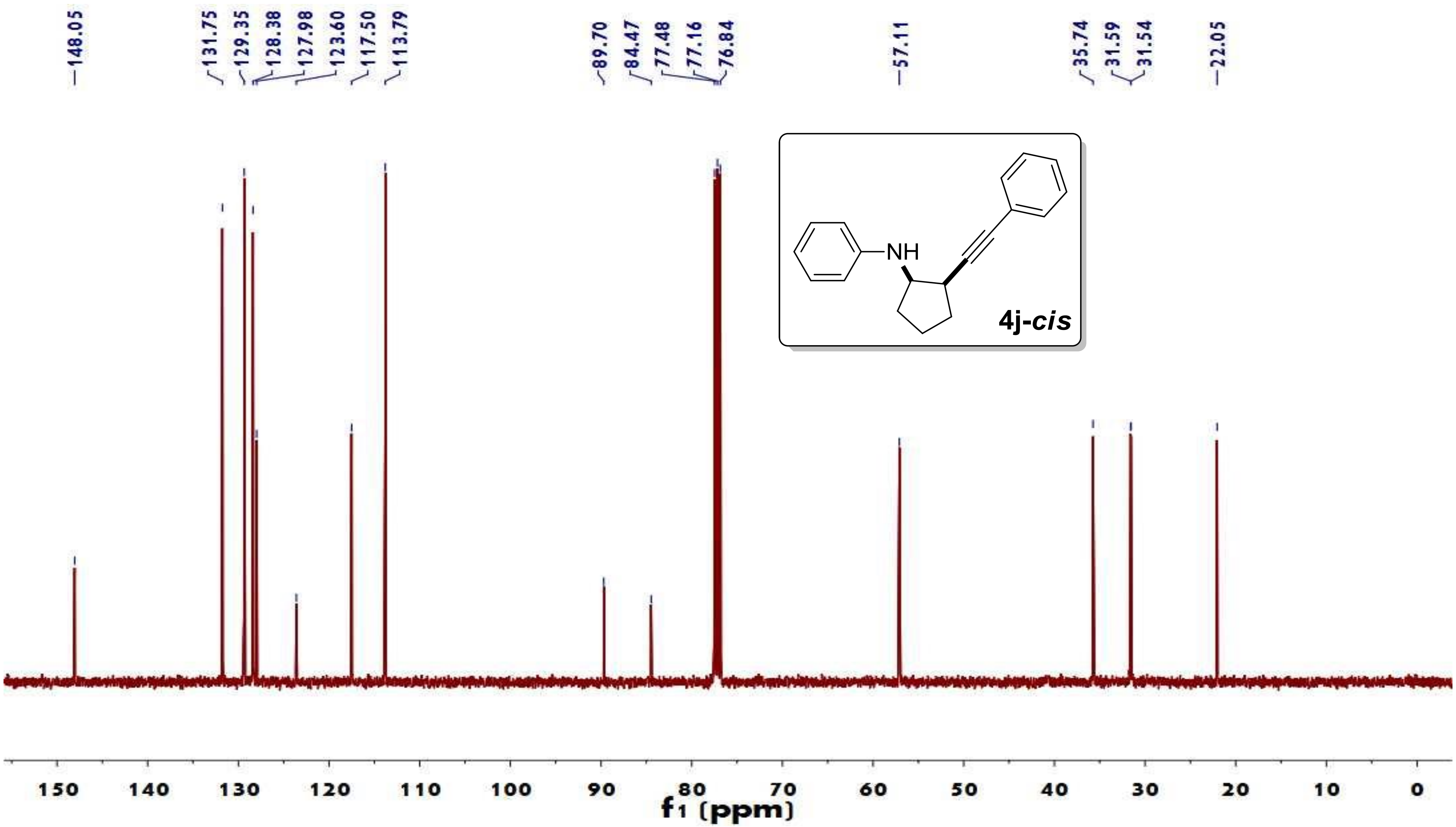

^{13}C NMR spectrum of 4j-*cis* (400 MHz, $CDCl_3$).

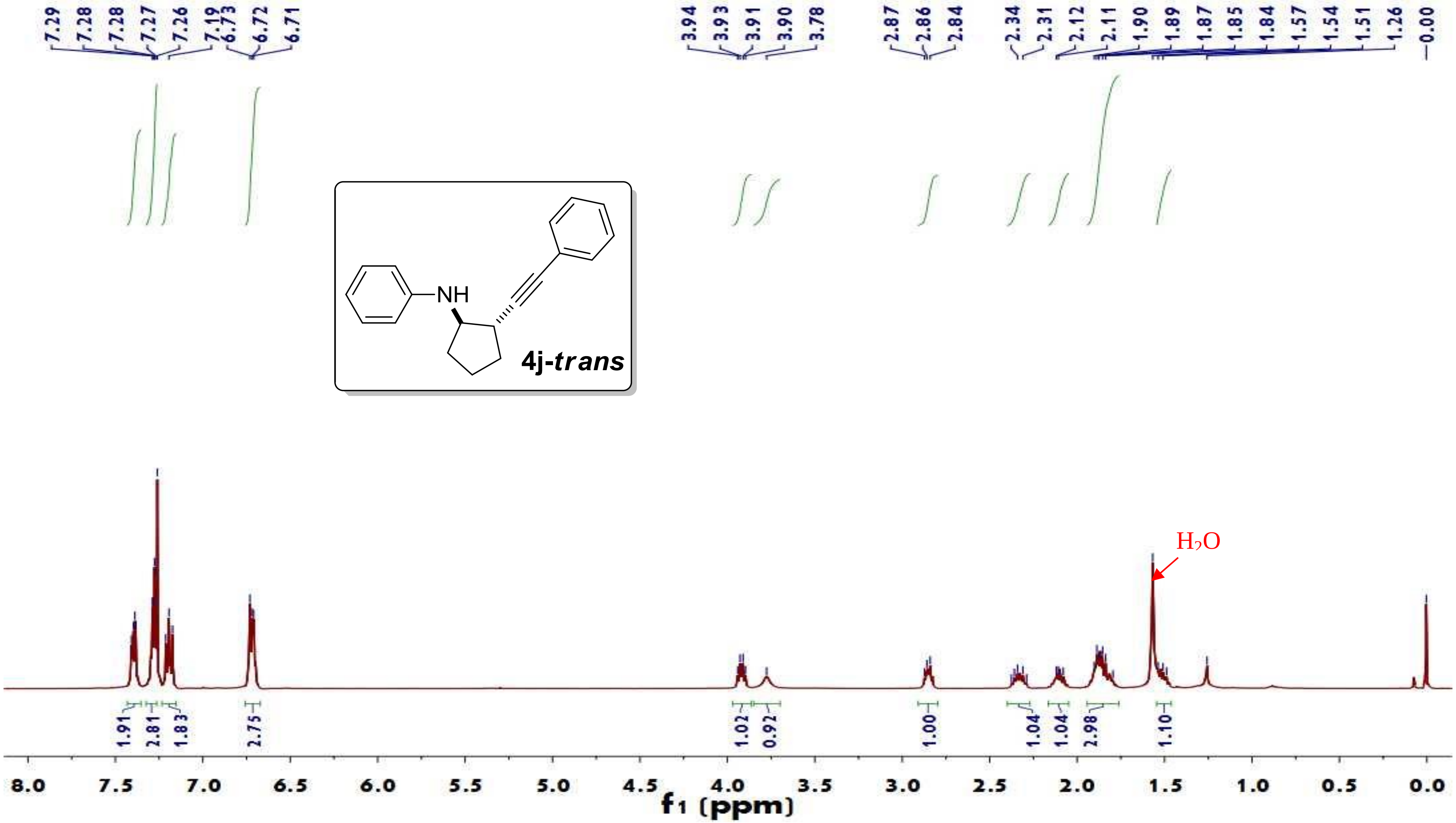

^{1}H NMR spectrum of 4j-*trans* (400 MHz, $CDCl_3$).

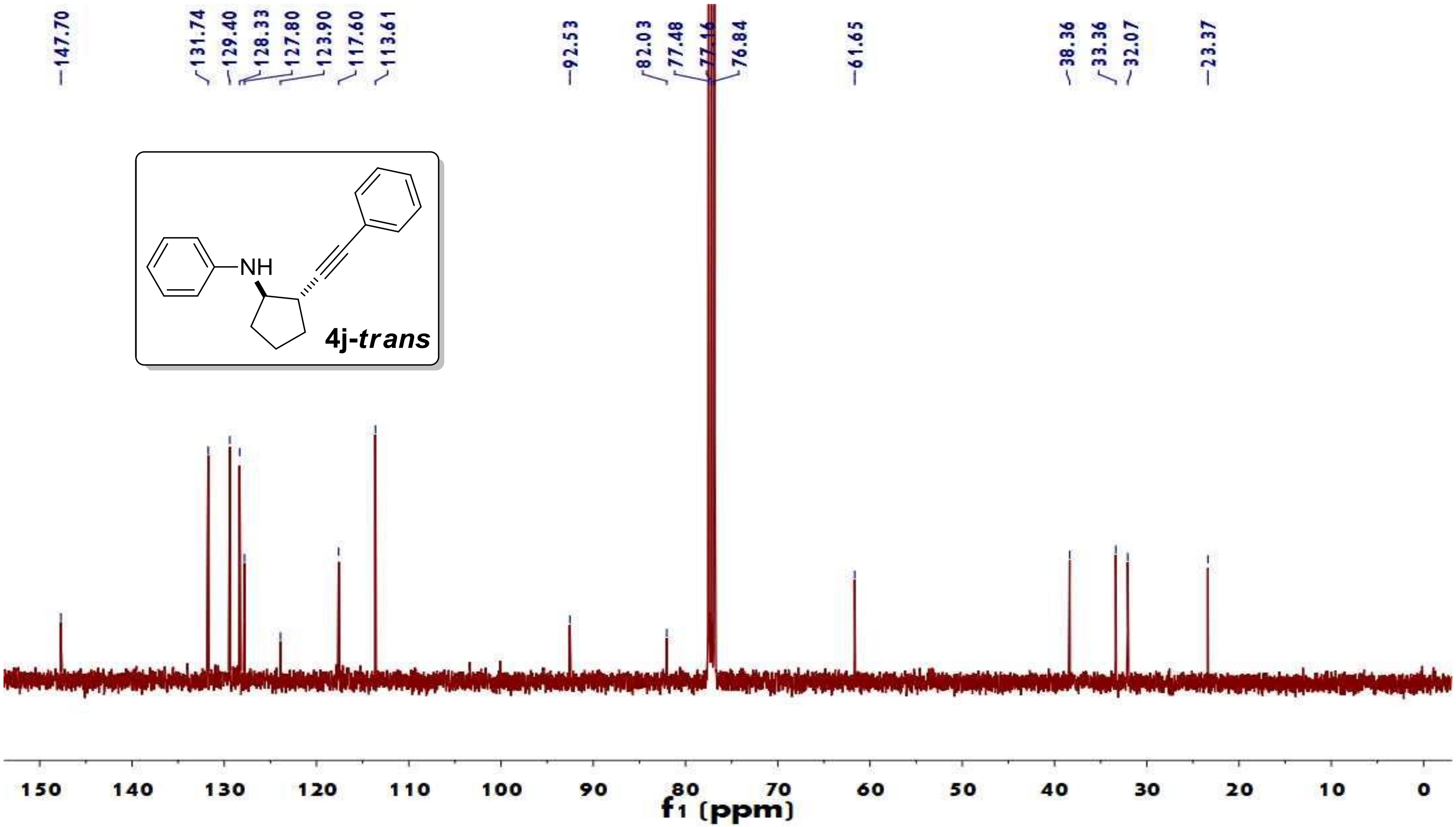

^{13}C NMR spectrum of 4j-*trans* (400 MHz, $CDCl_3$).

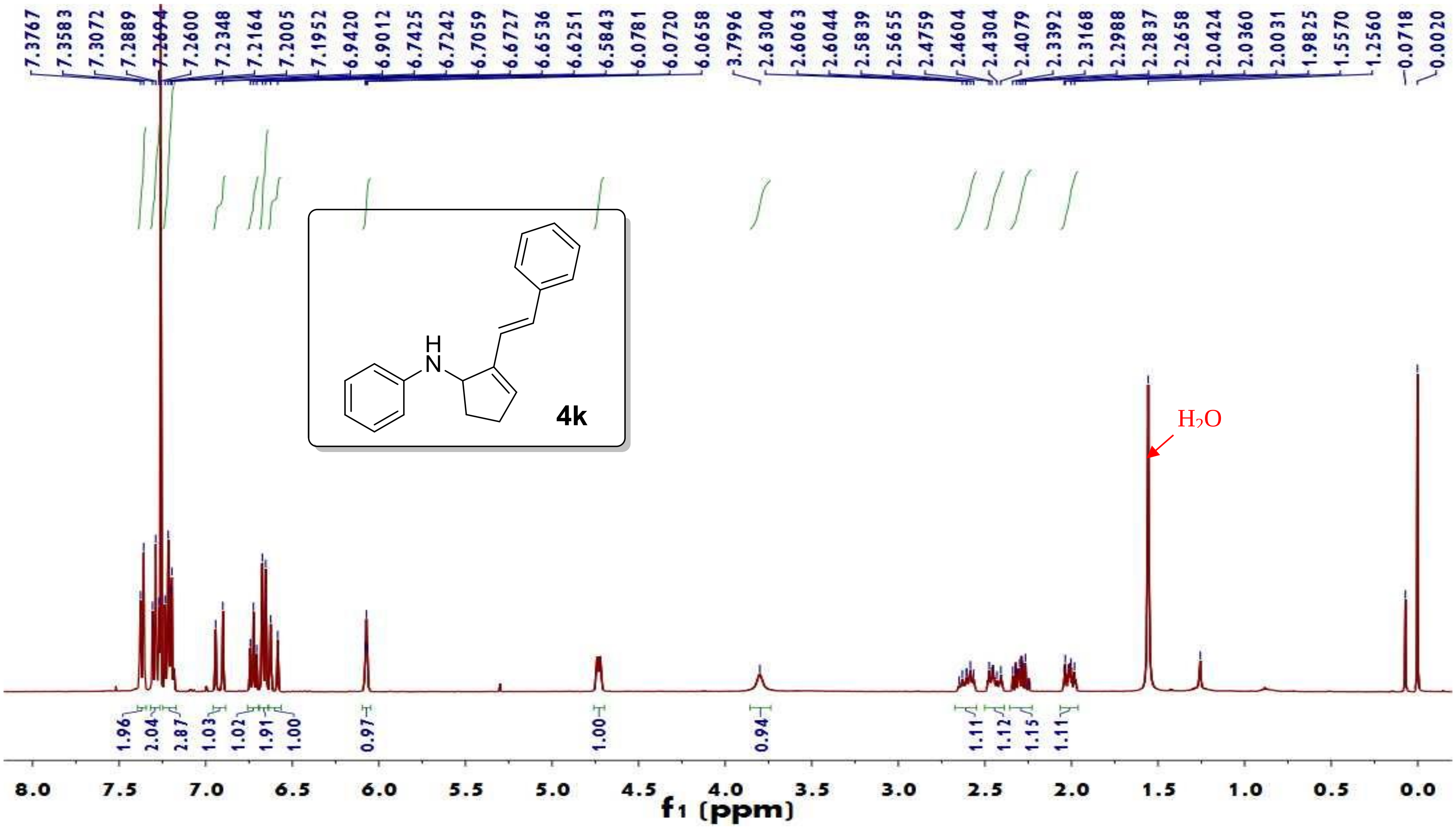

^{1}H NMR spectrum of 4k (400 MHz, $CDCl_3$).

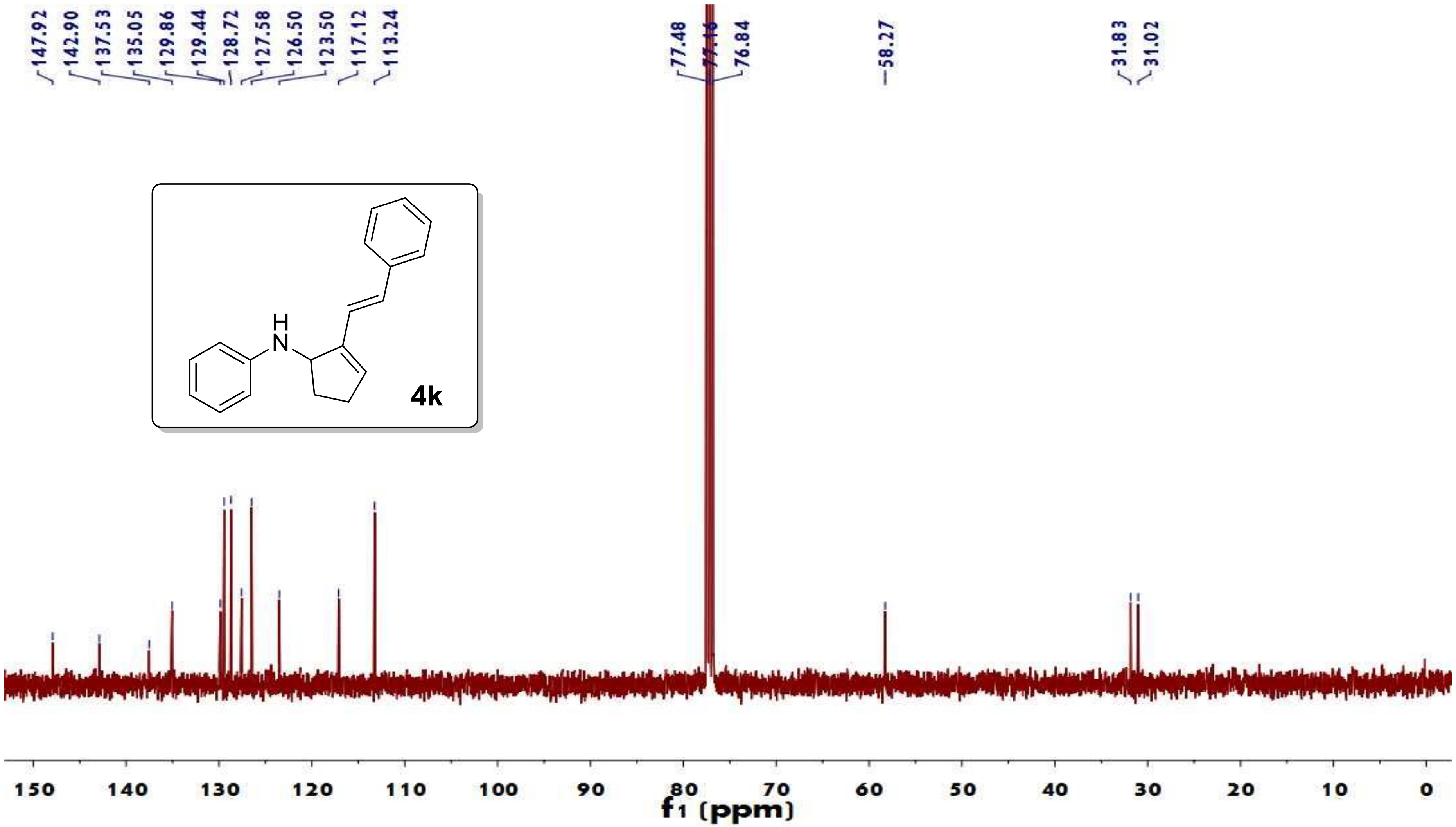

^{13}C NMR spectrum of 4k (400 MHz, $CDCl_3$.

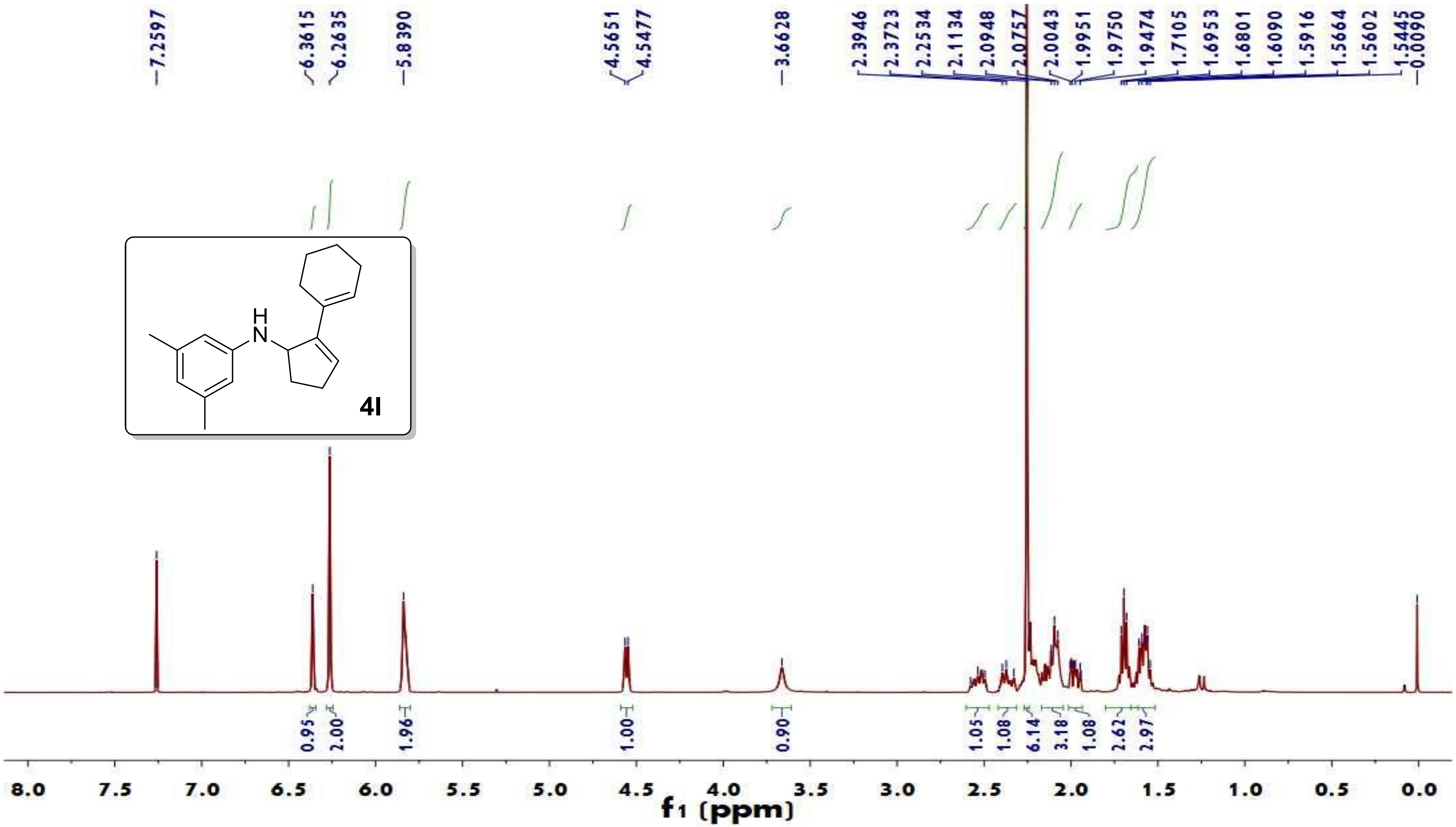

^{1}H NMR spectrum of4l (400 MHz, $CDCl_3$).

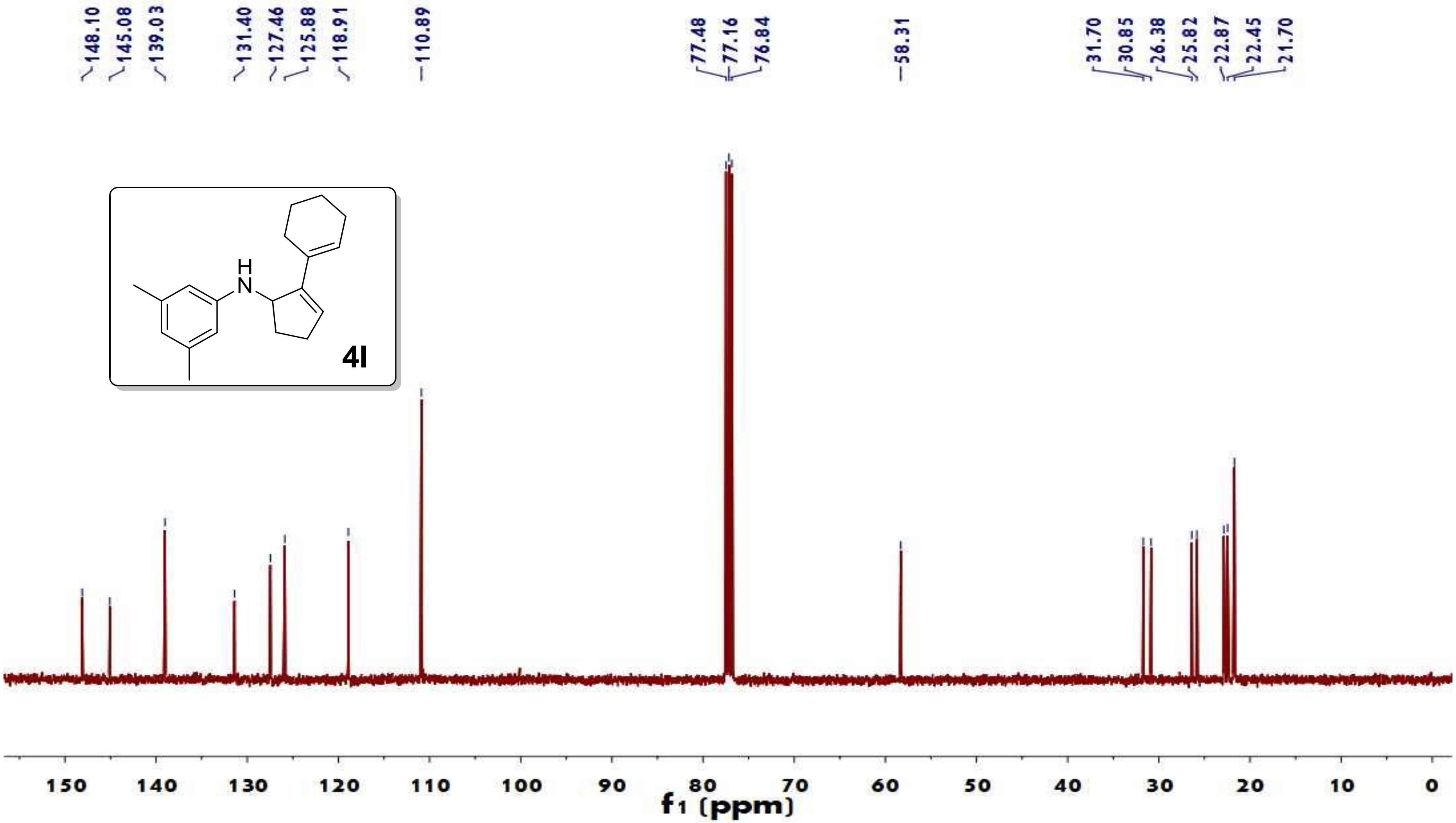

^{13}C NMR spectrum of 4l (400 MHz, $CDCl_3$).

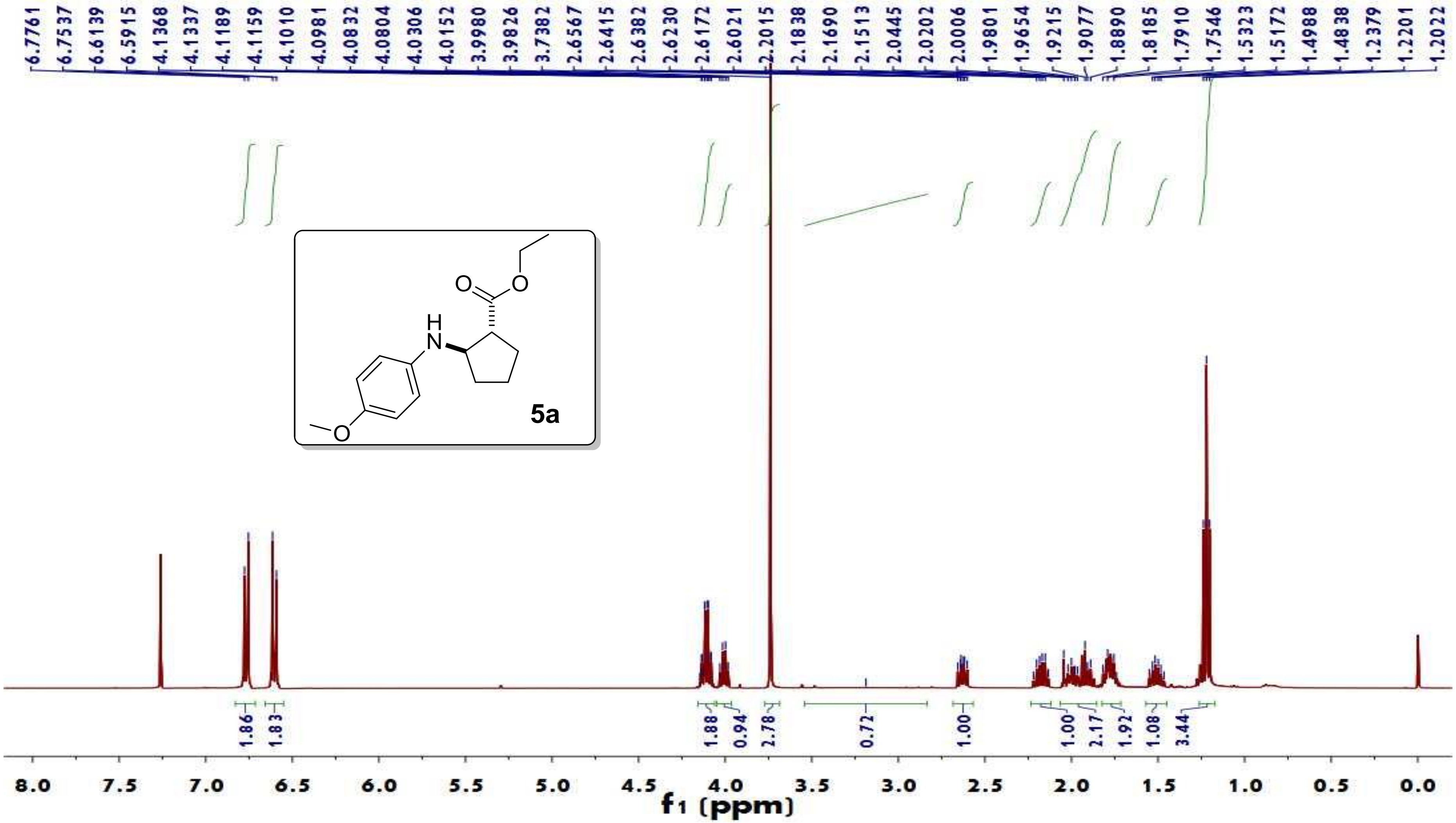

^{1}H NMR spectrum of 5a (400 MHz, $CDCl_3$).

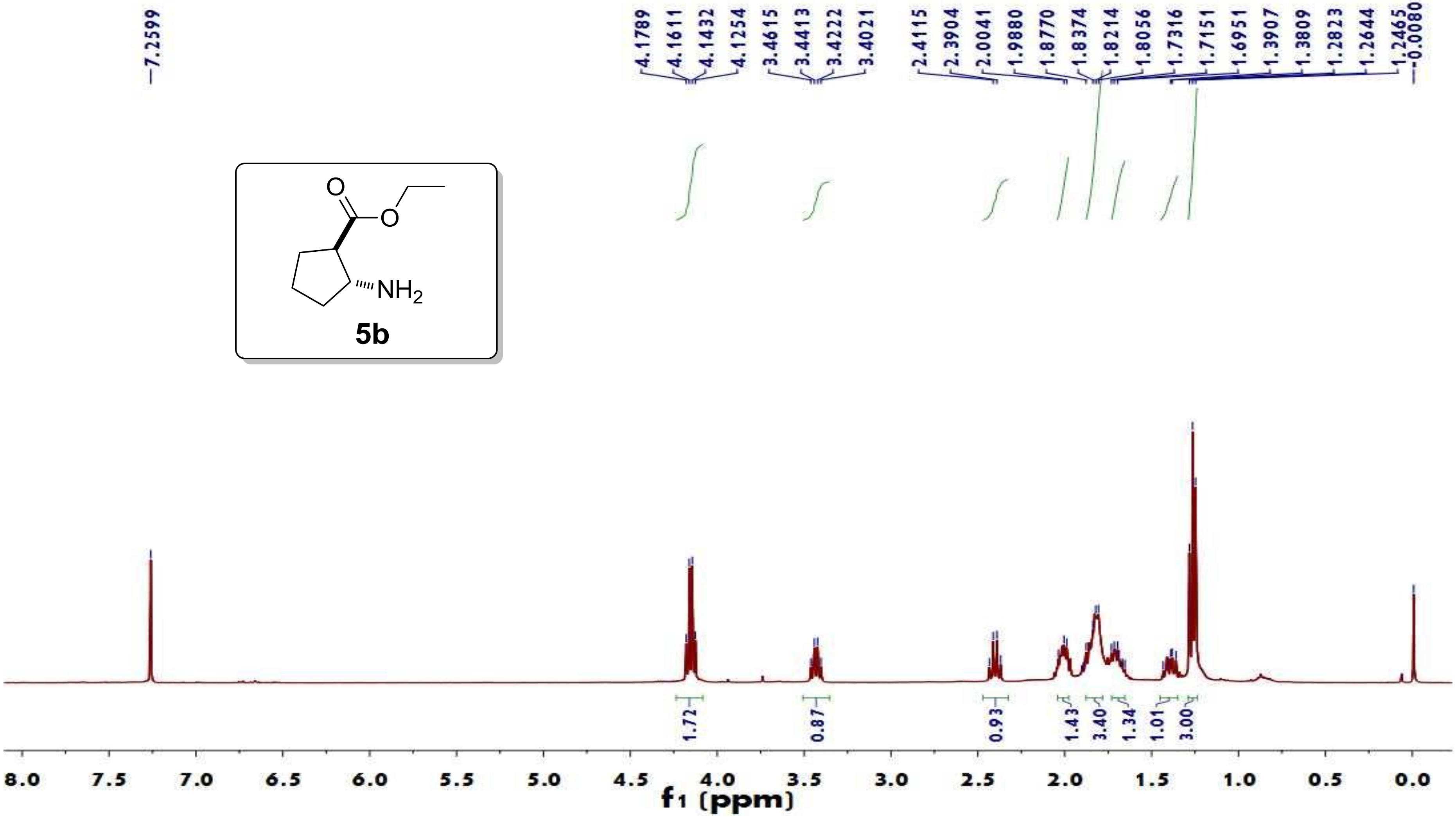

^{1}H NMR spectrum of 5b (400 MHz, $CDCl_3$).

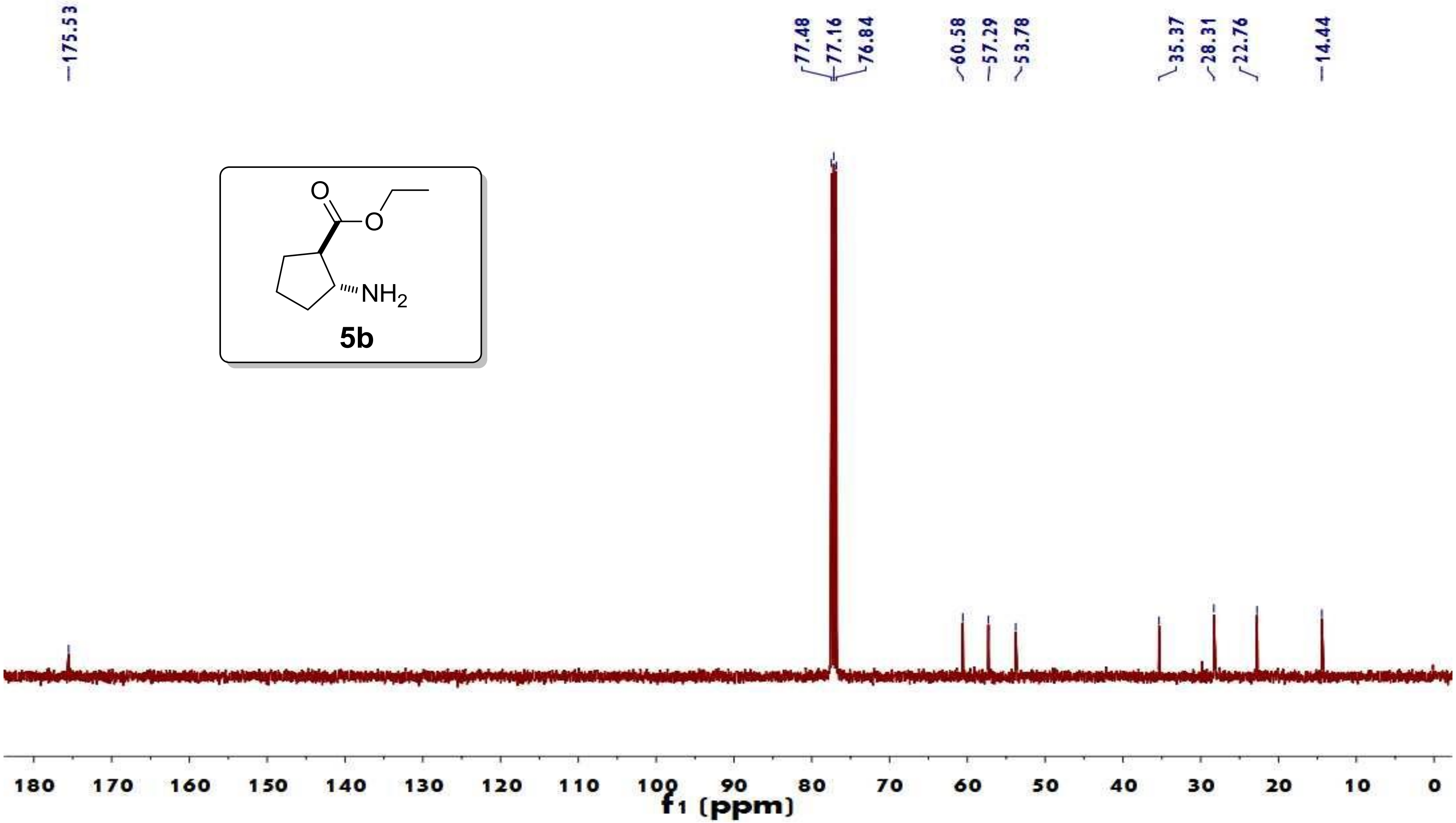

^{13}C NMR spectrum of 5b (400 MHz, $CDCl_3$).
